# Supplementary material for: Spatial control of karyopherin binding avidity within NPC mimics revealed by designer FG-nucleoporins
Source: Nat Commun. 2026 Sep 28;17:11379. doi: 10.1038/s41467-026-77818-0 (PMC13737079; doi:10.1038/s41467-026-77818-0)
Supplement: Supplementary file 1 [file 41467_2026_77818_MOESM1_ESM.pdf]

Supplementary Information

Spatial control of karyopherin binding avidity within NPC mimics revealed by designer FG-Nucleoporins

Hendrik W. de Vries[1*], Anders Barth[2*], Alessio Fragasso[2*], Tegan A. Otto[3], Ashmiani van den Berg[2], Eli O. van der Sluis[2], Erik van der Giessen[1], Liesbeth M. Veenhoff[3], Cees Dekker[2$], Patrick R. Onck[1$]

[1]Zernike Institute for Advanced Materials, University of Groningen, Nijenborgh 3, 9747AG Groningen, The Netherlands.

[2]Department of Bionanoscience, Kavli Institute of Nanoscience, Delft University of Technology, Van der Maasweg 9, 2629 HZ Delft, The Netherlands.

[3]European Research Institute for the Biology of Ageing, University of Groningen, University Medical Center Groningen, Hanzeplein 1, 9713GZ Groningen, The Netherlands

[*]These authors contributed equally.

[$]Corresponding authors: p.r.onck@rug.nl, c.dekker@tudelft.nl

Table of Contents:

Supplementary Table 1 – Amino acid sequences of artificial FG-Nups used in simulations and experiments. 7

Supplementary Table 2 - Overview of FG-domains of *saccharomyces cerevisiae* and selected domains of *homo sapiens*. 7

Supplementary Table 3 – Overview of simulations in this work. 8

Supplementary Table 4 – Hydrophobicity scale used in our 1-BPA model. 8

Supplementary Table 5 – Overview of interaction types. 10

Supplementary Table 6 – Extracted parameters from fitting frequency shifts upon Kap95 binding to NupY brushes. 10

Supplementary Table 7 – Amino acid sequences of Kap95 and cargoes used in the study. 11

Supplementary Table 8 – Sequence charge properties of NupY-mutants. 12

Supplementary Table 9 – sequence charge properties of native GLFG-Nups and NupX 12

Supplementary Table 10 – statistical analysis of −ΔD/Δf across NupY variants. 13

Supplementary Table 11 – pairwise statistical comparisons of −ΔD/Δf across NupY variants. 13

Supplementary Table 12 – logarithmic regression analysis of Kap95 binding parameters across NupY variants. 14

Supplementary Figure 1 – Sequence histograms for FG-spacing variants and C/H-ratio variants. 15

Supplementary Figure 2 – Violin plots of the Stokes radii of 50 different designs for different combinations of C/H-ratio and FG-spacing. 16

Supplementary Figure 3 – Violin plots of the radii of gyration of 5 baseline NupY-designs, compared to Nup100. 17

Supplementary Figure 4 – Inter-residue contact maps for NupY base design variants and Nup100. 18

Supplementary Figure 5 – Overview of QCM-D experiments on NupY brushes. 19

Supplementary Figure 6 – Harmonic spread of final Δf after NupY coating and layer height estimation. 20

Supplementary Figure 7 – Robustness of Kap95 binding to NupY(13;0.05) for brushes with different final Δf. 21

Supplementary Figure 8 – MUTEG coating and NaOH dissociation of NupY-bound Kap95 in QCM-D experiments. 22

Supplementary Figure 9 – Response of NupY-coated QCM-D chips to BSA. 23

Supplementary Figure 10 – Fitting of the QCM-D frequency response upon Kap95 binding. 24

Supplementary Figure 11 – Characterization of the liquid-liquid phase separation propensity and aggregation of the different NupY variants. 25

Supplementary Figure 12 – PEG does not partition into NupY condensates. 26

Supplementary Figure 13 – Minimal interactions of model cargoes with NupY condensates. 27

Supplementary Figure 14 – Diffusion kinetics of Kap95 in NupY condensates. 28

Supplementary Figure 15 – Snapshots of nanopores coated with FG-spacing and C/H-variants. 29

Supplementary Figure 16 – Time-averaged, axi-radial density graphs of FG-Nups in nanopores coated with artificial FG-Nups, grouped by C/H and FG-spacing. 30

Supplementary Figure 17 – Time-averaged, axi-radial density graphs of F(G)-motifs in nanopores coated with artificial FG-Nups, grouped by C/H and FG-spacing. 31

Supplementary Figure 18 – Time-averaged, axi-radial density graphs of FG-Nups in nanopores coated with artificial FG-Nups in presence of Kap95 and inert proteins, grouped by C/H and FG-spacing. 32

Supplementary Figure 19 – Time-averaged, axi-radial density graphs of the F(G)-motifs in nanopores coated with artificial FG-Nups in presence of Kap95 and inert proteins, grouped by C/H and FG-spacing. 33

Supplementary Figure 20 – Time-averaged, axi-radial density graphs of the Kap95 protein in nanopores coated with artificial FG-Nups in presence of inert proteins, grouped by C/H-ratio and FG-spacing. 34

Supplementary Figure 21 – Time-averaged, axi-radial density graphs of the BSA protein in nanopores coated with artificial FG-Nups in presence of Kap95 and inert proteins, grouped by C/H and FG-spacing. 35

Supplementary Figure 22 – Time-averaged, axi-radial density graphs of the Ubiquitin protein in nanopores coated with artificial FG-Nups in presence of Kap95 and inert proteins, grouped by C/H and FG-spacing. 36

Supplementary Figure 23 – Time-averaged, axi-radial density graphs of the entire protein mass (FG-Nups and inert proteins and Kap95) in nanopores coated with artificial FG-Nups in presence of Kap95 and inert proteins, grouped by C/H-ratio and FG-spacing. 37

Supplementary Figure 24 – Occupancy and on-axis velocity magnitude of Kap95 38

Supplementary Figure 25– SDS-PAGE gel of purified NupY variants and Kap95. 39

Supplementary Figure 26 – Contour graphs of cargo mass flux and NupY nanopore selectivity 40

Supplementary Figure 27 – Equilibration of dissociation constant and in-pore protein density 41

C/H	d_{FG}	
**0.0478 (1x)**	**7 (0.5x)**	**GPSQIMNSTFGPNNTGNQGLFGAPGSPSTFGTNQTNGTGLFGQAQSSSNFGQGSPLTTGLFGLSLAPQEFGNSAMVQS<br>FGPNTGASTGLFGRKGGQKSFGKFNNIMMGLFGQSSPQTQFGQNSANNTGLFGQALTTGAFGQPFSANNFGLGNNPTS<br>GLFGNGGKVGTFGSGSPGNPGLFGNSGNGQTFGLGLKTGSGLFGNGNHKTSFGAAGASLNFGQNQAGSNGLFGKGGAA<br>SNFGSSKQSKNGLFGMQSSGSTFGAQGNNQQGLFGTGGSTTNFGHHQSSTMFGTMINNQQGLFGNQNNLTQFGNTSSQ<br>PSGLFGHTQMFGKFGTNVNPLVGLFGGGQPMTSFGAGNIMGQFGTNSQGGSGLFGQVTNGMNFGHSMLNSMGLFGTAN<br>SGPSFGMNSSHSTGLFGQIVLPGTFGNGGASHNFGNQNSNSQGLFGMMSSDGNFGPTQSQTSGLFGQTLTPGPFGSGQ<br>HASSGLFGMNNGSFQFGAGTTNMQFGNQTGTNSGLFGQGSLAPNFGSPKASTAGLFGSGATAGTFGSSLSNTTGLFGF<br>QYGAGSFGPTSMSGQFGNPPQSTAGLFGAPGGTAPFGNNSAGKSGLFGPSRPNQHFGSPNNTTTGLTKLKSSIGKDFT<br>LAQVNKTTNNASIIEAIPYYNLVKKSSNKRRSYNEDDPRLGENASSAKAKKFQLDREQLNSAGKDDFDIQLLSPHESV<br>FMIMLNAGNLDARLDANNKVESLNSNDAQRSNGENPKPRAMTPLTISSEKDSISSSMKIDPAKHLTEPSQDIEQKKLT<br>TTWKSSDEKPVSVPKSTNSSEEC**
0.1912 (4x)	7 (0.5x)	GPSQIMNSTFGGNKTGNQGLFGAPGSPSTFGTKQANGTGLFGQQQSSSNFGQGKPTTTGLFGKSLAPQEFGESAMHQS<br>FGHNTGASTGLFGRKEGQKSFGKFNNISMGLFGQSSPQTQFGQNSGNNTGLFGQALTTGAFGSRFSAKRFGLGENPTS<br>GLFGRGGKVGGFGSESGRDHGLFGDSENGQTFGLGLKTGSGLFGNGEHKTSFGATGAELNFGQNGAGSRGLFGKDRAP<br>ENFGSSKQGKNGLFGMHSGGSTFGEQGNNQQGLFGQGESETNFGMHQSSKMFGTGINDQQGLFGEQNNLTQFGNTSSQ<br>PSGLFGGTQGFGKFGTNVNPLPGLFGGGQPPTSFGARNIGGSFGKNSQGGRGLFGQSDNGMNFGGSMLNSMGLFGTAN<br>GGPSFGMNSGHSTGLFGQIVLPGGFGNKDSSTNFGNQNSNSQGLFGPMSSDGNFGPTQSETSGLFGQTLTPRPFGSGQ<br>HASSGLFGGENGGFQFGARTTNGHFGNQTGTNSGLFGQGSLAPKFGSPKTSTAGLFGSKATAATFGSSDKRTTGLFGF<br>QHGAGSFGSGSMSGQFGNPPQGTAGLFGSPEGRAPFGNASHRKSGLFGPSRPNQHFGSPENTTTGLTQLKSSIGKDFT<br>LAQVNKTTNNASIIEAIPYYNLVKKSSNKRRSYNEDDPRLGENASSAKAKKFQLDREQLNSAGKDDFDIQLLSPHESV<br>FMIMLNAGNLDARLDANNKVESLNSNDAQRSNGENPKPRAMTPLTISSEKDSISSSMKIDPAKHLTEPSQDIEQKKLT<br>TTWKSSDEKPVSVPKSTNSSEEC
0.3824 (8x)	7 (0.5x)	GPGQIMNRGFGPKDPGNSGLFGAPESPSTFGTNMTNGDGLFGESQESSEFGTDSHLREGLFGGDAAPQEFGNSMDQQS<br>FGPKSGKSRGLFGRKRRKKSFGKHNKIMPGLFGQSSTQTDFGQKSEENTGLFGQAETTGHFGQPKSADRFGSGENPTS<br>GLFGNGGKVGGFGKGSPGEPGLFGNKDNGQTFGSEMKTGEGLFGNGNHKTSFGAAGSSLNFGSNTKGSKGLFGKGGKK<br>DNFGSSKQDKEGLFGHQSSGDTFGEQEMNQQGLFGKGGSTKRFGDHQSSTTFGTQHSEQGGLFGDHNNHTQFGNRSSQ<br>PRGLFGHKQPFGKFGGNVEPPVGLFGKKEPASSFGEGNHMEQFGGDSSGGQGLFGQHGRRPNFGHSMLNRMGLFGTAN<br>SKPSFGTNDSHKKGLFGQIMMPKTFGNRKAQTNFGNDNSNSQGLFGMMSSDGNFGDDQRQKSGLFGQGMTPGPFGSEQ<br>HDSSGLFGMDKGGFHFGDDSTPMQFGEGGHGNSGLFGQGSLDMKFGGTKTSTTGLFGSGRKHGSFGSGLSNGTGLFGF<br>GSAAGRFGPTGMEGQFGNPRQSGAGLFGAPGETHSFGRKSAGKSGLFGRDRPRQKFGSPNNDTTGSTKLKSSIGKDFT<br>LAQVNKTTNNASIIEAIPYYNLVKKSSNKRRSYNEDDPRLGENASSAKAKKFQLDREQLNSAGKDDFDIQLLSPHESV<br>FMIMLNAGNLDARLDANNKVESLNSNDAQRSNGENPKPRAMTPLTISSEKDSISSSMKIDPAKHLTEPSQDIEQKKLT<br>TTWKSSDEKPVSVPKSTNSSEEC
**0.5736 (12x)**	**7 (0.5x)**	**GPSEMHNSDFGGDERRETGLFGAEGSPSSFGTNGTNRKGLFGQGGRSSEFGQRRGQRTGLFGAKEGGQEFGESAQMQS<br>FGSNTGRSSGLFGRKGGQKSFGKFRNIKMGLFGSDGPRTQFGQNEAKKKGLFGQRMSERSFGQPMKGNNFGQKENKDD<br>GLFGNKKKAGMFGKGSTKNPGLFGDSGEGEKFGLDAKDGSGLFGNGKGKSSFGAAGAGHEFGQEQAGKKGLFGKKEDA<br>SAFGSKKQDKEGLFGSEESGETFGRQRNESQGLFGTGEKKENFGDHQSEDQFGTPKNNQGGLFGHGKDLSQFGETSEG<br>TDGLFGHTKMFGKFGREQNPLEGLFGGGEHAGPFGTGEPMGQFGTRESGGSGLFGQVDNGGEFGHRTLRSHGLFGTTN<br>SDPEFGMNSSHRSGLFGQGELSRRFGDGDHTGNFGNGNSKSGGLFGRTDEDRKFGTGQSQDGGLFGQRGTKESFGSRS<br>EAKKGLFGDENDSKQFGPRQDKMQFGNQSGSKSGLFGSGGHAPNFGRPKQDGKGLFGSGATAKKFGSSHRKTTGLFGR<br>TSGAKSFGGTSKSEMFGDDPQDSPGLFGAPGDTSPFGEESSKKEGLFGPKRTNQEFGKPKNETTGSTKLKSSIGKDFT<br>LAQVNKTTNNASIIEAIPYYNLVKKSSNKRRSYNEDDPRLGENASSAKAKKFQLDREQLNSAGKDDFDIQLLSPHESV<br>FMIMLNAGNLDARLDANNKVESLNSNDAQRSNGENPKPRAMTPLTISSEKDSISSSMKIDPAKHLTEPSQDIEQKKLT<br>TTWKSSDEKPVSVPKSTNSSEEC**
0.0239 (0.5x)	13 (1x)	GPTPGPSANSHAMGMFGAASHASTNSFSGSGLFGSNTQMAANMLTQMFGQPAGSNNTMNTSQGLFGGMNQTFMVKMSS<br>NFGNGNGRGQNQQAMLGLFGQPQTHPTGSIITHFGGLSTSKSQSSQVAFGSSTQMAGMHMQTNGLFGSMNNNTNLSMN<br>PMFGTAVSQAAVASYAMGLFGISSGHTQTGAQNVFGPQPAANSPQSMTGGLFGHANGTTKANMNQPFGAAFSPAGSMA<br>FPNFGQLMANTANGPMGAGLFGPPNQTASPSNTPPFGQNPLPPVPQTQAPGLFGSAQSHANTTNGNMFGHTMSGTKGN<br>MINAGLFGQPQNQGGMQTTQIFGTALPAPMQMMNSQFGSNRTMAAHMQATGGLFGALMQSSLLNPQATFGQSNQASHL<br>SMQMMGLFGAGAMTTQSNSNMMFGGSQTAQPSSMGNMGLFGTHMAGPALAMKMGFGMSQQSMPFTGGQVFGLNLQQMQ<br>QGTNTTGLFGTNVLTMAFQAPQPFGGKLGGSSQSNSNLGLFGSQTMGTPLGQNSNFGAGQQSPNSSATGGGLFGANSP<br>MSPANGQATFGQNGNAMMMPMGKPFGTQTTMNNASSNGIGLFGAFANSQQAPMSNTFGSTQPTSASQNLKSSIGKDFT<br>LAQVNKTTNNASIIEAIPYYNLVKKSSNKRRSYNEDDPRLGENASSAKAKKFQLDREQLNSAGKDDFDIQLLSPHESV<br>FMIMLNAGNLDARLDANNKVESLNSNDAQRSNGENPKPRAMTPLTISSEKDSISSSMKIDPAKHLTEPSQDIEQKKLT<br>TTWKSSDEKPVSVPKSTNSSEEC
**0.0478 (1x)**	**13 (1x)**	**GPSSGPSANSGGTNQFGTASTSGNNNFSGSGLFGSNTQPAKNQLSTNFGQPAGSNNKMNTSQGLFGGQNGTFPVNSGS<br>NFGNNNGRGQNQQAQLGLFGSPQGTGTGSIITNFGGLGGSKSQGSQVQFGSSTQASNPNTQTNGLFGSQNNNTNLSTN<br>SQFGTAVSQSAVASYAKGLFGISSGNTQTGAQNVFGTQNAANSNTSTNGGLFGNANGNNKANTNQPFGAAFSQAGSQT<br>FPNFGQLAPNTANGPQGAGLFGQGNQTSSPSNSSAFGTNTLPGVPQTQKSGLFGSANSSANGENGNMFGNGTSGTKGN<br>PINAGLFGQPSNKGGKQTTSIFGTALGAGSQNSNSNFGSNRTMTSGSQATNGLFGQLQTSGLLNTQTTFGQSNTSSGL<br>SGQMGGLFGAGTQTNQSNSNASFGGSQTTQPSSAGNNGLFGTSAGGNPLATNQKFGTSQQSPQFTNGQVFGLNLQNTG<br>QGTNTTGLFGTNVLNKSFQAPQNFGNKLNGSSNSNSNLGLFGSGTTGTPLGQNSNFGAGQQSPNSSATGGGLFGSNSP<br>ASQTNGSATFGTNGNNGMMPMGKTFGTSTNNNNANSNGIGLFGTFNNSQQTPNGNTFGSTQNTNPSTNLKSSIGKDFT<br>LAQVNKTTNNASIIEAIPYYNLVKKSSNKRRSYNEDDPRLGENASSAKAKKFQLDREQLNSAGKDDFDIQLLSPHESV<br>FMIMLNAGNLDARLDANNKVESLNSNDAQRSNGENPKPRAMTPLTISSEKDSISSSMKIDPAKHLTEPSQDIEQKKLT<br>TTWKSSDEKPVSVPKSTNSSEEC**
**0.0956 (2x)**	**13 (1x)**	**GPSSGPSANSGGTNQFGTASTSGNNNHSGSGLFGENTQPAKNQLSTNFGQPAGSRNKMNTSQGLFGGQNGTDPVNSGS<br>NFGNNNGRGQNQQAQLGLFGSPQGTGTGSGITNFGGLGGSKSQGGQVKFGSSTQASNPNTQTNGLFGSRNNNTNLSTN<br>SQFGTAVSQEAVASYAKGLFGISSGNTQTGAQNVFGTQNAANSNTETNGGLFGNANGNNKANTNQPFGAAFSQAGSQT<br>FPNFGQLAPNTANGPQGAGLFGQGNQTSSPSNSSAFGTNTLPGVPQTRKSGLFGSANSSANGENGNMFGNGTSGTKDN<br>PINAGLFGQPSNKGGKQTTSIFGTALGAGSQNSNSNFGSNRTMTSGSQATNGLFGQLQTSGLLNTQTTFGQSNTSSGL<br>SGQMGGLFGAGTQTNQSNSNASFGGSQTTQPSSAGNNGLFGTSAGGEPLATNQKFGTSQQGPQTTNGSVFGSNAQNTG**

		**SGTNTDGLFGTNMLNKSFQAPQKFGNKLNGSSNSNSNLGLFGSKTTGTPLGQNSNFGAGQQSPNSSATGGGLFGGNSP<br>ASQTNGSATFGTNGNNGMMPMGKTFGTSTNNNNANSNGTGLFGTFNDSQKSPNGNTFGSTQNTNPSTNLKSSIGKDFT<br>LAQVNKTTNNASIIEAIPYYNLVKKSSNKRRSYNEDDPRLGENASSAKAKKFQLDREQLNSAGKDDFDIQLLSPHESV<br>FMIMLNAGNLDARLDANNKVESLNSNDAQRSNGENPKPRAMTPLTISSEKDSISSSMKIDPAKHLTEPSQDIEQKKLT<br>TTWKSSDEKPVSVPKSTNSSEEC**
**0.1912<br>(4x)**	**13<br>(1x)**	**GPSSGPSANSGGTNQFGTASTSGNNNFGGSGLFGSNSQAAKNQASTNFGQPAGSNNKMNTEQGLFGGQNKSFPVNSGS<br>RFGNNNHRGQNQQAQPGLFGGPQGTETGSIITPFGGLGEDKSQGSQVQFGSSTRASNPNTQKNGLFGSQNNNSNMSSN<br>SQFGTAVSTSAVASYEKGLFGISSGNSHTGMQGVFGTQNMANSNTSTPGGLFGNENGNKKANSDSPFGAAESQGGSQS<br>FPNFGQMKPNTANGPQDHGLFGHGNQTSRPRNSSGFGTNTLPGVPSTQKSGLFGSANSEAEKENGNMFGRGSSGRKGN<br>PIKAGLFGQPSNKGGKQTTKMFGTALGAGSQNSNSNFGGNRTMTSGSQADNGLFGGLQSSGGLNTQTTFGQQNTEEGL<br>SGQMGGLFGAGTQTNQGNSNASFGGPQTKQPSSAGNNGLFGTSAGGNPLAGNQKFGTSQQSPQFTNGQTFGLNRQNTD<br>GGTDSSGLFGGEVQNKSFQPPQNFGNKLNKSSNSNSNLGLFGSDTTGTPLGQNSAFGAGQQSPNSKGTGEGLFGRESP<br>RSQTNGSATFGTNGNNGMMPMGKSFGSSTNNNKPNSNGIGLFGTFNNSQQTPNGDTFGSKHRTNPSDNLKSSIGKDFT<br>LAQVNKTTNNASIIEAIPYYNLVKKSSNKRRSYNEDDPRLGENASSAKAKKFQLDREQLNSAGKDDFDIQLLSPHESV<br>FMIMLNAGNLDARLDANNKVESLNSNDAQRSNGENPKPRAMTPLTISSEKDSISSSMKIDPAKHLTEPSQDIEQKKLT<br>TTWKSSDEKPVSVPKSTNSSEEC**
**0.3824<br>(8x)**	**13<br>(1x)**	**GPSEGPSAKKGGTKGFGTSSTSGNNDFSGEGLFGSNEQPAKNQGATNFGQPQKSRNKTETSQGLFGKSNEGFPSNGGS<br>DFGNENGRGQNQGSELGLFGKTQGEGTGKIITRFGGLGGSKSSESQSSFGSSPSASNPNTQSNGLFGDRNENTRRSTN<br>SSFGTAVSQKAPASSAKGLFGISSGNTQDERTNVFGDQNAADDNTETEGGLFGNMNERNKANTNHPFGAAPSQHGSQG<br>FPEFGQAQQNTANKQTDAGLFGHRERTSSPKKSDAFGTNTLKEVSQTGKSGLFGSHNSSANGEKGNMFGNGESGTKGN<br>PKEAGLFGQSKNKGKKQKGSIFGTATGAGSQRSNEGFGSNRTDDEGSQGTNGLFGQEQTSELLNDQSTFGQSNTSGEL<br>SGGMGGLFGTGTHTEKSNSNASFGDGQTKQPKSTGNNGLFGTSTKGNQLSTNQKFGGSQQSPTTTNGHVFGMNGHRTG<br>GGTRTTGLFGEKVKNKGFQAPQDFGNKHEDSSNSNSNAGLFGSGRTKRPLGSNSNFGTGQQRPNRSQSGGGLFGRKRT<br>GSQREGSAGFGTNGNNGMMPMGKTFGTSENNNNANSEGQGLFGSFNNSQETPNGNTFGSKGNTKGETNLKSSIGKDFT<br>LAQVNKTTNNASIIEAIPYYNLVKKSSNKRRSYNEDDPRLGENASSAKAKKFQLDREQLNSAGKDDFDIQLLSPHESV<br>FMIMLNAGNLDARLDANNKVESLNSNDAQRSNGENPKPRAMTPLTISSEKDSISSSMKIDPAKHLTEPSQDIEQKKLT<br>TTWKSSDEKPVSVPKSTNSSEEC**
0.5736<br>(12x)	13<br>(1x)	GPKKRPSTRDGRTNQFGTASGSKNNNKSDSGLFGSNTHPTKNSLDRRFGGRAEDNDKMDTSEGLFGGKEGTTPDNSGS<br>NFGRDKERGQEDQAKPGLFGSPTGDMTGSSTSEFGRLRKSKSKDRQPQFGSSDGRSDGKTQTRGLFGEEDNNTNLSKN<br>KEFGTAVGSGDVGSKAKGLFGSSSGETQTDAQNGFGTQNHANSEEGTNEGLFGNANMNRKARTNQSFGGAFKQAGSQE<br>QPKFGGLAQEGGNGSSKAGLFGQGKQGSSPENSSDFGENRSPEVRKTQKSGLFGSANSSANDENDKMFGNGTPKTKQN<br>PINAGLFGQDENSKEPQDTSIFGRPLRQGSRNSNSNFGGNRTMRSGGHASNGLFGGLTTSGLGNTQRTFGHERTSSGK<br>GGQRKGLFGAGTQRRQSKSRAGFGGDETEKPSKTRNRGLFGGSQGGRPKRSNQKFGSSSQSQQATREQVFGTNAQNTE<br>QGTNTTGLFGTNPLEKDFQQSGRFGNKQRESSNGRGNLGLFGTEMTDEALGQNSRFGAGTQDPNGKRDGDGLFGSPKP<br>ASSTEDSATFGENGNERSHPMGKDFGSGGENNNHESEEIGLFGTHNESEHKRKGNKFGSEQPENPSTNLKSSIGKDFT<br>LAQVNKTTNNASIIEAIPYYNLVKKSSNKRRSYNEDDPRLGENASSAKAKKFQLDREQLNSAGKDDFDIQLLSPHESV<br>FMIMLNAGNLDARLDANNKVESLNSNDAQRSNGENPKPRAMTPLTISSEKDSISSSMKIDPAKHLTEPSQDIEQKKLT<br>TTWKSSDEKPVSVPKSTNSSEEC
**0.0478<br>(1x)**	**26<br>(2x)**	**GPSSGPSANSGGTNQAATASTSGNNNFSFGSVISSNTQPAKNQLSTNVSQPAGSNNGLFGSQSLMPGQNGTFPVNSGS<br>NGSNNNGRFGNQQAQLLNTQSPQGTGTGSIITNMPGGLFGKSQGSQVQSASSTQASNPNTQTNPMLFGQNNNTNLSTN<br>SQVSTAVSQSAVASYAGLFGSISSGNTQTGAQNVGQTQNAANSNTSFGGKSVLNANGNNKANTNQPQNAAFSQAFGQT<br>FPNIGQLAPNTANGPQGATVMPQGGLFGSPSNSSAMQTNTLPGVPQTQKSSVLGFGNSSANGENGNMTNNGTSGTKGN<br>PINAGLFGQPSNKGGKQTTSIQTTALGAGSQNSNFGNQSNRTMTSGSQATNLGGSQLQTSGLGLFGTTSNQSNTSSGL<br>SGQMGKMNTAGTQTFGSNSNASSAGSQTTQPSSAGNNNQTSTFGGGNPLATNQKMPTSQQSPQFTNGQVMGLFGQNTG<br>QGTNTTSLAATNVLNKSFQAPQFGTNKLNGSSNSNSNLNNNASGTTGTPLGLFGNGSAGQQSPNSSATGGPLNLSNSP<br>ASFGNGSATAATNGNNGMMPMGKTGSTSTNGLFGNSNGIGQNSTFNNSQQTPNGNTNVSTFGTNPSTNLKSSIGKDFT<br>LAQVNKTTNNASIIEAIPYYNLVKKSSNKRRSYNEDDPRLGENASSAKAKKFQLDREQLNSAGKDDFDIQLLSPHESV<br>FMIMLNAGNLDARLDANNKVESLNSNDAQRSNGENPKPRAMTPLTISSEKDSISSSMKIDPAKHLTEPSQDIEQKKLT<br>TTWKSSDEKPVSVPKSTNSSEEC**
0.0239<br>(0.5x)	52<br>(4x)	GPSSGPSMSSGGTNQNHTMSQMGNNNFQSSITNLSNTQPAKSQLAAALGQPMMSFGQMPTSQPMMQGANMTFAVNSGS<br>TLGNTHGTMMQQQAQLGNKLSMQMPGTGMIGLFGGGLGAMKTMGSMVQQTSSTPASTPMTQTNGIMQSQPNNTNLSMN<br>SALGTAVSFGAVMSYAKALMPISSGNPQTHAQNVLSTQNMANMATQMNMGLLSNANGNNKANGLFGQPAMFSQAGSQT<br>FAPVSQLMMAPANGAAGAPMVTQTAQASPPAMHQAMPMNTFGGVPQTQKSAAMMHMQSSANGMSGNMMQNGMSGTQHN<br>PINMGLLSAPSNKGGTGLFGIVSTMLGAPSQMSNQNMPSNRPMTQGSMATNQMTQQLQTAGLLNTQTPMQQSFGQMGL<br>HGQMMAMSTAGTMTNAMNSQASLSPSAQTQPSSAGPNMLVSASAGPNPFGTHQSNMTSQQSPQFTSGQVLPLNLQMPG<br>MGTNQQPMMQQNVLNKSFQAPQHIGLFGNGSSNSQSALMLLGSGTQGTPLGMNSGLAAGQPQANSAATGGQMLSMMMA<br>APFGNQSAPVSTNGGSAMMPMGHTVMTSTNNNAMNSQTITNQPTFNNSQQTPPGNPGLFGQNTMPHTNLKSSIGKDFT<br>LAQVNKTTNNASIIEAIPYYNLVKKSSNKRRSYNEDDPRLGENASSAKAKKFQLDREQLNSAGKDDFDIQLLSPHESV<br>FMIMLNAGNLDARLDANNKVESLNSNDAQRSNGENPKPRAMTPLTISSEKDSISSSMKIDPAKHLTEPSQDIEQKKLT<br>TTWKSSDEKPVSVPKSTNSSEEC
**0.0478<br>(1x)**	**52<br>(4x)**	**GPSSGPSANSGGTNQNTTASTSGNNNFSGSITNLSNTQPAKNQLSTNLGQPAGSFGKMNTSQPMMQGQNGTFPVNSGS<br>NLGNNNGRGQNQQAQLGNKLSPQGTGTGSIGLFGGGLGGSKSQGSQVQQTSSTQASNPNTQTNGIMQSQNNNTNLSTN<br>SQLGTAVSFGAVASYAKGLMPISSGNTQTGAQNVLSTQNAANSNTSTNGGLLSNANGNNKANGLFGQSAAFSQAGSQT<br>FPNVSQLAPNTANGPQGAPMVTQGNQTSSPSNSSAMPTNTFGGVPQTQKSAAMQSANSSANGENGNMMQNGTSGTKGN<br>PINAGLLSQPSNKGGKGLFGIVSTALGAGSQNSNSNMPSNRTMTSGSQATNQTTSQLQTSGLLNTQTTMQQSFGSSGL<br>SGQMGAASTAGTQTNQSNSNASLSGSQTTQPSSAGNNSLVSTSAGGNPFGTNQKNNTSQQSPQFTNGQVLPLNLQNTG<br>QGTNTTPMMQTNVLNKSFQAPQNIGLFGNGSSNSNSNLGLLGSGTTGTPLGQNSNLAAGQQSPNSSATGGQMLSSNSP<br>ASFGNGSATVSTNGNNGMMPMGKTVTTSTNNNNANSNGITNQPTFNNSQQTPNGNTGLFGQNTNPSTNLKSSIGKDFT<br>LAQVNKTTNNASIIEAIPYYNLVKKSSNKRRSYNEDDPRLGENASSAKAKKFQLDREQLNSAGKDDFDIQLLSPHESV<br>FMIMLNAGNLDARLDANNKVESLNSNDAQRSNGENPKPRAMTPLTISSEKDSISSSMKIDPAKHLTEPSQDIEQKKLT<br>TTWKSSDEKPVSVPKSTNSSEEC**
0.1912<br>(4x)	52<br>(4x)	GPSSAQSANSGGTNQNTTASTSGNNNSSGSISNGSNTGPAKDQLTTNLGQPAGSFGKMNTSQPMMQGQNGTFPVNSGS<br>NLGERNGRGQDEQAQLGNKLSPQGTGTGSIGLFGGGAGGSKSQESQVQQTSSTGASDPNTQTNGIMQSQNNNTNLSTN<br>SSLGTHVSFGPVASYAKGLMPIQSGNTQTGEQNTLRTQNAANSNTETNGGLLSNANGNKKARGLFGQSGAFSQAGDQT<br>FPNVSQLAPNTANGPRGEPDVTQGNQTSSPSNSSKMPGNTFGGVPQTTKGTAMQSANSSARGENGNMMQNETSGSKPN<br>HINAGLLSQGSNKGKKGLFGPVSTALGAGSHKSNSNMKSNRTMTSPSPSQATNQTTSHLQTGGLLKTQTTMQQSFGSSGL

		GGQMGASSTAGTQTNESNSNASLSGSHTTQPSSMGENSLVSTSAGGNPFGTNQKNRTSQQGPQFTNGQVLPGQLSRTG<br>QGTNTTPPMQTNSLNKSPQAPQKIGLFGNGSSNSNSNLGLLRSGSTKTPLGHKSNLAAGQESPGQSATGEQMLESNSP<br>ESFGNGSATVSTNGDNGMMPMDKTVKRSTNNENANSRRPGNQPTFNNSQKTPNGNTGLFGQNTNPSTELKSSIGKDFT<br>LAQVNKTTNNASIIEAIPYYNLVKKSSNKRRSYNEDDPRLGENASSAKAKKFQLDREQLNSAGKDDFDIQLLSPHESV<br>FMIMLNAGNLDARLDANNKVESLNSNDAQRSNGENPKPRAMTPLTISSEKDSISSSMKIDPAKHLTEPSQDIEQKKLT<br>TTWKS
0.3824<br>(8x)	52<br>(4x)	GPGEKPSANDGGTNQNTTQSTGENDNFSGKQTNQSNSTPAKNQAETELGQPTGSFGKMNTSQPMTQGQNGKFPVNSGS<br>RLGDNNGRGRNDQAQLGNKLSPTGTGEGSTGLFGKDLGESKSQRSQVQETSSSQASNPETTTEGIMDGQNNNTRKGKN<br>STKETAVSFGAVASAAKKLMPISSGNDQTRAQNVDGGQMAANSNEDGNGGSLSNANGNNKQNGLFGQREAFSQAERHT<br>FPNRSQAAPNTANGPHGEPMMTQGNQTSSPSNSSAMPTNKFGGSQQTQKSAAHSSANSGKNGENGNMMQDGTSGTKGR<br>PGEGGLSDQPSNKGDKGLFGIVGTAKGAGSQNKNSNMPSNRTDTSGSQATNQTGSQTQTSGDLNTQETMDQGFGKSKP<br>SGQMGKAKTADTDTRQSNRNRSLSGSQTRQPSSPGNNSLASTDAGGNPFGKNQKNNTRQRSPQFSNGEKLPLETQNTR<br>QGTNTTEMMKTRVANKSTQAPQNTGLFGNGSGNSNSNMGLLGSGTTDTPLGQKDNTSAGQQSPNSEAGKGQMLSSESP<br>AKFGRGSATPEGKKNEGMMGMGKSVTTSTNNNDANSNKITNGPSSRNSRQTENGRTGLFGGNTNPSTELKSSIGKDFT<br>LAQVNKTTNNASIIEAIPYYNLVKKSSNKRRSYNEDDPRLGENASSAKAKKFQLDREQLNSAGKDDFDIQLLSPHESV<br>FMIMLNAGNLDARLDANNKVESLNSNDAQRSNGENPKPRAMTPLTISSEKDSISSSMKIDPAKHLTEPSQDIEQKKLT<br>TTWKSSDEKPVSVPKSTNSSEEC
0.5736<br>(12x)	52<br>(4x)	GPSSGPSANSEETEQRSTRDTKEDDNMSGSMGELDKTGPRKNTLSTNLEQPAGSFGKMNTGQPMMKKRNGTFPVNDGE<br>ELDNNRRRGQEKGHSLKEKLGPQKTRGGSIGLFGDRSGESKRQRSSVQQTSSTRASNSETKTEGIMQSQKNNRNLSRN<br>DQTETHEKFGAVASYAKGLGPRSGEETSGKASNMLSSTNGANENTSKKGKLLEDHDGNNKARGLFGESSTMSQSKSST<br>FQNTSQEAPKTGNGKQRAPMGGQGNETGSPEEESKPPSNTFGGVPQTQKGATMQSSNDSAKGENGNAMQNGTKEEKKK<br>PINAKRLSQPSEKGGKGLFGPVSTALGAGESKEESNMDKNRRATSGSQAGEQTTSQLQTSRLPDTTSDMQQGFGSSGL<br>SGGMDKPSTAGTQTNQSNSNARSSKSQTTQTSSAGNESLVSTGAKERPFGGNQKNDDSQEEDQFTNGQVLPTNMRETG<br>QGTNKTPRQQENQLNKSKQRPQNSGLFGNRSSKGNKNLKALGSGETRTHSGQNSETATRQESPEGGATDGSHLSKNGP<br>AEFGNGKATGGSNENRGMMGMDKGVTTGKNNNNAKSKGQTNQKTGNNGTSTTNGETGLFGGNEEPESNLKSSIGKDFT<br>LAQVNKTTNNASIIEAIPYYNLVKKSSNKRRSYNEDDPRLGENASSAKAKKFQLDREQLNSAGKDDFDIQLLSPHESV<br>FMIMLNAGNLDARLDANNKVESLNSNDAQRSNGENPKPRAMTPLTISSEKDSISSSMKIDPAKHLTEPSQDIEQKKLT<br>TTWKSSDEKPVSVPKSTNSSEEC
0.0239<br>(0.5x)	104<br>(8x)	GPQSAPSANSMGTNQLMMASTSAQMGFTAQAAISHNTQAMPPMLSPNLGMPAGSNQHMNPSMNMLLGQHGPFPVMSSS<br>SAMHNNQRTQNQQMQLPMMASPMMTGHGFGITNISGLGGSPSQGSQVQVSATPQASHMNMQTNAALSPQGNNTNLSTA<br>TQMPTAVMMSMVMSYASQMMQISSGNTQTMAQGVLTQQNMANSNTSTSMQMMPNMNGLFGANTNQPMPAAFSAAGSMP<br>FPNSIPLAPNTANGPQQAPMLTQGNPTSSPANQSAMPTPTLAGVAMTQGTPMLGSANTMANGENGTMIPNGTSMTQGN<br>PINAMVRQFGSSKGGKATTSILSTALTAGSPATHSNLPMNRPMTSMSQAMNSMMPQLPTMPLLQTQTTQPQMPTSSGL<br>QGQMHPVMQAGAMAGQSNSNAALPGSMTAQPSSAGNGLFGTTSAGQNPLATNMKMAMSAQSPQFMMGAVMPLNLPNTG<br>QGHNTTPMMQTNVLPPPFQAPKNISNTLQGTHPSNSNLTLISTPTTGHPLGANMNMQMGQASPNSSFGGGGLAASHSP<br>MPPTNPSATLSQHGNNTMMPMPAAMQTSTNAAPMNSNSIQNAKPFNNSAPPMNGNTISSQQQTMMPPNLKSSIGKDFT<br>LAQVNKTTNNASIIEAIPYYNLVKKSSNKRRSYNEDDPRLGENASSAKAKKFQLDREQLNSAGKDDFDIQLLSPHESV<br>FMIMLNAGNLDARLDANNKVESLNSNDAQRSNGENPKPRAMTPLTISSEKDSISSSMKIDPAKHLTEPSQDIEQKKLT<br>TTWKSSDEKPVSVPKSTNSSEEC
**0.0478<br>(1x)**	**104<br>(8x)**	**GPSSGPSANSGGTNQLSTASTSGNNNFSGSAAISSNTQPAKNQLSTNLGQPAGSNNKMNTSQNSLLGQNGTFPVNSGS<br>NATNNNGRGQNQQAQLPMAASPQGTGTGFGITNISGLGGSKSQGSQVQVSSSTQASNPNTQTNAALSSQNNNTNLSTN<br>SQMPTAVSQSAVASYAKQMMQISSGNTQTGAQNVLSTQNAANSNTSTNGQMMPNANGLFGANTNQPMPAAFSQAGSQT<br>FPNSIQLAPNTANGPQGAPMLSQGNQTSSPSNSSAMPTNTLPGVPQTQKSPMLGSANSSANGENGNMISNGTSGTKGN<br>PINATVMQFGSNKGGKQTTSILSTALGAGSQNSNSNLGSNRTMTSGSQATNAAMPQLQTSGLLNTQTTQPQSNTSSGL<br>SGQMGTVMQAGTQTNQSNSNASLSGSQTTQPSSAGNGLFGGTSAGGNPLATNQKMQTSQQSPQFTNGQVMPLNLQNTG<br>QGTNTTPMMQTNVLNKSFQAPQNISNKLNGSSNSNSNLGLISSGTTGTPLGQNSNMQAGQQSPNSSFGGGGLAASNSP<br>ASQTNGSATLSTNGNNGMMPMGKTMQTSTNNNNANSNGIGNNKTFNNSQQTPNGNTISSTQNTNPSTNLKSSIGKDFT<br>LAQVNKTTNNASIIEAIPYYNLVKKSSNKRRSYNEDDPRLGENASSAKAKKFQLDREQLNSAGKDDFDIQLLSPHESV<br>FMIMLNAGNLDARLDANNKVESLNSNDAQRSNGENPKPRAMTPLTISSEKDSISSSMKIDPAKHLTEPSQDIEQKKLT<br>TTWKSSDEKPVSVPKSTNSSEEC**
0.1912<br>(4x)	104<br>(8x)	GPSSGPGADSRGTNQLSTASTSGENNFSGSAAISSNTKPAKNQLSGNLGQPAGSNNKMNTSQNSLLGQNGSFPVNSGS<br>NATNNNGRGQNQQAQLPMAASPQGTMTDFGPTNISGLGGSKDQGSTVQVSSSTQAKNPNTQTNPQLQSQNNNTELSTK<br>SQHPTAVSQSAVASAAKGMMQISSGNTSTGAQNVLSTQEAAESNTSRNGQMQPNARGLFGANTNQPMQTDFSQSGSQS<br>SPNSIELAPNTSKRPQGAPMQSQGNQTSSPSNSSSMPTNTLPGQGQTQKSPMLGSPNSSGDGENGNMISNGTEGTKGN<br>PINASVMQFGSNKGGKQTTSHLSTPLGAGSQRHNSNLGRNRTMTSGSQATNAAMPQLQDSGLLNTQSTQPQSNSESKL<br>SDQMGTVMQAGTQKNQSNSNMSLSGSQTTQPSSAGNGLFGGTKARGKPLATNSKMMTSHQSPQFSNGQMMPLNGQNTG<br>QGDETTPMMQKNVMNKSFQASQNKSNKLNGSSNHNSNLGLISSGTTGTPLGHNSNMQAEQQSPNSSFGGEDLDASNSP<br>AGQTNGSAELSTNGNPGMDSMGKTMQTSRNNNKAKSNGIGNRKTFNNGQQTPNGNTISSSQNTRPSTNLKSSIGKDFT<br>LAQVNKTTNNASIIEAIPYYNLVKKSSNKRRSYNEDDPRLGENASSAKAKKFQLDREQLNSAGKDDFDIQLLSPHESV<br>FMIMLNAGNLDARLDANNKVESLNSNDAQRSNGENPKPRAMTPLTISSEKDSISSSMKIDPAKHLTEPSQDIEQKKLT<br>TTWKSSDEKPVSVPKSTNSSEEC
0.3824<br>(8x)	104<br>(8x)	GPSRGPSANGKGTNQGPTASTSENGNFSGSKQISENEQSAKEQLSTNMGQGGGDNNKMDTGQNSHMGQNRGSQVNSTS<br>SAGNNRGRGQNQGAELPMAPSTQGTGTPFGDTNISGHREHKRQGSQVTGSSSTTASNPRTQDNAALKSGNRKTNSETN<br>GQMPTQGSQSEVPSAAKQMMQIGSGNTQTGATRVLSSQRAAEENTSGNGQMAPNSRGLFGANSNQPMKAAFRRAGSGT<br>FPNGSQLATNTMNGPQGAPMLSQENQGSKRSNSSKMPDRTLPKVPQDQKSPMTGQPNSSANGEDGESESNGTSGGKGS<br>PMRARMHQFGKRKGGKQTTSQGSTASGAGKDESEGNLGKERTTTSGETQTEAAMDQLQTSGLLNTHTKHPQSNTDKGL<br>SEHMGSDGSAGTQGNQSNSKASLSGSQTKKPSRRENGLFGGDSAGGKPGATNQKMQTDQGSPSTTNRQVPDLRPGNTR<br>QGSNTRPSMTPEMLNKSFQAPQNISNKLNGGSKSNSNLTQIESGTEGTPLEQNSEMQMGQQGENSSFGKGGQRGSRSP<br>ESSTNGSATPMDNGNNGMMPMKKTGQTSTNNENANSNGIGKNKTFNNSQQTPDGDSTSGTGETNPSTNLKSSIGKDFT<br>LAQVNKTTNNASIIEAIPYYNLVKKSSNKRRSYNEDDPRLGENASSAKAKKFQLDREQLNSAGKDDFDIQLLSPHESV<br>FMIMLNAGNLDARLDANNKVESLNSNDAQRSNGENPKPRAMTPLTISSEKDSISSSMKIDPAKHLTEPSQDIEQKKLT<br>TTWKSSDEKPVSVPKSTNSSEEC
**0.5736<br>(12x)**	**104<br>(8x)**	**GPSKESGADSGGTKQLSTAEDSEKDKFSDSSGHSSETQPAKDEMGTNPKQPHESNNKPNTSQNSLTKQNGTMPVNSGG<br>RDTNNNGRKQKHEAQSGMAPSPQGTGSKFGIENESRLGGSKRHDSHVQTSSSEQASEPNSQTNDTLGSQNENGELSTN<br>STMSSRDEQSHHASYAKQMMQISRGRTQTKAQNPLSTQDAPNSETSGEGQKGPKANGLFGARGDQPMPAAMSQQGSQT<br>KPNSIHLAPNSARKPRGAPMLRHKNKTSKPSESSAMPDNTLPGVPSGHKSQQLGSANSSGNGENDEMTENGTGGTKDN**

		**TIEAKPMQFGREKGKKSTTKGLDTALGADSQNKNSNMGSRRTATSKSGATNHRMPQLQTSKLARTSTTGTQSNTSSGL DRQTGTVMQEGSQSNESRSDASASRSRKSQPSSQENGLFGGRKAGGKPEDTNSKMTTERRSPQFTNEQVSPQRLEETG QETKTESHMEGNTLNKDEQAPQNHSNKLDGSSRRKSRAESISSGTTGTELGQNKNSHHDQQKPNDSFGRGGMAAERSH QSQTRGSATASKKGEERMQPMGKTHQTSTNNDNANSNRKEEEKTPNNKDKRPNGRTAESTQNGDPSTNLKSSIGKDFT LAQVNKTTNNASIIEAIPYYNLVKKSSNKRRSYNEDDPRLGENASSAKAKKFQLDREQLNSAGKDDFDIQLLSPHESV FMIMLNAGNLDARLDANNKVESLNSNDAQRSNGENPKPRAMTPLTISSEKDSISSSMKIDPAKHLTEPSQDIEQKKLT TTWKSSDEKPVSVPKSTNSSEEC**

Supplementary Table 1 – Amino acid sequences of artificial FG-Nups used in simulations and experiments.
The final amino acid sequences include a Cys-residue at the C-terminus, and a Gly-Pro-motif that remains after cleaving off the Protease tag at the N-terminus, putting the total sequence length at 803 residues. Sequences optimized for expression and purified for use in the experiments in this work are highlighted using bold text and color-coded to indicate which proteins were studied experimentally. Blue sequences indicate that the purification was successful, orange sequences indicate that the protein was not studied (either due to incompatible properties or due to unsuccessful purification).

**FG-Nup**	**Residues**	**C/H**	**Average FG/GLFG-motif spacing**
Nup159	387–1071	0.51	24.42
Nup42	1–382	0.071	11.17
Nup116-CD	172–764	0.042	12.61
Nup116-ED	765–960	0.83	196*
Nup100-CD	1–610	0.042	11.1
Nup100-ED	611–800	0.56	190*
Nsp1-CD	1–186	0.041	11.4
Nsp1-ED	187–617	0.748	18.6
Nup2	120-600	0.600	34.9
Nup49	1–251	0.051	11.2
Nup57	1–255	0.05	12.81
Nup145N-CD	1–242	0.06	17.17
Nup145N-ED	243–433	0.49	191*
Nup1-ED	220–797	0.574	46.33
Nup1-CD	798–1076	0.072	38.14
Nup60	389–539	0.539	151*
hsNup98[1]	1-500	0.041	10.35
hsNup153[2]	875-1475	0.2537	22
hsNup214[3]	1632-2090	0.089	10.7

Supplementary Table 2 - Overview of FG-domains of *saccharomyces cerevisiae* and selected domains of *homo sapiens*.
Domain definitions (*e.g.* collapsed or extended) follow those outlined by Yamada et al[4] for yeast FG-Nups and selected human FG-Nups. The FG-motif spacing (in number of residues) is calculated by determining the average amino acid spacing between any FG or GLFG-motifs. The charged-to-hydrophobic amino acid ratio (C/H) was calculated using Equation 1 and the normalized hydrophobicity values in Supplementary Table 4. The domains marked with an asterisk (*) comprise a high charge content and do not contain any ‘FG’ or ‘GLFG’ motifs, hence the spacing coincides with the full length of the domain.

System	System size (no. of beads)	Number of simulations	Forcefield	Timestep (ps)	Simulation time (steps)
Single FG-spacing or C/H ratio variants	803	1400 (50 per d_{FG},C/H)	1-BPA	0.02	1.5 µs ($7.5\cdot10^{7}$)
FG-Nup-coated nanopores in absence of cargo	102731	21	1-BPA-CP[5]	0.02	5 µs ($2.5\cdot10^{8}$)
FG-Nup-coated nanopores in absence of cargo	102731	21	1-BPA-CP[5]	0.0175	5.0 µs ($2.86\cdot10^{8}$)
FG-Nup-coated nanopores in presence of cargo, equilibration	142841	18	1-BPA-CP[5]	0.02	500ns ($2.5\cdot10^{7}$)
FG-Nup coated nanopores in presence of cargo	142841	18	1-BPA-CP[5]	0.02/0.0175	5.0-12µs (up to $6\cdot10^{8}$)
Binding simulations of FG spacing or C/H ratio variants with Kap95	1663	20 per FG-spacing and C/H (420 in total)	1-BPA-CP[5]	0.02	Up to 300 µs ($1.5\cdot10^{10}$)

Supplementary Table 3 – Overview of simulations in this work.

**AA**	A	R*	N	D*	C	Q	E*	G	H	I
	0.7	0.005	0.33	0.005	0.68	0.64	0.005	0.41	0.53	0.98
**AA**	L	K*	M	F	P	S	T	W	Y	V
	1	0.005	0.78	1	0.65	0.45	0.51	0.96	0.82	0.94

Supplementary Table 4 – Hydrophobicity scale used in our 1-BPA model. Hydrophobicity-values of charged residues have been increased with respect to the original version[6]. This hydrophobicity scale is used to calculate the C/H-ratio values.

Particle type	Consists of:	(nm)
AA	All residues not involved in cation-pi (RK/FYW) or binding site (B-RK, B-FYW, F1, G1) interactions	0.6
RK	R or K-residues that are not part of a Kap95 binding site	0.6
FYW	F, Y or W residues that are not part of a Kap95 binding site or FG-motif (in case of F).	0.6 (hydrophobic) or 0.45 (cation-pi)
B-RK	R or K-residues that are part of a Kap95 binding site	0.6 or 0.45
B-FYW	F, Y or W residues that are part of a Kap95 binding site	0.6 or 0.45
F1	F-residue within an FG-motif	0.6
G1	G-residue within an FG-motif	0.6
WALL	Sterically beads that constitute the nanopore scaffold	3
BOUNDS	Sterically inert beads that confine only Kap95 to a cylindrical region on either side of the nanopore membrane	3

Supplementary Table 5 – Overview of interaction types.

	62.5 nM Kap95					500 nM Kap95				
NupY variant - rep #	Δf_{max} (Hz)	$t_{1/2}$ (min)	τ_1 (min)	τ_2 (min)	(t_1,t_2) min	Δf_{max} (Hz)	$t_{1/2}$ (min)	τ_1 (min)	τ_2 (min)	(t_1,t_2) min
NupY(13;0.05) - rep1	-16.074	10.016	0.044	0.53	(3.0, 19.0)	-42.945	26.852	0.021	0.412	(26.5, 44.5)
NupY(13;0.05) - rep2	-20.175	20.748	0.027	0.418	(2.5, 22.0)	-39.29	14.567	0.047	0.576	(30.0, 45.5)
NupY(13;0.05) - rep3	-24.822	6.639	0.082	0.64	(4.5, 25.0)	-51.581	16.954	0.429	0.032	(27.5,45.0)
NupY(52;0.05) - rep1	-5.277	9.867	0.047	0.408	(3.0, 22.0)	-12.779	8.634	2.591	0.07	(25.5, 41.0)
NupY(52;0.05) - rep2	-7.694	4.389	0.081	0.644	(3.3, 21.0)	-10.729	15.877	0.917	0.037	(27.0, 42.5)
NupY(104;0.05) - rep1	-6.608	1.911	1.072	0.109	(3.0, 22.0)	-12.424	0.703	1.112	0	(26.0, 30.0)
NupY(104;0.05) - rep2	-1.8	1.982	1.374	0.136	(2.4, 22.0)	-9.954	4.089	0.181	0	(35.0, 48.0)
NupY(104;0.57) - rep1	-8.441	7.312	1.024	0.073	(3.0, 23.0)	-19.416	6.43	0.079	1.102	(26.0, 44.0)
NupY(13;0.09) - rep1	-8.919	7.554	0.853	0.085	(6.0, 23.0)	-31.17	15.962	0.038	2.065	(26.3, 42.0)
NupY(13;0.09) - rep2	-12.017	14.315	0.044	0.572	(3.5, 25.0)	-49.14	13.109	0	0.053	(32.0, 44.5)
NupY(13;0.38) - rep1	-16.269	7.873	0.674	0.042	(4.0, 23.0)	-30.257	10.222	0.053	3.778	(25.0, 42.2)
NupY(13;0.38) - rep2	-19.243	7.58	0.056	0.345	(3.5, 25.0)	-31.74	10.884	0.056	0.924	(27.5, 47.0)
NupY(13;0.38) - rep3	-5.835	7.43	0.074	0.912	(4.0, 24.0)	-11.851	10.239	0.051	1.051	(25.0, 44.0)

Supplementary Table 6 – Extracted parameters from fitting frequency shifts upon Kap95 binding to NupY brushes.

The parameters Δf_{max}, $t_{1/2}$, were extracted from fitting the function to the frequency shifts obtained from Kap95 binding curves at 62.5 nM and 500 nM across the different NupY variants and replicates. The time interval (t_1, t_2) corresponds to the region where the fit was performed, identified upon analyzing the $\Delta D/\Delta f$ response (Methods and Supplementary Figure 10c-e). Examples of fits are reported in Figures 2h-i and Supplementary Figure 10a-b.

Protein	Sequence
**Kap95**	MSTAEFAQLLENSILSPDQNIRLTSETQLKKLSNDNFLQFAGLSSQVLIDENTKLEGRILAALTLKNEL VSKDSVKTQQFAQRWITQVSPEAKNQIKTNALTALVSIEPRIANAAAQLIAAIADIELPHGAWPELMK IMVDNTGAEQPENVKRASLLALGYMCESADPQSQALVSSSNNILIAIVQGAQSTETSKAVRLAALNA LADSLIFIKNNMEREGERNYLMQVVCEATQAEDIEVQAAAFGCLCKIMSLYYTFMKPYMEQALYAL TIATMKSPNDKVASMTVEFWSTICEEEIDIAYELAQFPQSPLQSYNFALSSIKDVVPNLLNLLTRQNED PEDDDWNVSMSAGACLQLFAQNCGNHILEPVLEFVEQNITADNWRNREAAVMAFGSIMDGPDKVQ RTYYVHQALPSILNLMNDQSLQVKETTAWCIGRIADSVAESIDPQQHLPGVVQACLIGLQDHPKVAT NCSWTIINLVEQLAEATPSPIYNFYPALVDGLIGAANRIDNEFNARASAFSALTTMVEYATDTVAETS ASISTFVMDKLGQTMSVDENQLTLEDAQSLQELQSNILTVLAAVIRKSPSSVEPVADMLMGLFFRLLE KKDSAFIEDDVFYAISALAASLGKGFEKYLETFSPYLLKALNQVDSPVSITAVGFIADISNSLEEDFRR YSDAMMNVLAQMISNPNARRELKPAVLSVFGDIASNIGADFIPYLNDIMALCVAAQNTKPENGTLEA LDYQIKVLEAVLDAYVGIVAGLHDKPEALFPYVGTIFQFIAQVAEDPQLYSEDATSRAAVGLIGDIAA MFPDGSIKQFYGQDWVIDYIKRTRSGQLFSQATKDTARWAREQQKRQLSLLPETGG
**IBB-GFP**	MSKHHHHSGHHHTGHHHHSGSHHHTGENLYFQGSDNGTDSSTSKFVPEYRRTNFKNKGRFSADELR RRRDTQQVELRKAKRDEALAKRRNFIPPTDGTSKGEELFTGVVPILVELDGDVNGHKFSVSGEGEGD ATYGKLTLKFICTTGKLPVPWPTLVTTLTYGVQCFSRYPDHMKQHDFFKSAMPEGYVQERTIFFKDD GNYKTRAEVKFEGDTLVNRIELKGIDFKEDGNILGHKLEYNYNSHNVYIMADKQKNGIKVNFKIRHN IEDGSVQLADHYQQNTPIGDGPVLLPDNHYLSTQSALSKDPNEKRDHMVLKEFVTAAGITLGMDEL YKTGC
**2xMBP-IBB**	MKIEEGKLVIWINGDKGYNGLAEVGKKFEKDTGIKVTVEHPDKLEEKFPQVAATGDGPDIIFWAHDR FGGYAQSGLLAEITPDKAFQDKLYPFTWDAVRYNGKLIAYPIAVEALSLIYNKDLLPNPPKTWEEIPA LDKELKAKGKSALMFNLQEPYFTWPLIAADGGYAFKYENGKYDIKDVGVDNAGAKAGLTFLVDLI KNKHMNADTDYSIAEAAFNKGETAMTINGPWAWSNIDTSKVNYGVTVLPTFKGQPSKPFVGVLSAG INAASPNKELAKEFLENYLLTDEGLEAVNKDKPLGAVALKSYEEELVKDPRIAATMENAQKGEIMPN IPQMSAFWYAVRTAVINAASGRQTVDEALKDAQTNSSSNNNNNNNNNNLGIEGRISHMLEVLFQGP MKIEEGKLVIWINGDKGYNGLAEVGKKFEKDTGIKVTVEHPDKLEEKFPQVAATGDGPDIIFWAHDR FGGYAQSGLLAEITPDKAFQDKLYPFTWDAVRYNGKLIAYPIAVEALSLIYNKDLLPNPPKTWEEIPA LDKELKAKGKSALMFNLQEPYFTWPLIAADGGYAFKYENGKYDIKDVGVDNAGAKAGLTFLVDLI KNKHMNADTDYSIAEAAFNKGETAMTINGPWAWSNIDTSKVNYGVTVLPTFKGQPSKPFVGVLSAG INAASPNKELAKEFLENYLLTDEGLEAVNKDKPLGAVALKSYEEELVKDPRIAATMENAQKGEIMPN IPQMSAFWYAVRTAVINAASGRQTVDEALKDAQTCCGPCCDNGTDSSTSKFVPEYRRTNFKNKGRF SADELRRRRDTQQVELRKAKRDEALAKRRNFIPPTDHHHHHHHH

Supplementary Table 7 – Amino acid sequences of Kap95 and cargoes used in the study.

Variant	FCR	NCPR	kappa	pI (full protein)	neg	pos				
7-0.05	0,026	0,020	0,261	9,63	2	14	349	0,032	0	0,000
7-0.19	0,103	0,021	0,177	9,63	25	38	55	0,073	43	0,070
7-0.38	0,186	0,020	0,214	9,19	51	63	47	0,106	43	0,163
7-0.57	0,258	0,020	0,175	9,19	73	85	39	0,205	29	0,138
13-0.02	0,015	0,015	0,214	9,63	0	9	537	0,017	0	0,000
13-0.05	0,026	0,023	0,207	9,73	1	15	302	0,026	0	0,000
13-0.09	0,051	0,021	0,206	9,63	9	22	112	0,027	0	0,000
13-0.19	0,098	0,023	0,181	9,63	23	37	58	0,138	57	0,053
13-0.38	0,178	0,021	0,191	9,41	48	61	60	0,100	51	0,157
13-0.57	0,250	0,021	0,202	9,19	70	83	48	0,125	33	0,152
26-0.05	0,026	0,023	0,207	9,73	1	15	302	0,030	0	0,000
52-0.02	0,015	0,015	0,214	9,63	0	9	571	0,016	0	0,000
52-0.05	0,026	0,023	0,207	9,73	1	15	302	0,023	0	0,000
52-0.19	0,096	0,024	0,207	9,73	22	37	96	0,083	51	0,039
52-0.38	0,175	0,024	0,170	9,40	46	61	37	0,081	32	0,125
52-0.57	0,246	0,021	0,195	9,19	69	82	32	0,156	20	0,150
104-0.02	0,015	0,011	0,270	9,41	1	8	293	0,024	0	0,000

104-0.05	0,026	0,023	0,207	9,73	1	15	302	0,030	0	0,000
104-0.19	0,098	0,023	0,228	9,63	23	37	68	0,059	42	0,143
104-0.38	0,176	0,023	0,204	9,41	47	61	46	0,087	27	0,185
104-0.57	0,246	0,021	0,167	9,19	69	82	46	0,152	40	0,150

Supplementary Table 8 – Sequence charge properties of NupY-mutants.
Blue shading indicates that the protein was used experimentally. Fraction of charged residues (FCR), net charge per residue (NCPR), charge patterning (kappa) were calculated using localCIDER[7,8], positive and negative like-charge region lengths and densities were calculated from the work of Peyro *et* al.[9,10] . The isoelectric point (pI) is here expressed as the value for the full domain, all other values are obtained for the systematically varied (1-613) collapsed domain.

**Name**	**FCR**	**NCPR**	**kappa**	**pI**	**PP region**				
Nup49 collapsed	0,028	0,028	0,216	11,81	1	246	0,028	0	0,000
Nup57 collapsed	0,027	0,027	0,216	12,25	1	242	0,029	0	0,000
Nup100 collapsed	0,022	0,018	0,183	11,81	1	376	0,024	0	0,000
Nup116 collapsed	0,024	0,020	0,196	11,16	1	539	0,022	0	0,000
Nup145N collapsed	0,033	0,025	0,209	10,72	1	216	0,032	0	0,000
Nup100 extended	0,263	0,000	0,178	7,00	2	24	0,167	17	0,294
Nup116 extended	0,352	-0,005	0,163	7,00	3	29	0,241	9	0,444
Nup145 extended	0,241	0,010	0,200	9,19	1	42	0,143	21	0,333
NupX collapsed	0,019	0,019	0,372	12,25	1	144	0,021	0	0,000
NupX extended	0,268	0,000	0,155	7,00	2	23	0,130	24	0,292
hsNup98	0.0501	0.014	0.155	9.73	1	261	0.027	27	0.074
hsNup214	0.024	0.011	0.176	10.28	1	72	0.042	0	0
hsNup153	0.135	-0.002	0.094	7	1	24	0.083	14	0.143

Supplementary Table 9 – sequence charge properties of native GLFG-Nups and NupX. Fraction of charged residues (FCR), net charge per residue (NCPR), charge patterning (kappa) were calculated using localCIDER[7,8], positive and negative like-charge region lengths and densities were calculated from the work of Peyro *et* al.[9,10]

test	dependent variable	factor	F	df_between	df_within	P value	η^2
one-way ANOVA	$-\Delta D/\Delta f$ (7th harmonic)	NupY variant	17.991	6	18	1.039e-06	0.857

Supplementary Table 10 – statistical analysis of −ΔD/Δf across NupY variants.

One-way ANOVA results for the dissipation-to-frequency ratio (−ΔD/Δf, 7th harmonic) across NupY variants. The table reports the F statistic, degrees of freedom (df_between, df_within), exact P value, and effect size (η^2). Statistical tests were two-sided.

group 1	group 2	mean difference	adjusted P value	95% CI lower	95% CI upper	significant
NupY(13;0.09)	NupY(13;0.19)	-0.0021	9.987e-01	-0.0163	0.0121	FALSE
NupY(13;0.09)	NupY(13;0.38)	0.0058	7.555e-01	-0.0072	0.0187	FALSE
NupY(13;0.09)	NupY(104;0.05)	0.0189	1.300e-03	0.0066	0.0312	TRUE
NupY(13;0.09)	NupY(104;0.57)	0.0111	3.866e-01	-0.0063	0.0285	FALSE
NupY(13;0.09)	NupY(52;0.05)	0.0053	7.864e-01	-0.007	0.0176	FALSE
NupY(13;0.09)	NupY(13;0.05)	-0.0065	4.902e-01	-0.0175	0.0046	FALSE
NupY(13;0.19)	NupY(13;0.38)	0.0079	4.426e-01	-0.0051	0.0208	FALSE
NupY(13;0.19)	NupY(104;0.05)	0.021	4.000e-04	0.0087	0.0333	TRUE
NupY(13;0.19)	NupY(104;0.57)	0.0132	2.128e-01	-0.0042	0.0306	FALSE
NupY(13;0.19)	NupY(52;0.05)	0.0074	4.588e-01	-0.0049	0.0196	FALSE
NupY(13;0.19)	NupY(13;0.05)	-0.0044	8.428e-01	-0.0155	0.0067	FALSE
NupY(13;0.38)	NupY(104;0.05)	0.0131	1.210e-02	0.0023	0.0239	TRUE
NupY(13;0.38)	NupY(104;0.57)	0.0053	9.277e-01	-0.0111	0.0217	FALSE
NupY(13;0.38)	NupY(52;0.05)	-0.0005	1.000e+00	-0.0113	0.0103	FALSE
NupY(13;0.38)	NupY(13;0.05)	-0.0122	6.800e-03	-0.0217	-0.0028	TRUE
NupY(104;0.05)	NupY(104;0.57)	-0.0078	6.706e-01	-0.0236	0.0081	FALSE
NupY(104;0.05)	NupY(52;0.05)	-0.0136	4.300e-03	-0.0237	-0.0036	TRUE
NupY(104;0.05)	NupY(13;0.05)	-0.0254	<1e-15	-0.0339	-0.0168	TRUE
NupY(104;0.57)	NupY(52;0.05)	-0.0058	8.788e-01	-0.0217	0.01	FALSE
NupY(104;0.57)	NupY(13;0.05)	-0.0176	1.550e-02	-0.0325	-0.0026	TRUE
NupY(52;0.05)	NupY(13;0.05)	-0.0117	3.900e-03	-0.0203	-0.0032	TRUE

Supplementary Table 11 – pairwise statistical comparisons of −ΔD/Δf across NupY variants.

Tukey's honestly significant difference (HSD) test for pairwise comparisons of the dissipation-to-frequency ratio (−ΔD/Δf, 7th harmonic) across NupY variants following one-way ANOVA. The table reports mean differences, adjusted P values (family-wise error rate corrected), and 95% confidence intervals (CI). Statistical tests were two-sided.

Analysis	Parameter	Harmonic	Slope	SE	t statistic	df	P value	95% CI lower	95% CI upper	R^2
C/H	t1/2	7_1	-4.3949	2.7281	-1.611	7	1.512e-01	-10.8458	2.056	0.27
C/H	t1/2	7_2	-4.5081	1.801	-2.503	6	4.634e-02	-8.915	-0.1011	0.511
C/H	|Δfmax|	7_1	5.9105	3.936	1.502	7	1.769e-01	-3.3966	15.2176	0.244
C/H	|Δfmax|	7_2	11.3717	3.7396	3.041	6	2.278e-02	2.2213	20.5221	0.606
FG-spacing	t1/2	7_1	-6.7562	2.7114	-2.492	6	4.705e-02	-13.3909	-0.1216	0.509

FG-spacing	t1/2	7_2	-7.6176	2.2284	-3.418	5	1.887e-02	-13.346	-1.8892	0.7
FG-spacing	|Δfmax|	7_1	11.0021	3.1998	3.438	6	1.383e-02	3.1725	18.8317	0.663
FG-spacing	|Δfmax|	7_2	17.5582	2.8717	6.114	5	1.697e-03	10.1762	24.9402	0.882

Supplementary Table 12 – logarithmic regression analysis of Kap95 binding parameters across NupY variants.

Ordinary least-squares logarithmic regression analysis of extracted parameters (|Δfmax| and t1/2) as a function of FG spacing or C/H-value. The table reports slope, standard error (SE), t statistic, degrees of freedom (df), exact P value, 95% confidence intervals (CI), and coefficient of determination (R^2). Statistical tests were two-sided t-tests of the slope of the fitted logarithmic model. Results are shown for both Kap95 concentrations: 7_1 corresponds to 62.5 nM and 7_2 to 500 nM. Values reported in the main text correspond to the 7_2 (500 nM) measurements.

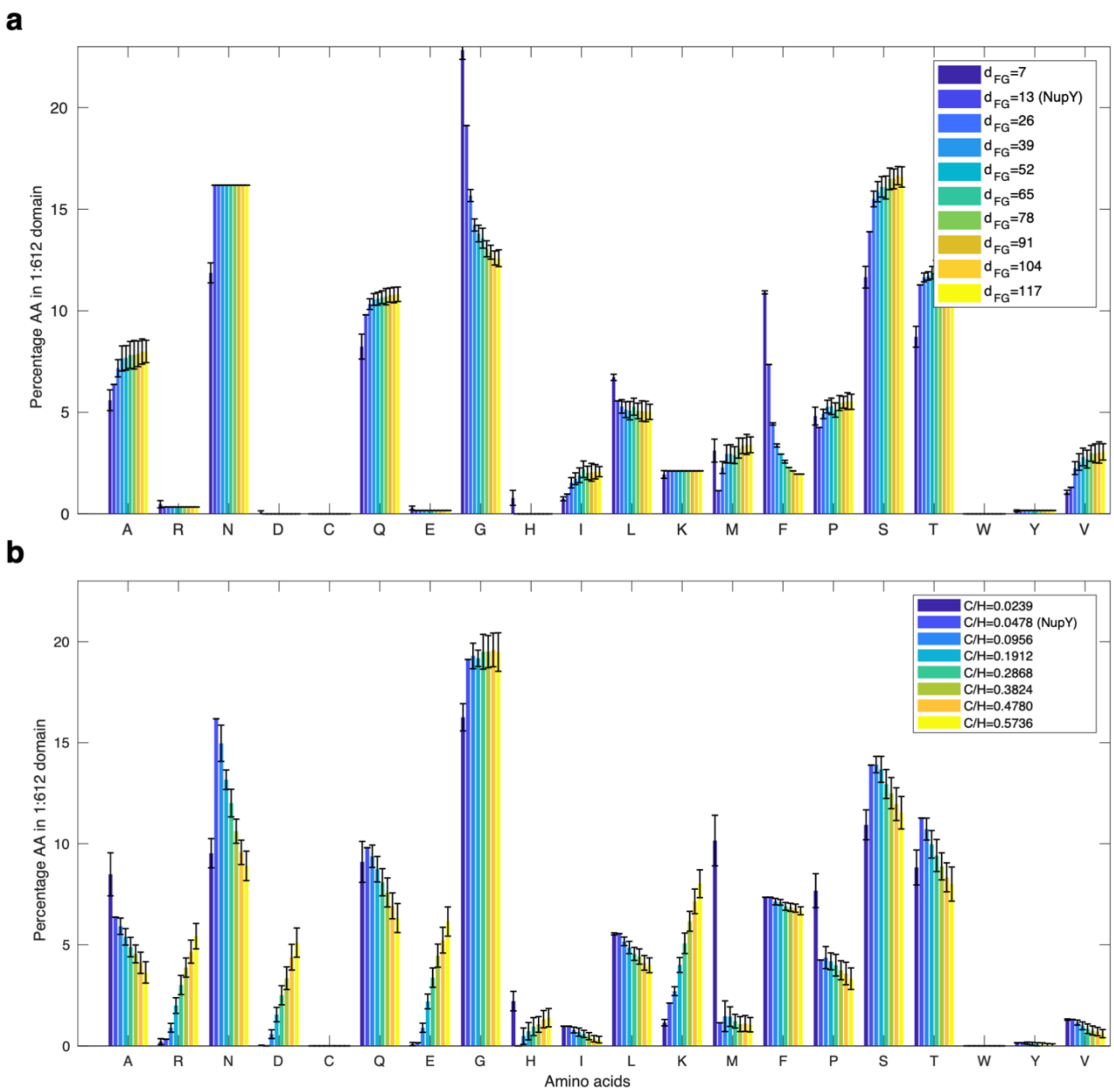

Supplementary Figure 1 – Sequence histograms for FG-spacing variants and C/H-ratio variants. **a:** The average occurrence of different amino acids in the 50 highest-ranking designs for FG-spacing variants (with fixed C/H-ratio at the native GLFG-Nup average of 0.0478). Increasing the FG-spacing leads to an exchange of FG and GLFG-motifs motifs for other isohydrophobic pairs of amino acids (AA, MQ, MP, IG, LF, IS, LS, VS, VT). **b:** The average occurrence of different amino acids in the 50 highest-ranking designs for C/H-ratio variants (with fixed FG-spacing at the native GLFG-Nup average of 13). To achieve higher C/H-ratios, additional charge content (D,E,K,R) is introduced that replaces polar uncharged residues (N,Q,T,S). To reduce the hydrophobicity below the native GLFG-Nup average of 0.0478, additional hydrophobic residues were introduced that do not strongly enhance secondary structure (*e.g.*, A, M, or P). For both panels, error bars indicate the standard deviation, N=50 variants per design value.

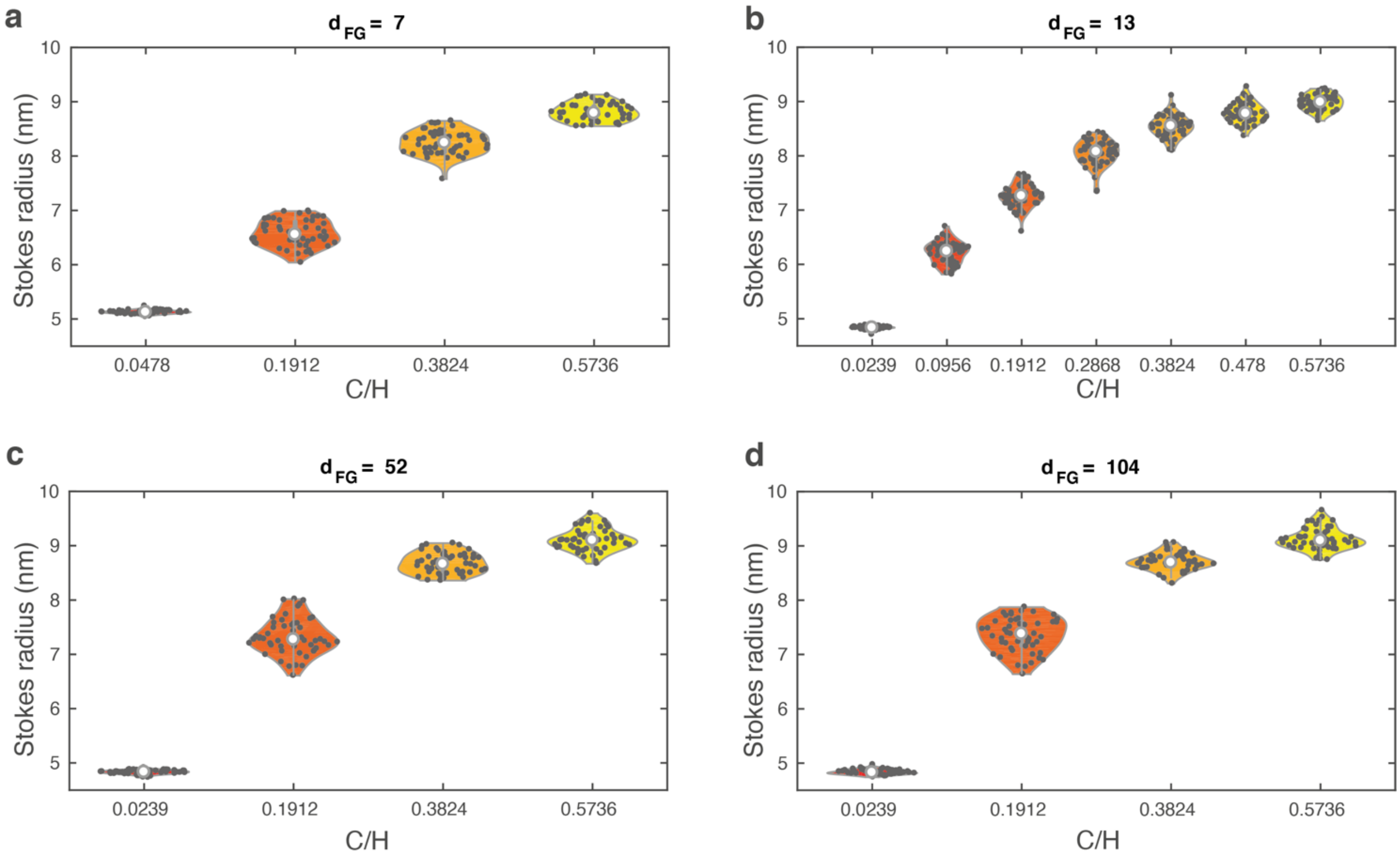

Supplementary Figure 2 – Violin plots of the Stokes radii of 50 different designs for different combinations of C/H-ratio and FG-spacing. Compared to the Stokes radii for designs comprising C/H and d_{FG}-values close to the GLFG-Nup average (*i.e.,* panel (b) is reproduced from Figure 1g in the main text), we observe similar trends for other combinations (a,c,d). Namely, increases in C/H lead to a monotonic increase in FG-Nup dimensions, the polymer dimensions are similar between C/H-variants with the same d_{FG}, and the variability in Stokes radii between designs is small (~10%).

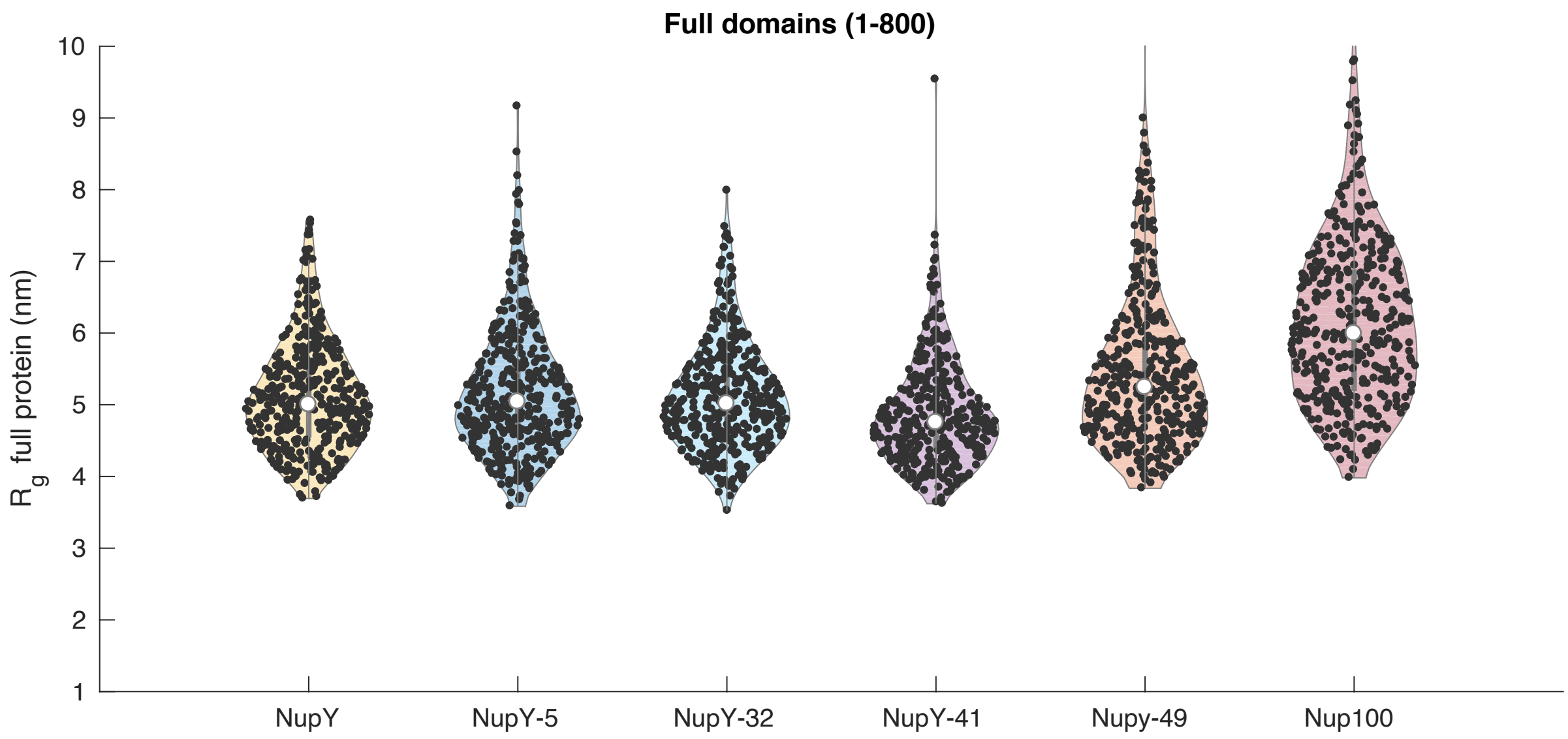

Supplementary Figure 3 – Violin plots of the radii of gyration of 5 baseline NupY-designs, compared to Nup100.
The consistency in mean radius of gyration (white dot) is high amongst the five baseline NupY designs, here simulated without the leading Gly-Phe and trailing Cys residues to match the length of Nup100. Nup100, the native GLFG-Nup closest to NupY (same domain lengths), adopts more extended configurations than the native NupY design. Total simulation time is 1500 ns, of which the final 1000 ns were used. For visualization purposes, each 50th data point is shown (black dots).

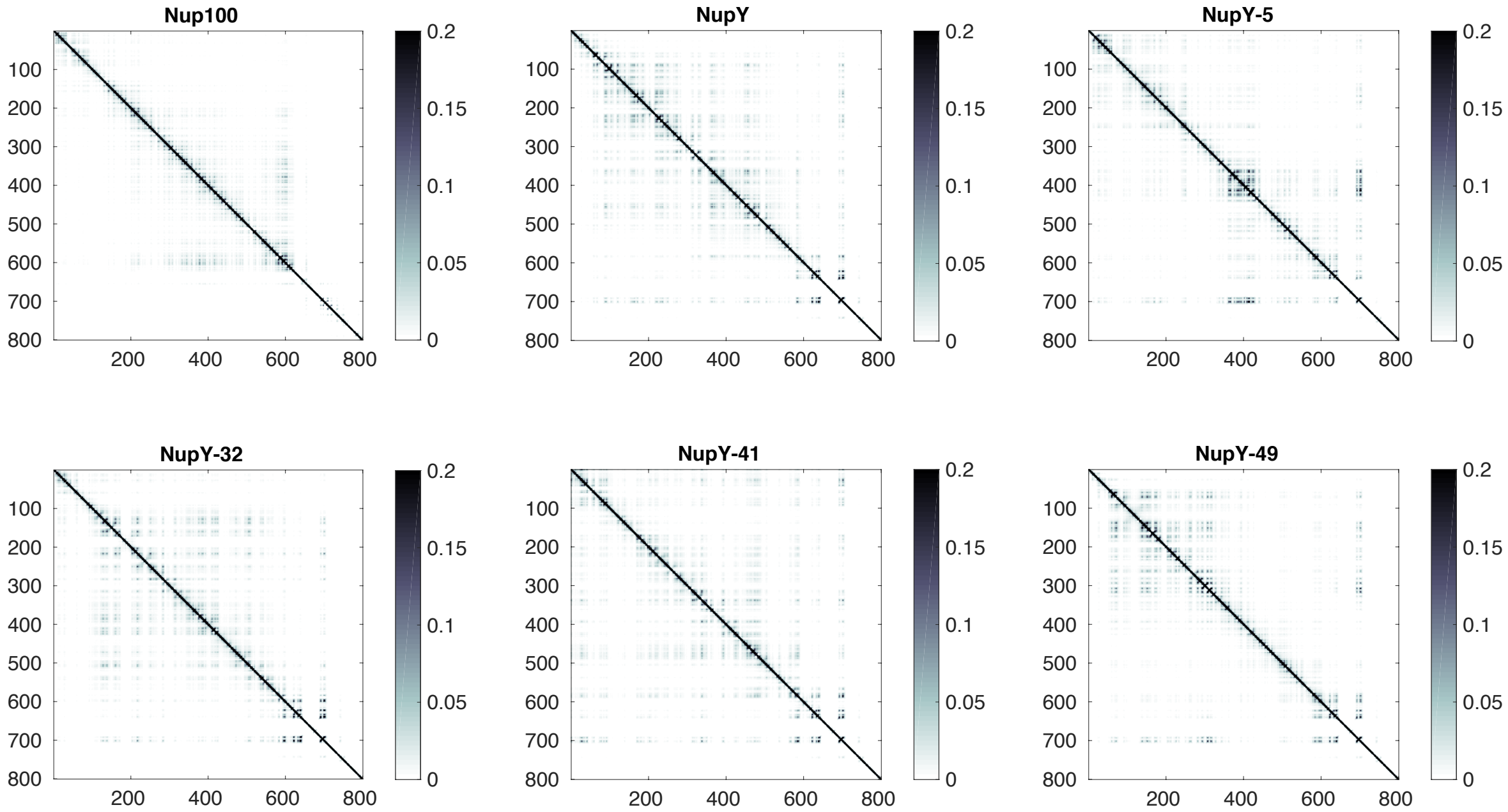

Supplementary Figure 4 – Inter-residue contact maps for NupY base design variants and Nup100.

Compared to the native Nup100, the 5 NupY baseline variants display more compaction (Supplementary Figure 3). Following a contact analysis (see Methods), we identify two root causes for the differences. On the one hand, the higher degree of patterning of GLFG and FG-motifs in the NupY-variants causes additional compaction as compared to the more random patterning in the Nup100 collapsed domain. Additionally, a number of motifs exist in the extended domain of the NupY-variants (residues 611-800 in this simulation, Gly-Phe and Cys residues were left off) that interact with the GLFG/FG-motifs in the collapsed domain, an effect not seen as explicitly in Nup100.

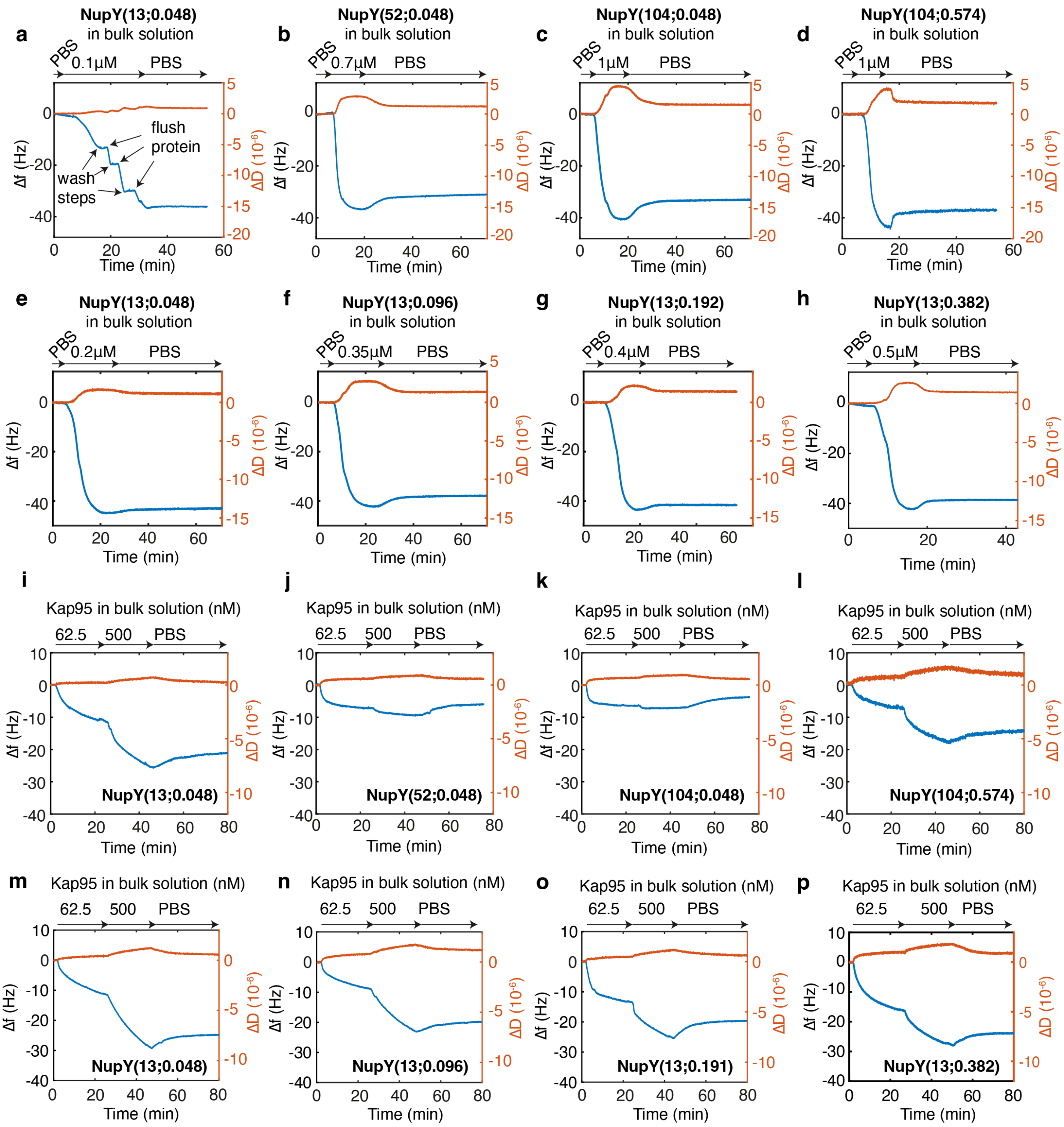

Supplementary Figure 5 – Overview of QCM-D experiments on NupY brushes.
a-h) Coating of the QCM-D sensor with the indicated NupY variants. The concentration was individually adjusted to achieve similar frequency response Δf for all variants. **i-p)** Addition of Kap95 to the pre-formed NupY brushes at concentrations of 62.5 and 500 nM shows different degree of interaction for the tested NupY variants.

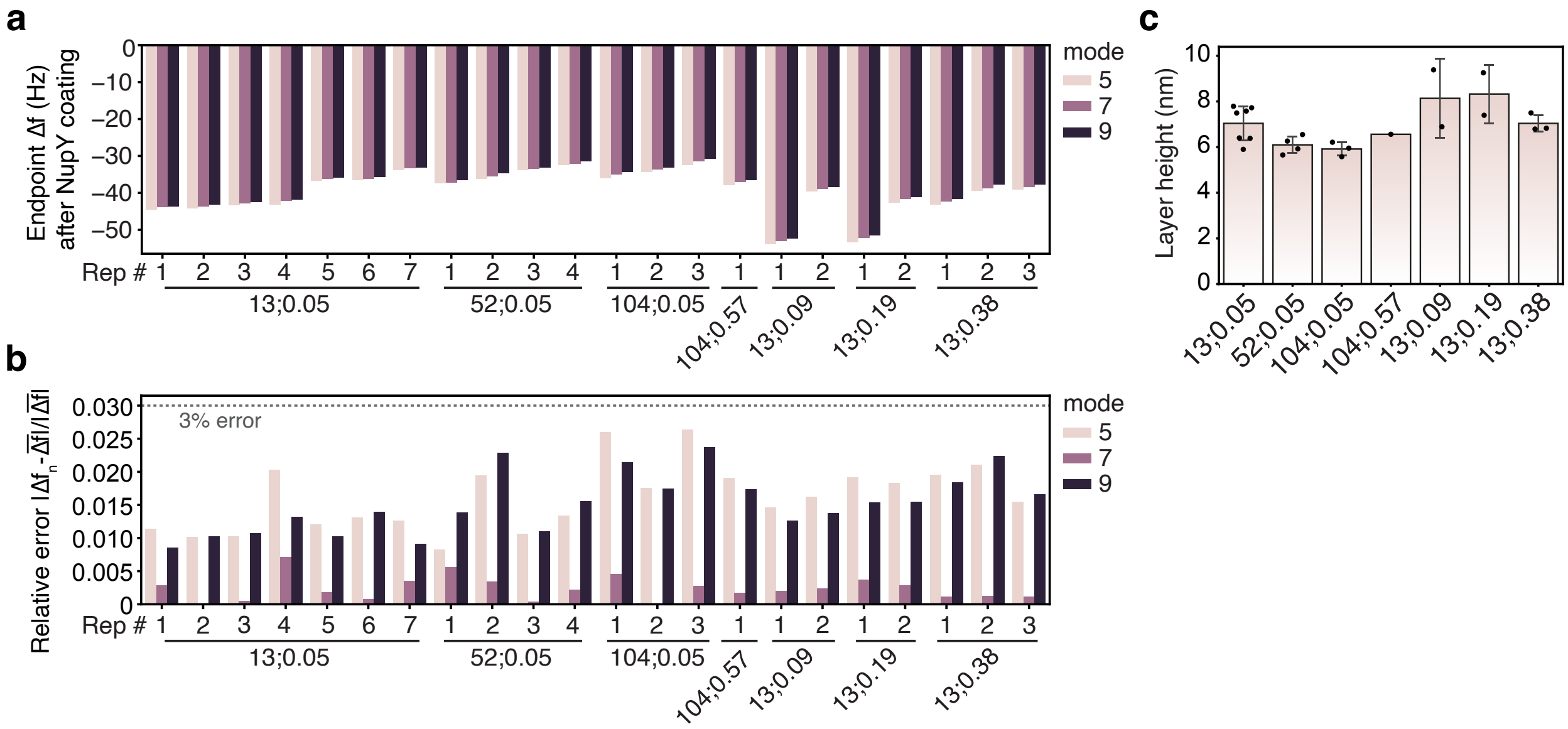

Supplementary Figure 6 – Harmonic spread of final Δf after NupY coating and layer height estimation.

a) Endpoint frequency shifts for the different NupY variants for the indicated replicates. Only replicates with final Δf between -30 and -55 Hz were included. **b)** Relative error between each mode and the within-replicate mean, overall featuring a small (<3%) spread across harmonics 5th, 7th, and 9th for all analyzed replicates. **c)** Layer height estimation from Sauerbrey relation (Methods) assuming proper layer hydration (ρ=1 g/cm^3) and film uniformity (justified by the measured low harmonic spread **(**see panel **b)**).

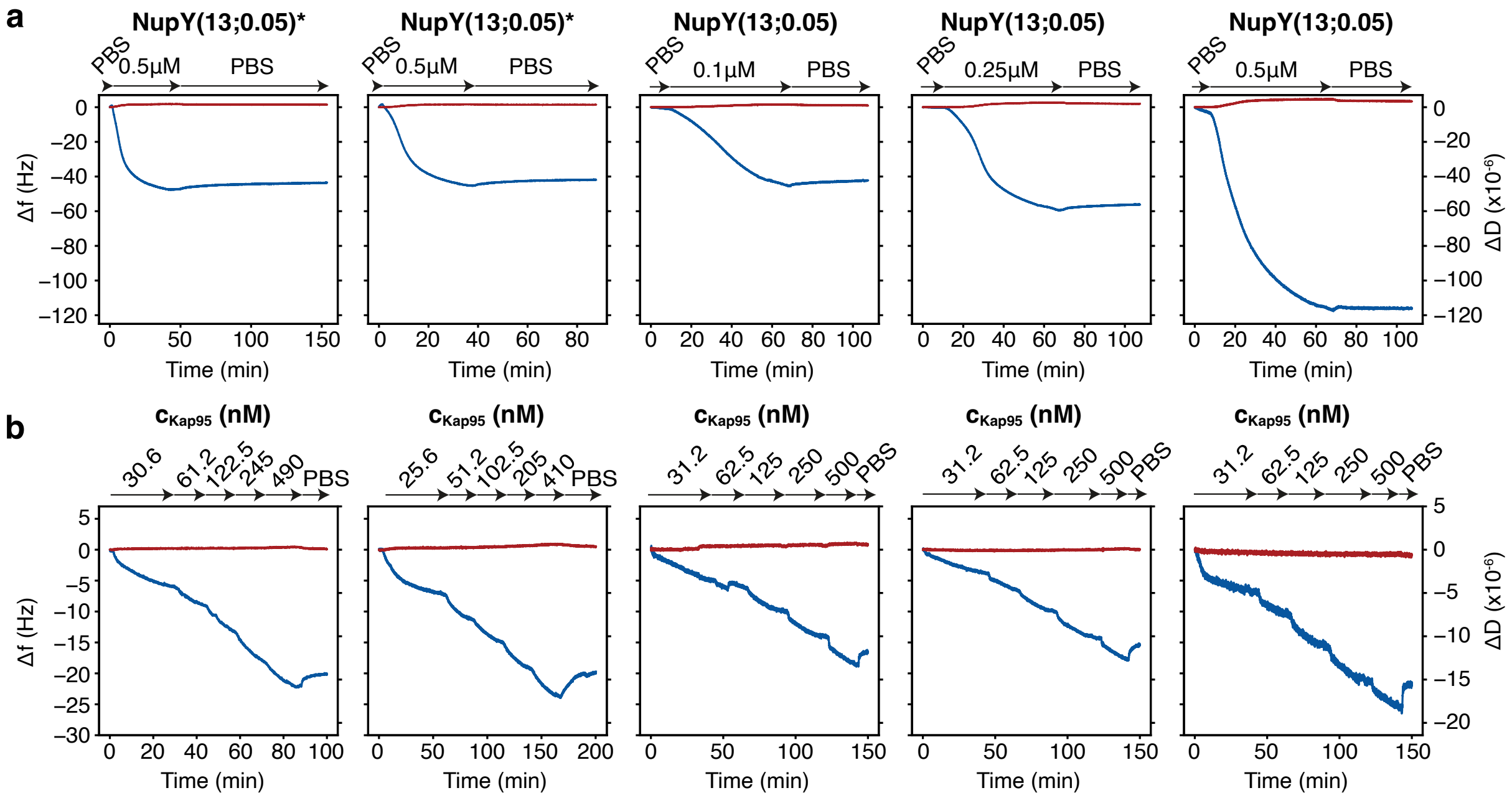

Supplementary Figure 7 – Robustness of Kap95 binding to NupY(13;0.05) for brushes with different final Δf.

a) Coating of the QCM-D sensor with the NupY(13;0.05) at the indicated concentrations. Asterisk indicates an older NupY stock for which a higher nominal concentration was estimated. **b)** Addition of Kap95 to the pre-formed NupY brushes at the indicated increasing concentrations in the range ~30-500 nM, showing that similar Kap95 surface binding was achieved for NupY(13;0.05) brushes with different final Δf. Red lines indicate dissipation shifts (ΔD), blue lines frequency shifts (Δf).

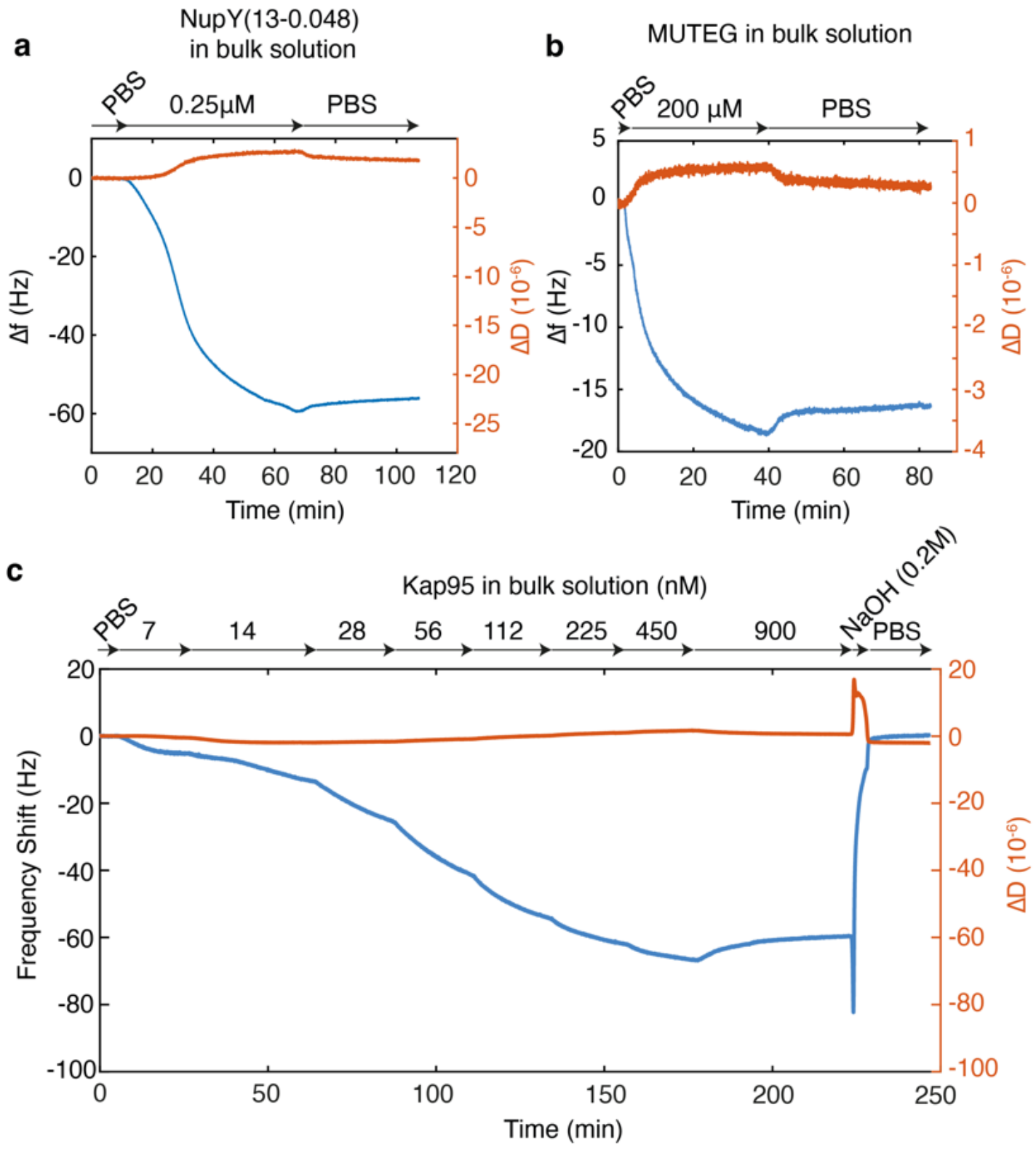

Supplementary Figure 8 – MUTEG coating and NaOH dissociation of NupY-bound Kap95 in QCM-D experiments.

a) Coating of a sensor with our template NupY(13;0.05) at 0.25 μM concentration. **b)** Passivating with MUTEG at 200 μM of the remaining exposed gold surface following NupY coating. **c)** Titration of Kap95 from concentrations 7–900 nM showing efficient binding to the NupY(13;0.05) brush followed by complete dissociation by 0.2 M NaOH.

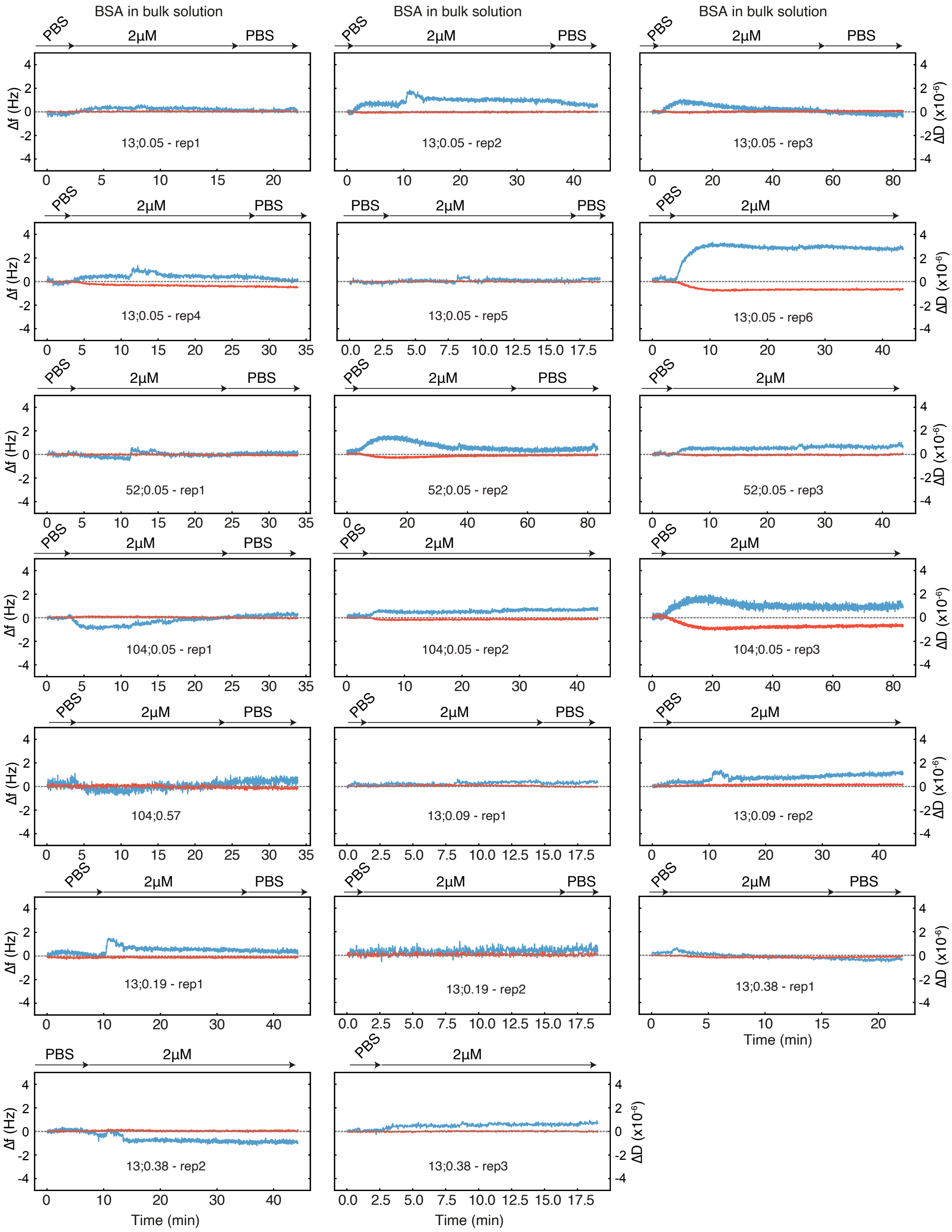

Supplementary Figure 9 – Response of NupY-coated QCM-D chips to BSA.
Upon flushing high concentration (2 μM) of BSA onto the NupY-coated sensors only subtle changes in the resonance frequency or dissipation were detected, which could result in either positive or negative shifts, indicating minor interactions between all NupY variants and BSA.

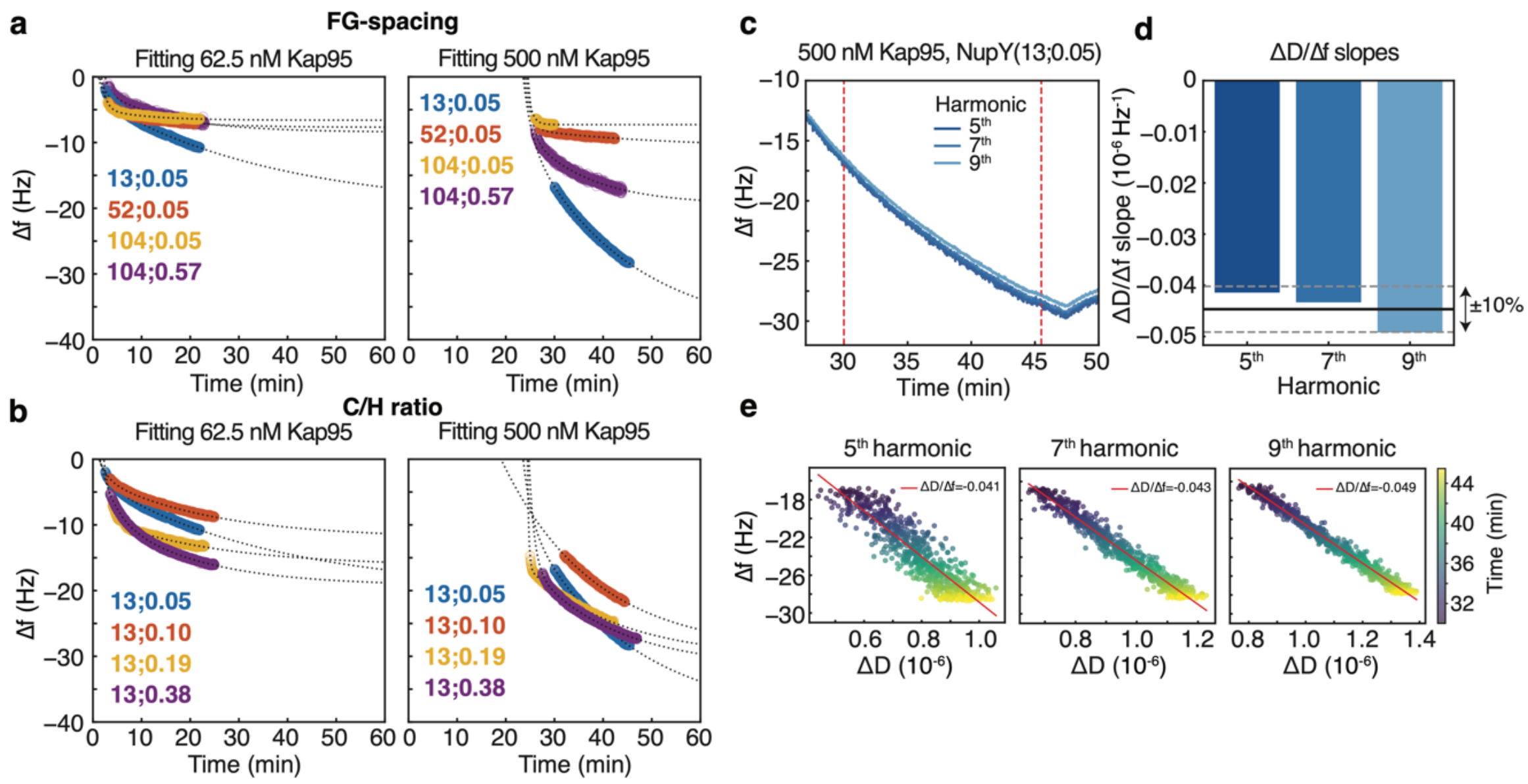

Supplementary Figure 10 – Fitting of the QCM-D frequency response upon Kap95 binding. The frequency shift Δf for the FG-spacing (**a**) and C/H variants (**b**) after sequential flushing of 62.5 and 500 nM Kap95 was fitted to a bi-exponential model function to extract the half-time $t_{1/2}$ and saturation value Δf_{max}. **c)** Frequency shift upon binding of 500 nM Kap95 onto a NupY(13;0.05) brush with indicated harmonics 5th, 7th, and 9th. Red dashed lines indicate the interval where the fit was performed. **d)** Bar plot showing the slopes ΔD/Δf for different harmonics, obtained by fitting a linear regression through the Δf *vs* ΔD data points within the time interval indicated in (**c**) and taking the inverse of that. **e**) Scatterplot showing Δf *vs* ΔD data points within the time interval highlighted in (**c**). Fitted linear regression is shown as a red line. Extracted slope ΔD/Δf values are indicated.

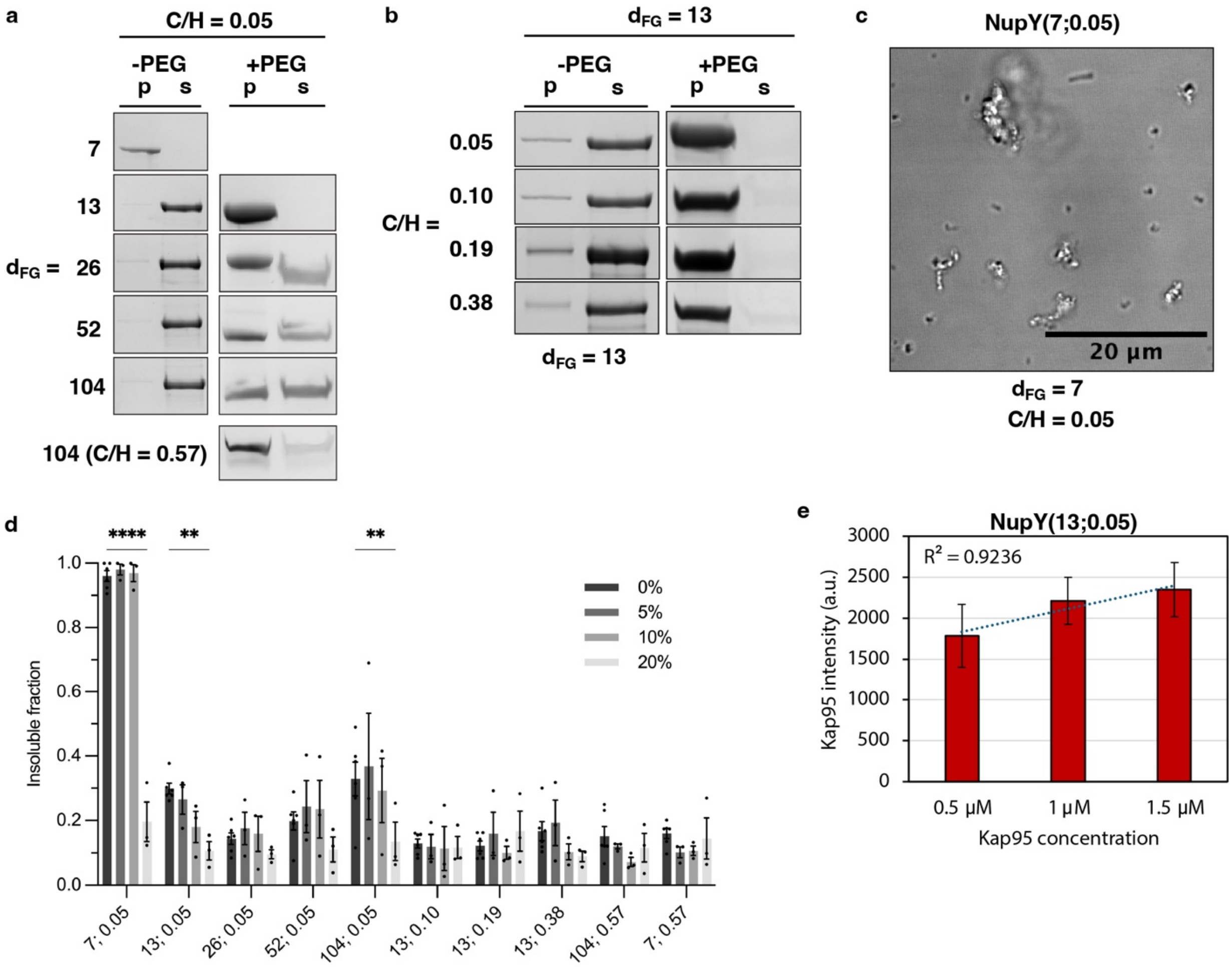

Supplementary Figure 11 – Characterization of the liquid-liquid phase separation propensity and aggregation of the different NupY variants.

a-b) The degree of condensation at a concentration of 200 nM in the absence or presence of 10% w/v PEG-8000 in 50 mM Tris, 150 mM NaCl, pH 7.5 is assessed by a sedimentation assay. p: pellet, s: supernatant. **c)** The variant NupY(7;0.05) formed amorphous aggregates at 200 nM in the absence of PEG. **d)** Quantification of the insoluble fraction of NupY condensates formed at room temperature after 1 hour (3µM protein in 50 mM Tris–HCl pH 8.0, 150 mM NaCl, and indicated concentrations of 1,6-hexanediol). Graph shows mean ± SEM (n=3-6). Two-way ANOVA with Dunnett's multiple comparisons test compared to 0% 1,6-hexanediol for each NupY variant. **** $p < 0.0001$, ** $p < 0.01$ **e)** Saturation of condensates formed by the variant NupY(13;0.05) under increasing concentration of Kap95. The marginal increase from 1 µM to 1.5 µM indicates that condensates are close to reaching saturation under the experimental conditions.

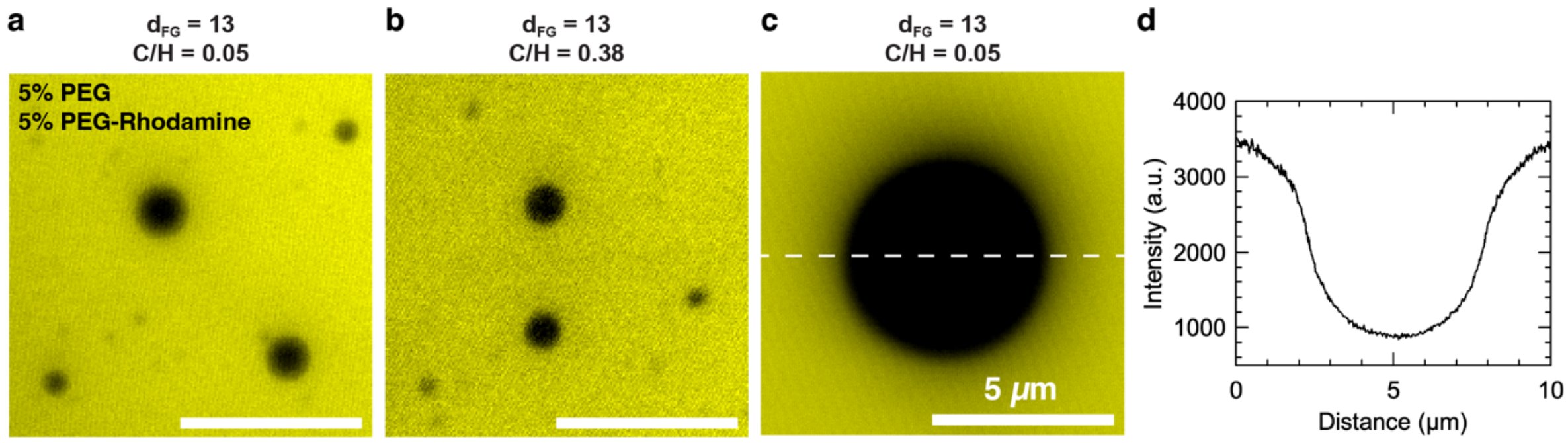

Supplementary Figure 12 – PEG does not partition into NupY condensates. Condensates of the template NupY(13;0.05) and variant NupY(13;0.38) were formed in 5% w/v PEG-8000 and 5% w/v PEG-8000-Rhodamine in 50 mM Tris, 150 mM NaCl, pH 7.5. PEG is excluded from the condensates. a-c) Confocal fluorescence images of NupY condensates. Scalebar in a-b: 10 µm. d) Line profile of the PEG-Rhodamine intensity as indicated in c.

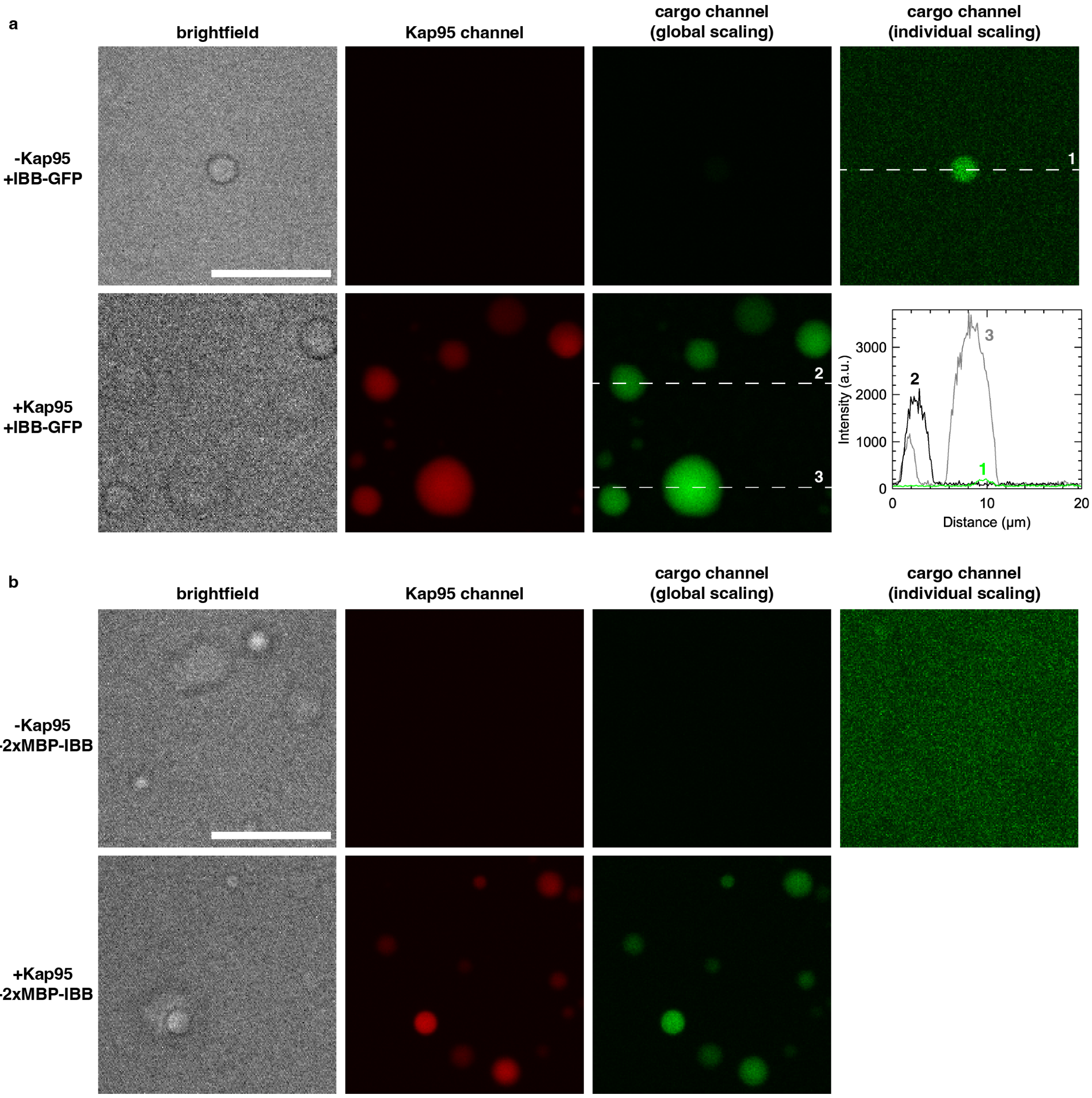

Supplementary Figure 13 – Minimal interactions of model cargoes with NupY condensates. Condensates of the template NupY(13;0.05) were formed in 10% w/v PEG-8000 in 50 mM Tris, 150 mM NaCl, pH 7.5, and supplemented by 1 µM cargo (**a**: IBB-GFP, **b**: 2xMBP-IBB) in the absence (top) or presence (bottom) of 1 µM Kap95. Scalebars: 10 µm. The cargo channel in the presence and absence of Kap95 is shown in global scaling between the two conditions (third column) and individually scaled (fourth column). A minimal amount of interaction is detected for the IBB-GFP cargo (**a**, right) which is negligible compared to the signal in the presence of Kap95 (see line profiles). No significant interaction is detected for the large cargo 2xMBP-IBB.

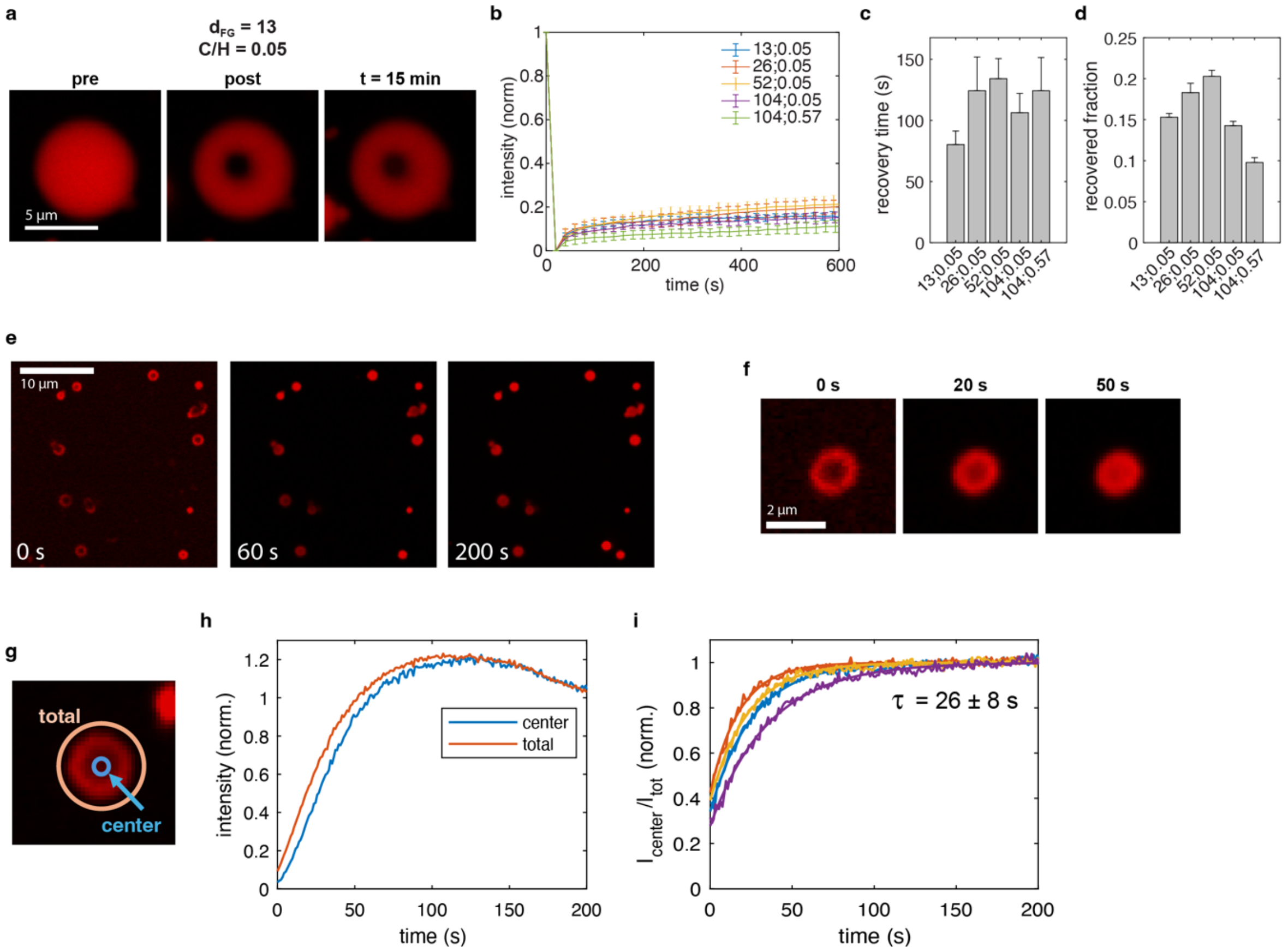

Supplementary Figure 14 – Diffusion kinetics of Kap95 in NupY condensates. Condensates of the different NupY variants were formed at 200 nM in 10% w/v PEG-8000 in 50 mM Tris, 150 mM NaCl, pH 7.5. **a)** FRAP experiment of a condensate formed by the template NupY loaded with 1 µM Kap95. Minimal recovery occurs over the timescale of 15 min. **b)** Recovery curves of the different NupY variants showing low recovery over 10 min. **c-d)** Recovery time and recovered fraction of the data shown in b. **e)** An influx experiment where Kap95 was added to fresh condensates of the template $NupY_{13\text{-}0.05}$ at a concentration of 1 µM. The contrast of the images was individually adjusted. **f)** Zoom-in of an exemplary particle from e. **g)** The influx of Kap95 was quantified by comparing the the total intensity to the intensity in the center of the condensate. **h)** Exemplary quantification of the influx as shown in h. Curves were each normalized to their respective signal at the end of the experiment. **i)** Comparison of the influx curves of four different particles. Each curve was normalized to the intensity ratio at the end of the experiment. Kap95 influx occurs on the timescale of 26±8 s.

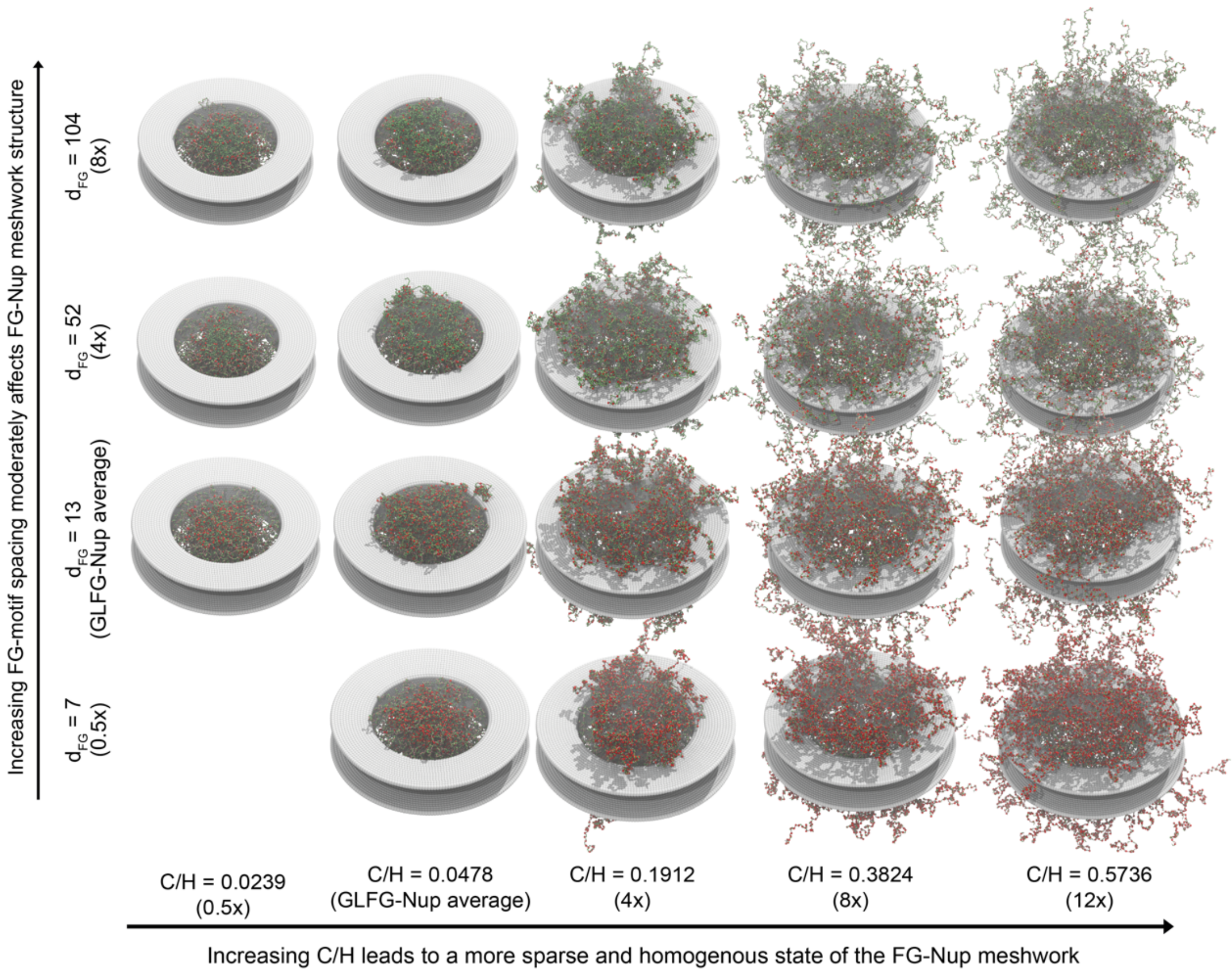

Supplementary Figure 15 – Snapshots of nanopores coated with FG-spacing and C/H-variants. Generally, the distribution of FG-Nups (green) and FG-motifs (red) is strongly affected by the value of C/H (horizontal axis). For low C/H-values, our FG-Nups seal the pore interior, where the collapsed domains (residues 1-610) form dense regions near the pore center. At larger values of C/H (beyond 4x the native average or 0.1912) a homogenous and sparse meshwork of FG-Nups forms, extending outwards from the pore region. The distribution of FG-Nups is less strongly affected by the FG-spacing (vertical axis): in general, the distribution of FG-Nups remains similar for FG-spacing variants, as the patterning of hydrophobic and C/H is conserved. The 0.5x-spacing (d_{FG}=7)-variants provide an exception to this rule, as the enhanced patterning of hydrophobic residues leads to an additional collapse of the FG-Nup meshwork for all variants.

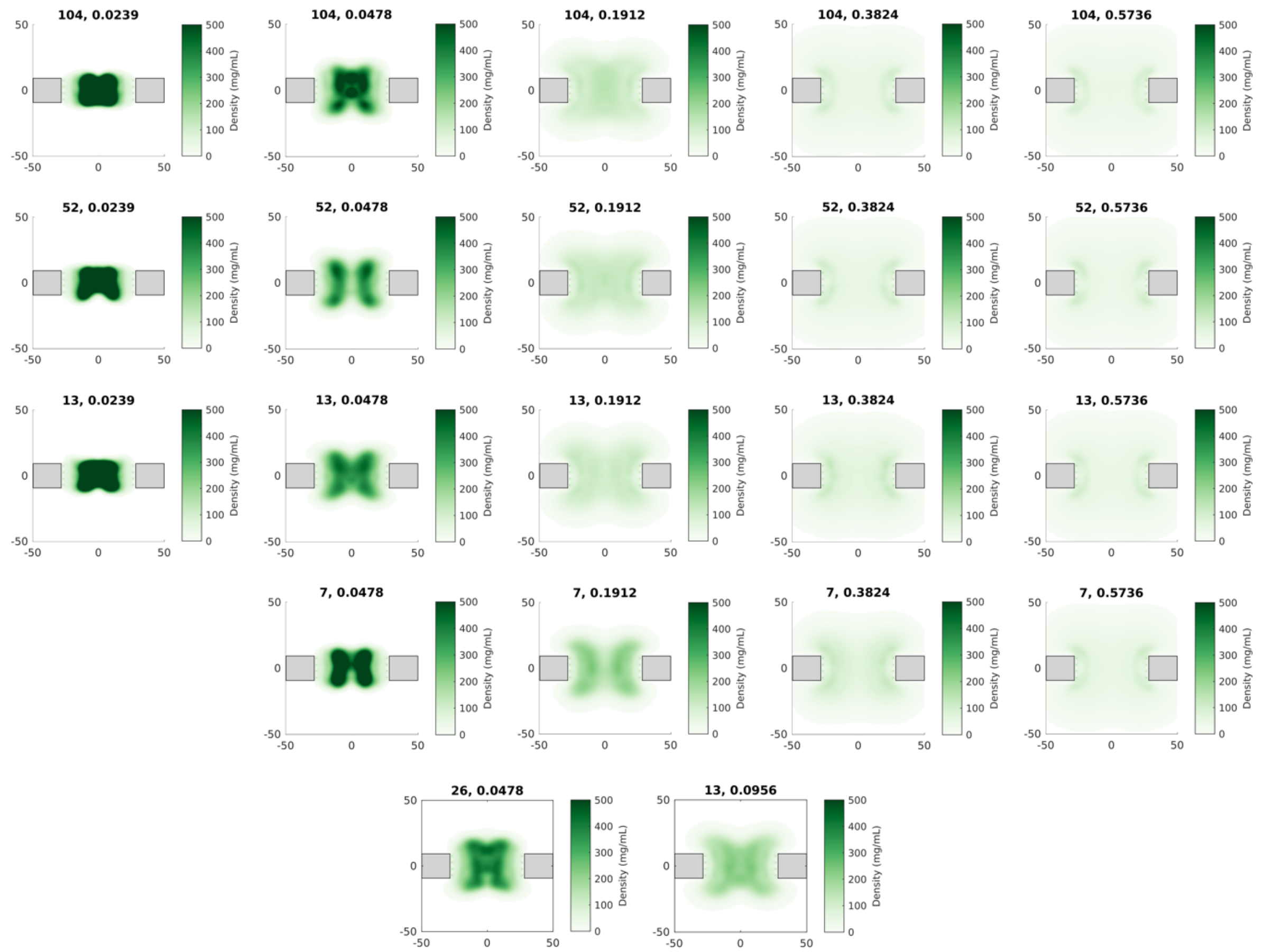

Supplementary Figure 16 – Time-averaged, axi-radial density graphs of FG-Nups in nanopores coated with artificial FG-Nups, grouped by C/H and FG-spacing. Scaffolds are highlighted in gray. Additional variants with 2xFG-spacing and 2xC/H are shown separately on the bottom row. The value of C/H (horizontal direction) strongly affects the distribution of FG-Nups in the nanopore lumen: for the native GLFG-Nup average (0.0478), a dense, ring-like or 'doughnut-like' structure is formed by the FG-Nups, with densities exceeding 500 mg/mL. A further reduction in C/H (0.0239) leads to a strongly collapsed meshwork with densities exceeding 1000 mg/mL. Increases (C/H=0.0956 and higher) lead to the formation of a more homogenous meshwork, where the density inside the pore lumen steeply drops, from 300 mg/mL to ~100 mg/mL. Increases in FG-spacing (vertical direction) w.r.t. the native GLFG-Nup average (dF_G=13) do not notably affect the distribution of the FG-Nups, as the patterning of hydrophobic residues and overall C/H are conserved. For the variants with a decreased spacing (d_{FG}=7, top row), we find that regardless of conserved C/H, the increased number and degree of patterning of hydrophobic residues leads to an additional collapse of the FG-Nup mesh.

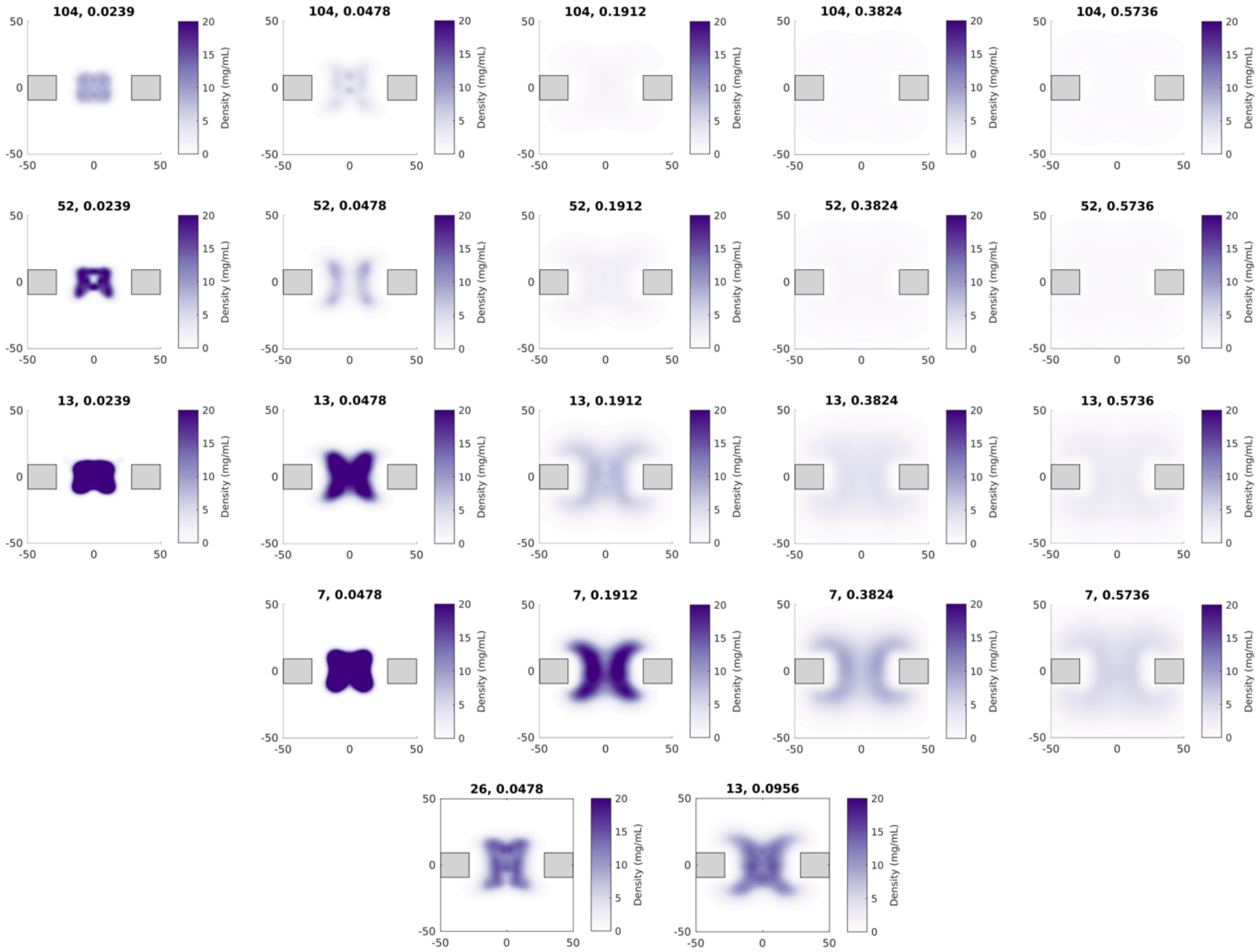

Supplementary Figure 17 – Time-averaged, axi-radial density graphs of F(G)-motifs in nanopores coated with artificial FG-Nups, grouped by C/H and FG-spacing. Scaffolds are highlighted in gray. Additional variants with 2xFG-spacing and 2xC/H are shown separately on the bottom row. The FG-motif distribution coincides with the distribution of the NupY-domains that are varied in this study (residues 1-610). Changes in the localization of this domain by virtue of changing C/H (horizontal direction) similarly affect the localization of the FG-motifs, where a dense, ring-like or 'doughnut-like' central region is formed that comprises all the FG-motifs for low values of C/H, and the FG-motifs are spread out rather evenly for higher C/H-values, albeit with a significantly reduced density. Changes in the FG-spacing (vertical direction), which occur under similar C/H, generally lead to a preserved shape of the FG-motif distribution. At the same time, the density of the FG-motifs in the nanopore scales with the change in d_{FG}: compared d_{FG}=13, variants with spacings of 26, 52 and 104 residues display densities that are approximately 2, 4 and 8 times lower, respectively. This trend does not hold fully for a reduced FG-spacing (d_{FG}=7, top row), as the FG-Nup meshwork experiences an additional collapse by means of the enhanced patterning of hydrophobic residues.

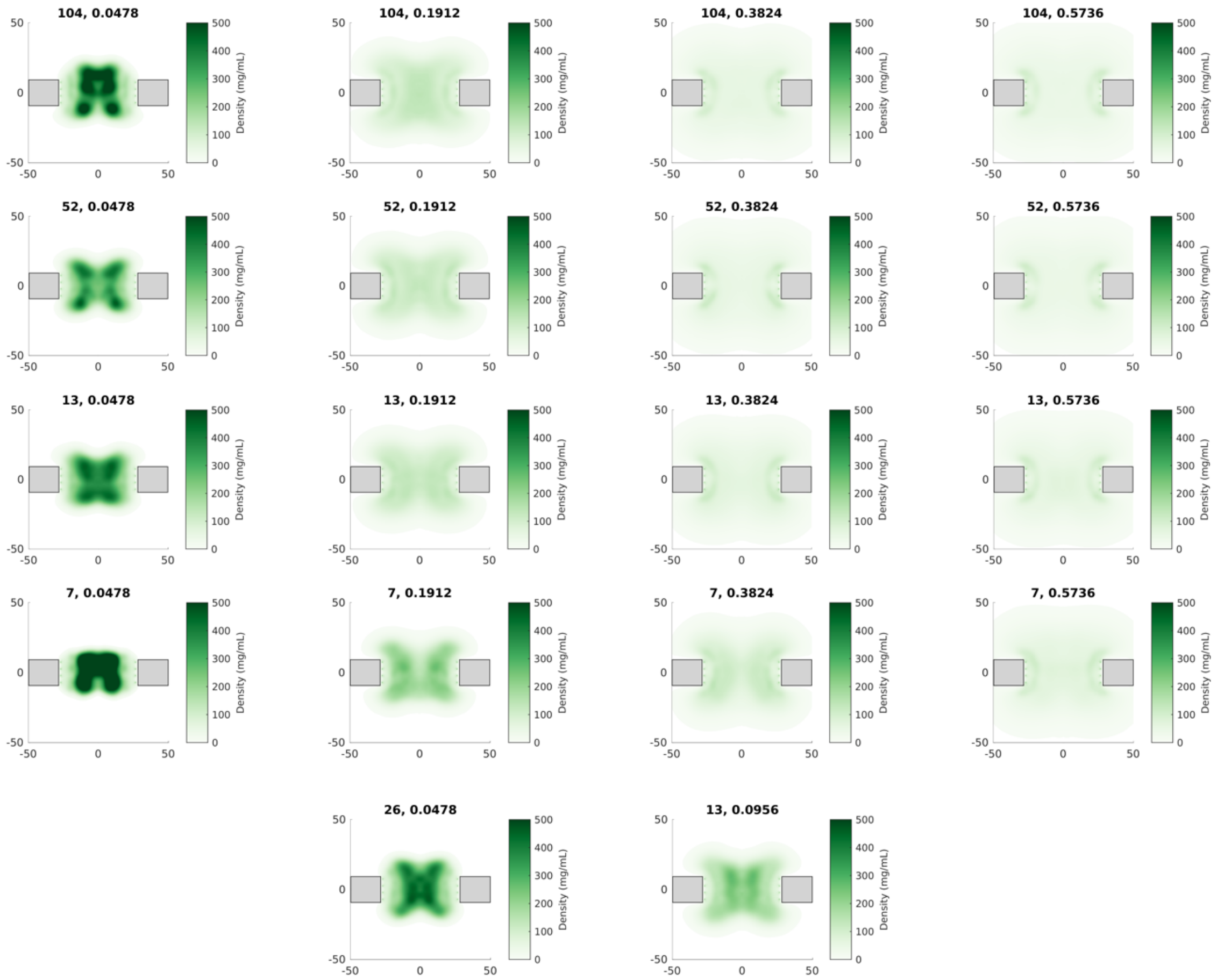

Supplementary Figure 18 – Time-averaged, axi-radial density graphs of FG-Nups in nanopores coated with artificial FG-Nups in presence of Kap95 and inert proteins, grouped by C/H and FG-spacing. Scaffolds are highlighted in gray. Additional variants with 2xFG-spacing and 2xC/H-ratio are shown separately on the bottom row. C/H=0.0239 (0.5x native average) variants were not studied in presence of cargo and NTRs due to their high FG-Nup densities. The overall FG-Nup density distributions closely follow those in Supplementary Figure 16, indicating that the presence of the studied amount of NTRs and inert molecules (10 copies of Kap95, BSA and Ubiquitin, resp.) did not appreciably alter the average structure of the FG-Nup meshwork.

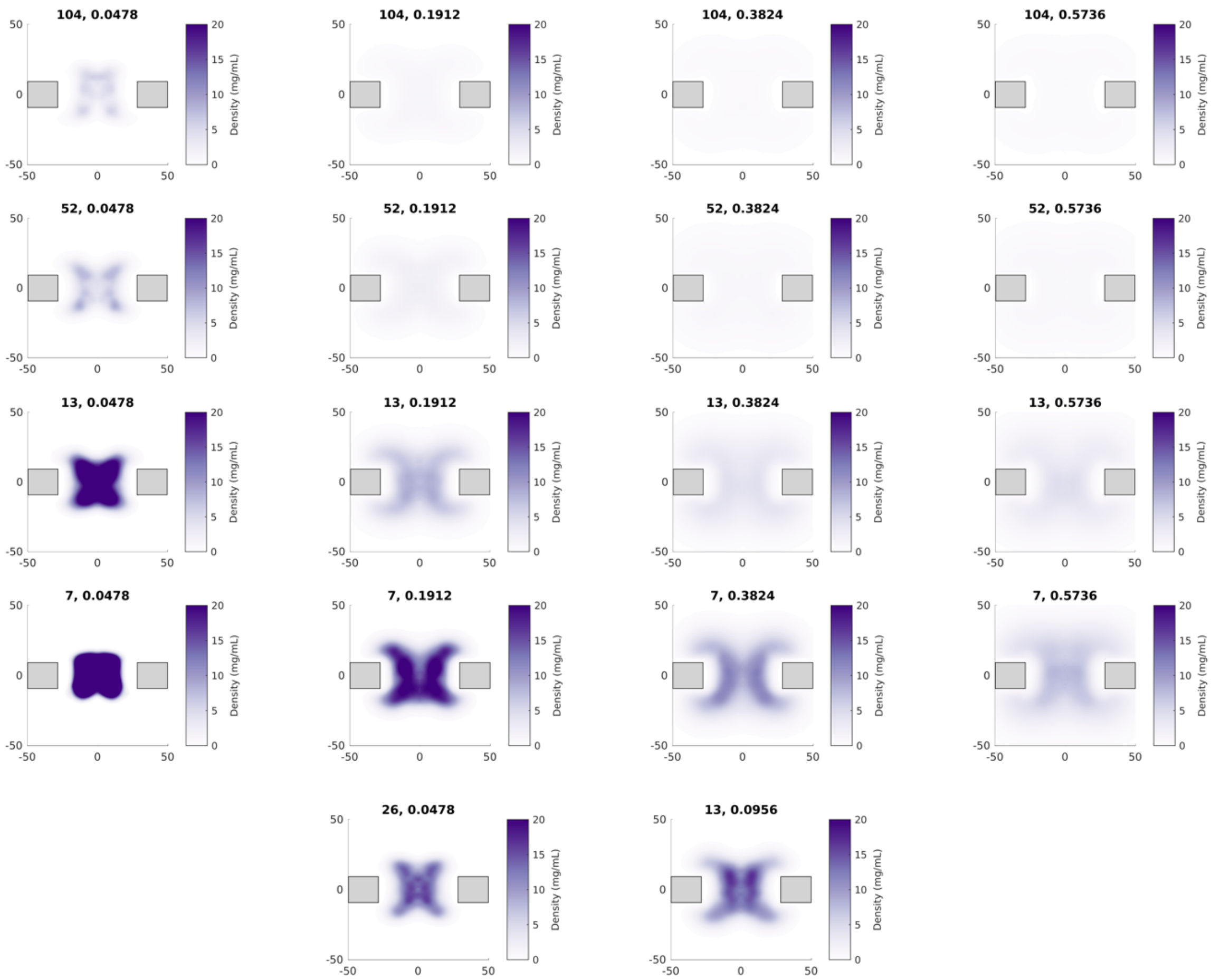

Supplementary Figure 19 – Time-averaged, axi-radial density graphs of the F(G)-motifs in nanopores coated with artificial FG-Nups in presence of Kap95 and inert proteins, grouped by C/H and FG-spacing. Scaffolds are highlighted in gray. Additional variants with 2xFG-spacing and 2xC/H-ratio are shown separately on the bottom row. C/H=0.0239 (0.5x native average) variants were not studied in presence of cargo and NTRs. Similar to the overall FG-Nup density distribution (Supplementary Figure 16), we do not find that the presence of NTRs and inert proteins has not notably affected the distribution of FG-motifs.

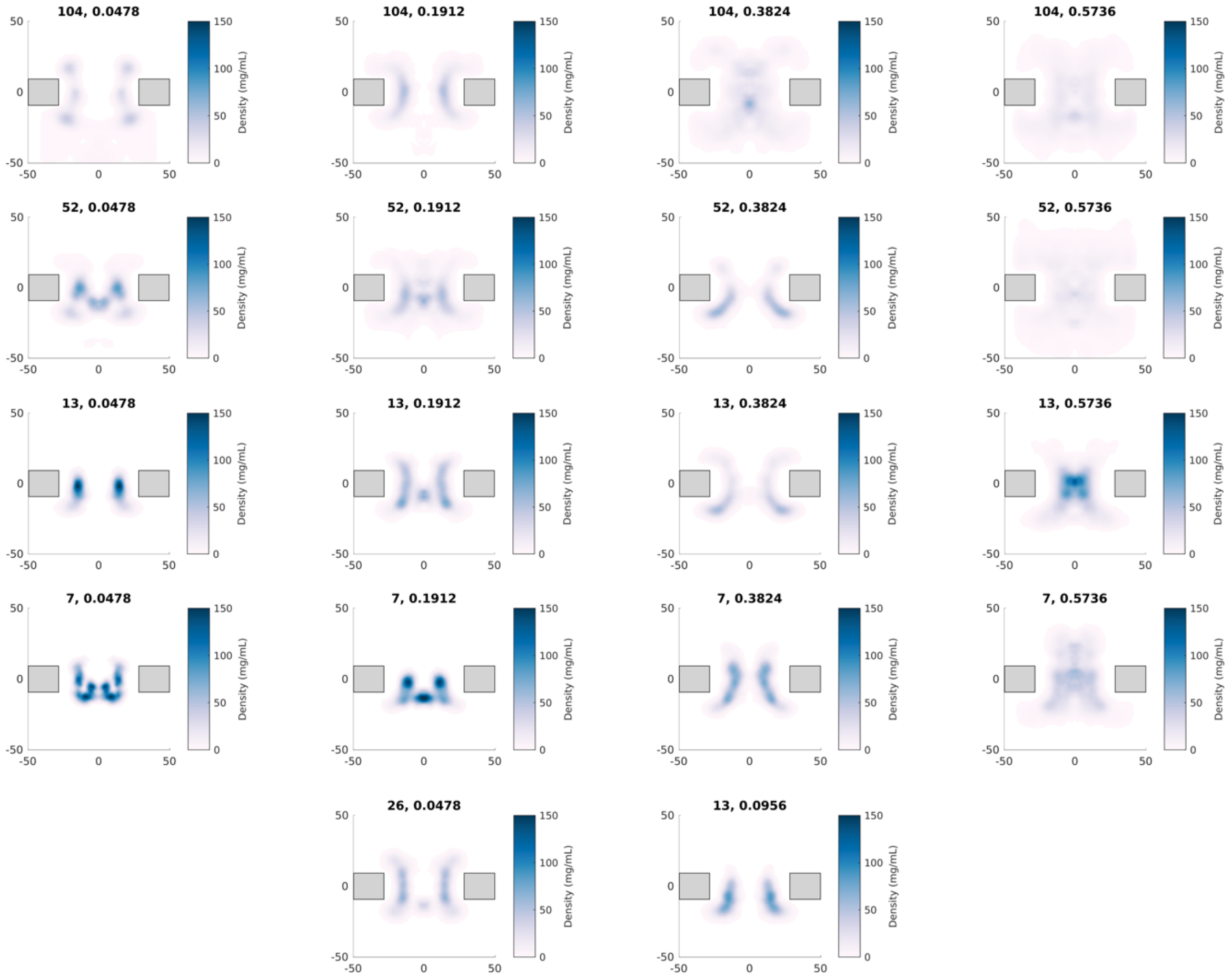

Supplementary Figure 20 – Time-averaged, axi-radial density graphs of the Kap95 protein in nanopores coated with artificial FG-Nups in presence of inert proteins, grouped by C/H-ratio and FG-spacing. Scaffolds are highlighted in gray. Additional variants with 2xFG-spacing and 2xC/H-ratio are shown separately on the bottom row. C/H=0.0239 (0.5x native average) variants were not studied in presence of cargo and NTRs. Following the changes in the FG-Nup distribution (Supplementary Figure 16 with varying C/H (horizontal direction), the distribution of Kap95 changes in a similar fashion. For low values of C/H, Kap95 interacts with FG-motifs in the dense, central bands, ring-like or 'doughnut-like' regions formed by the artificial FG-Nups, without fully localizing inside the dense regions. Indeed, the density distribution of Kap95 for low C/H (left column) does not fully coincide with the density distributions of FG-motifs and FG-Nups in Supplementary Figures 16-17. Rather, it is shifted radially outward. For higher values of C/H (beyond 0.0956), the FG-Nup density reduces, and the Kap95 distribution more closely follows that of the FG-motifs (more homogenous with a slight presence of two 'bands'). Expectedly, the FG-spacing (vertical direction) affects the Kap95 density in the pore lumen: reducing or increasing the FG-spacing leads Kap95 to localize near the densest regions or spread out more evenly throughout the pore region, respectively. Interestingly, increasing the FG-spacing to large values (d_{FG}=104) enhances the portion of the pore volume that is sampled by Kap95.

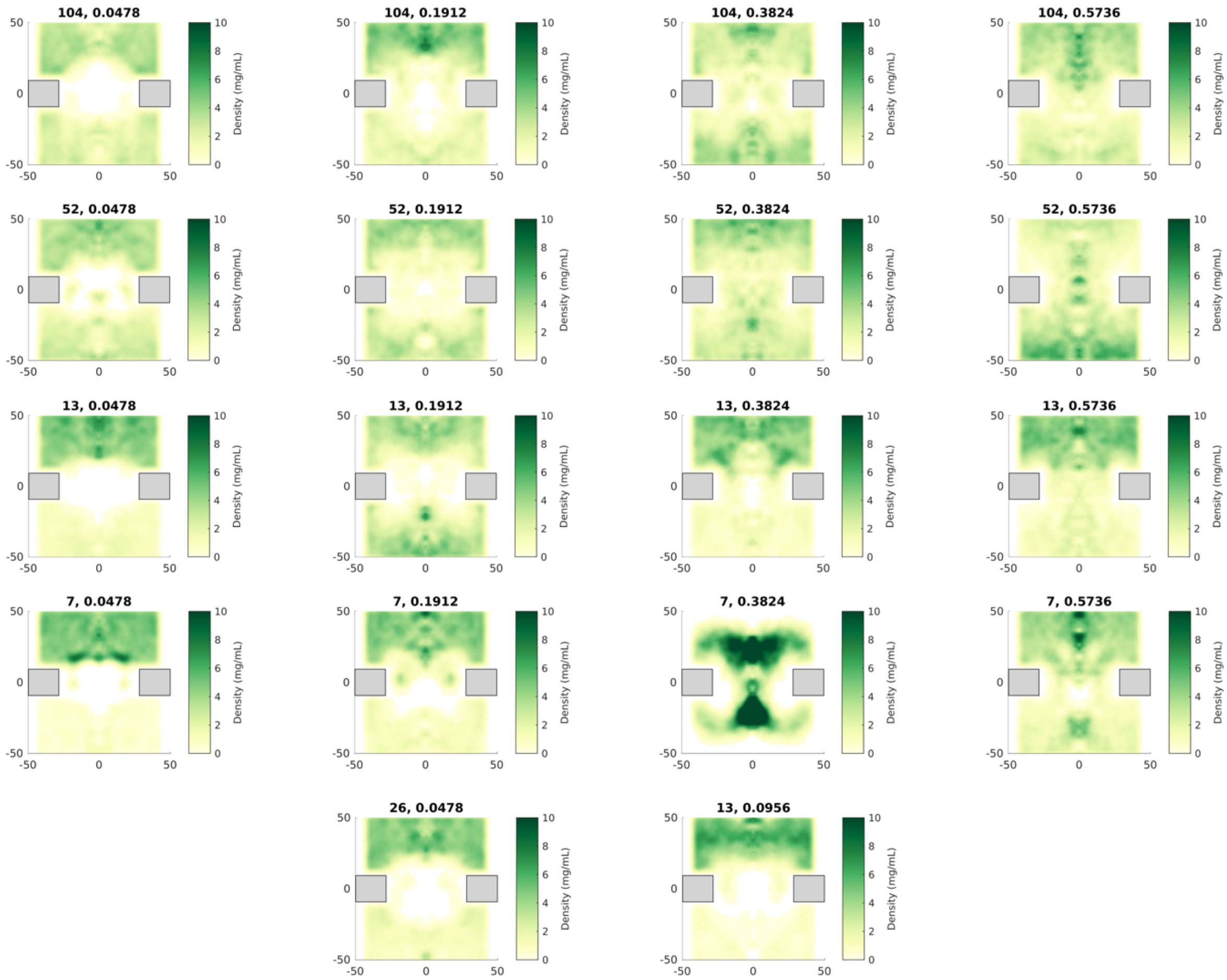

Supplementary Figure 21 – Time-averaged, axi-radial density graphs of the BSA protein in nanopores coated with artificial FG-Nups in presence of Kap95 and inert proteins, grouped by C/H and FG-spacing. Scaffolds are highlighted in gray. Additional variants with 2xFG-spacing and 2xC/H-ratio are shown separately on the bottom row. C/H=0.0239 (0.5x native average) variants were not studied in presence of cargo and NTRs. The density distributions for BSA (initially inserted from the top side of the nanopore, and subsequently expelled to the top side) indicate that changes in C/H (horizontal direction) generally lead to a reduction of barrier function. Any BSA permeation events for lower C/H-values (0.0478, 0.0956, 0.1912) take place via the sparser regions near the pore wall, formed by the extended anchoring domain of our artificial FG-Nups. When C/H is increased further, translocation events start to take place via the central channel of the nanopore as well, as the ‘dense plug’ formed by artificial FG-Nups for low C/H is dissolved. For variations in FG-spacing, only a decrease (d_{FG}=7) seems to affect the distribution of BSA, due to the increased FG-Nup density in such cases.

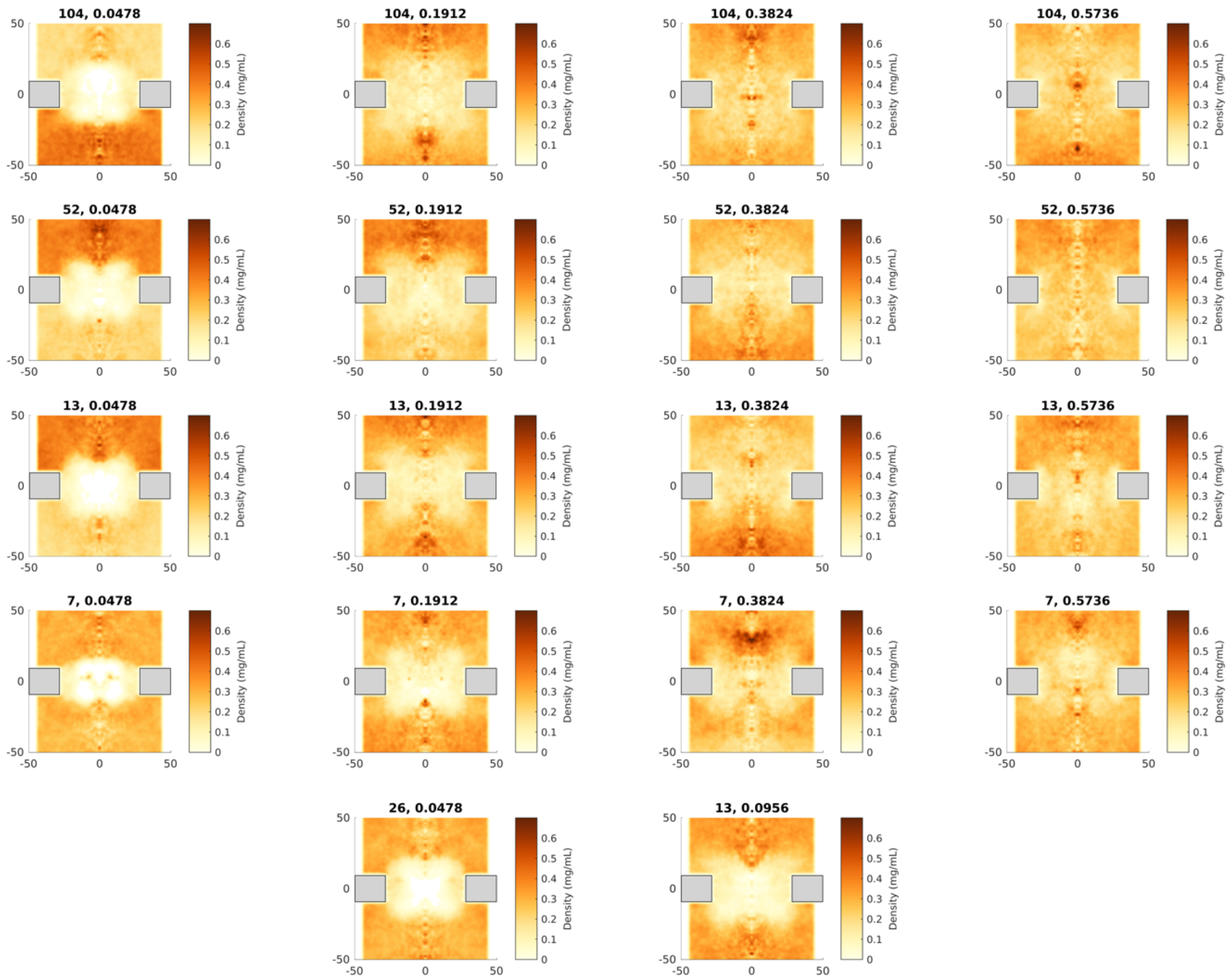

Supplementary Figure 22 – Time-averaged, axi-radial density graphs of the Ubiquitin protein in nanopores coated with artificial FG-Nups in presence of Kap95 and inert proteins, grouped by C/H and FG-spacing. Scaffolds are highlighted in gray. Additional variants with 2xFG-spacing and 2xC/H-ratio are shown separately on the bottom row. C/H=0.0239 (0.5x native average) variants were not studied in presence of cargo and NTRs. Similar to the results for BSA (Supplementary Figure 21), the permeability of FG-Nup-coated nanopores for small inert proteins increases with C/H (horizontal direction). Localization of Ubiquitin in FG-Nup coated pores takes place near the sparse, extended anchoring domains close to the pore wall for low C/H (0.0478, 0.0956). As the value of C/H increases beyond 0.0956, Ubiquitin localizes rather evenly throughout the nanopore system. The effect of FG-spacing on the distribution of Ubiquitin is modest, with only a reduction (d_{FG}=7) w.r.t. the native GLFG-Nup average leading to a noticeable change in the average Ubiquitin distribution.

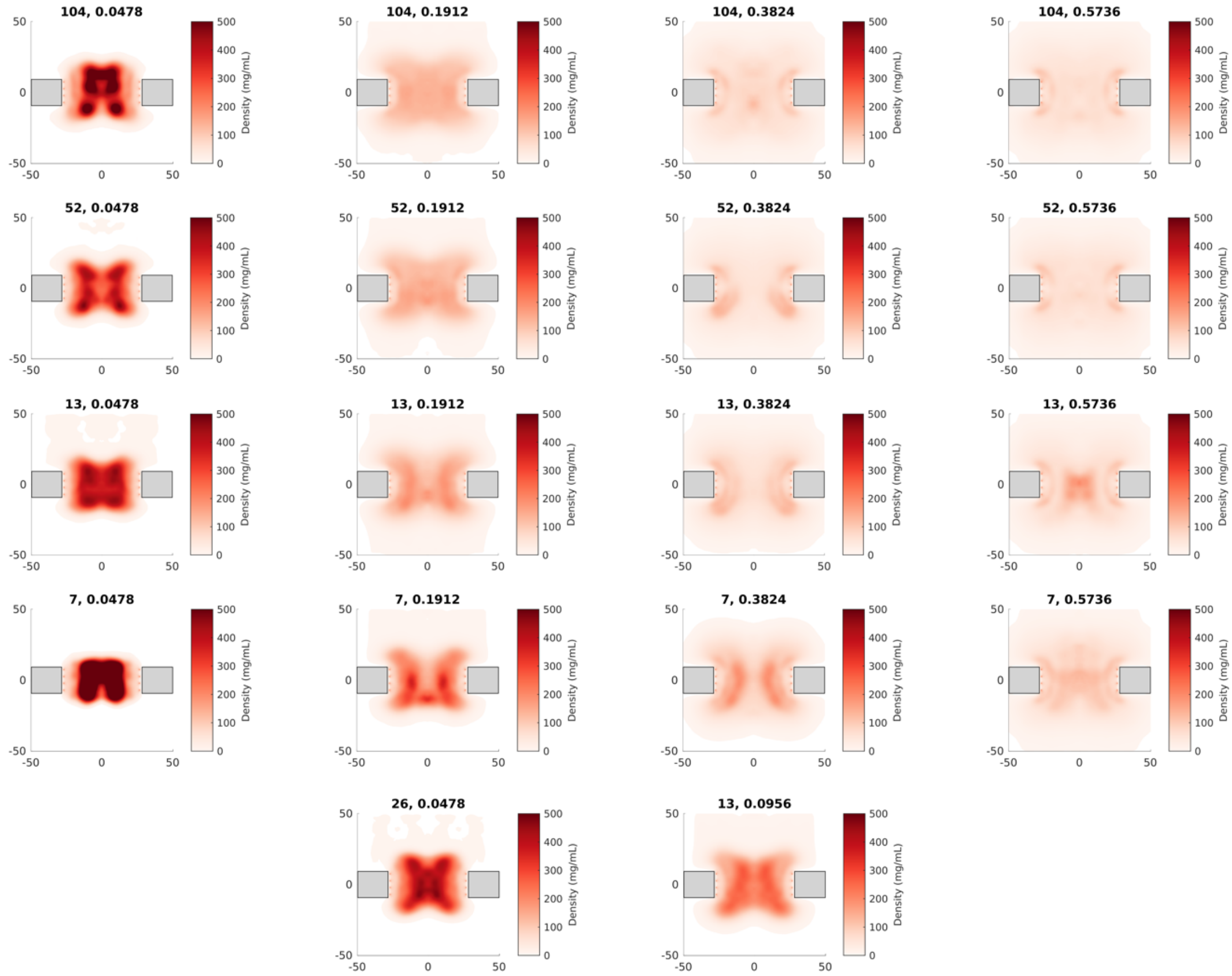

Supplementary Figure 23 – Time-averaged, axi-radial density graphs of the entire protein mass (FG-Nups and inert proteins and Kap95) in nanopores coated with artificial FG-Nups in presence of Kap95 and inert proteins, grouped by C/H-ratio and FG-spacing.

Scaffolds are highlighted in gray. Additional variants with 2xFG-spacing and 2xC/H-ratio are shown separately on the bottom row. C/H=0.0239 (0.5x native average) variants were not studied in presence of cargo and NTRs.

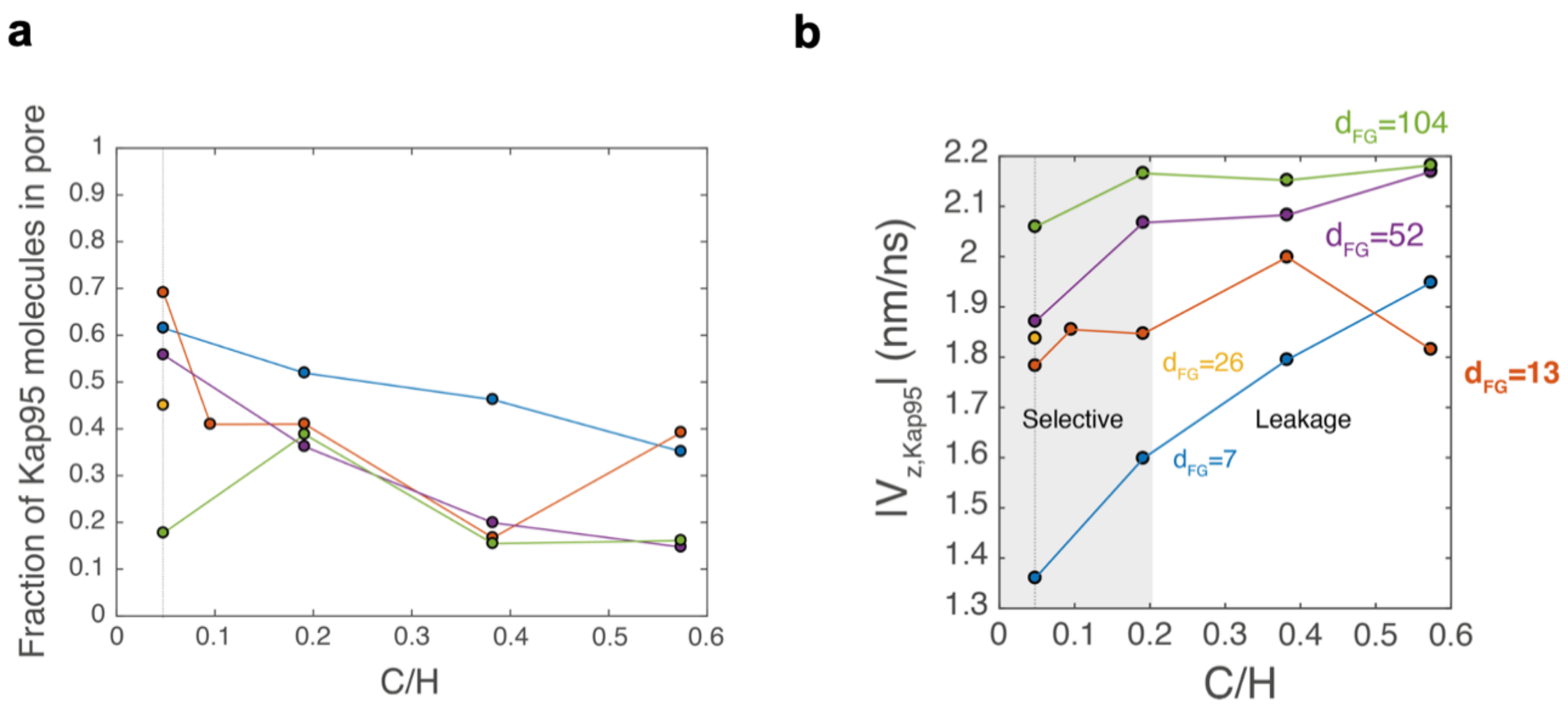

Supplementary Figure 24 – Occupancy and on-axis velocity magnitude of Kap95. **a:** Time-averaged fraction of Kap95 molecules that reside within the pore. With increasing C/H, the occupancy decreases. **b**: Velocity magnitude, averaged over the pore interior and over time, for Kap95 molecules in NupY-variant-coated pores. Mobility of Kaps increases with C/H (reduced steric hindrance) and with dF_G (less frequent binding).

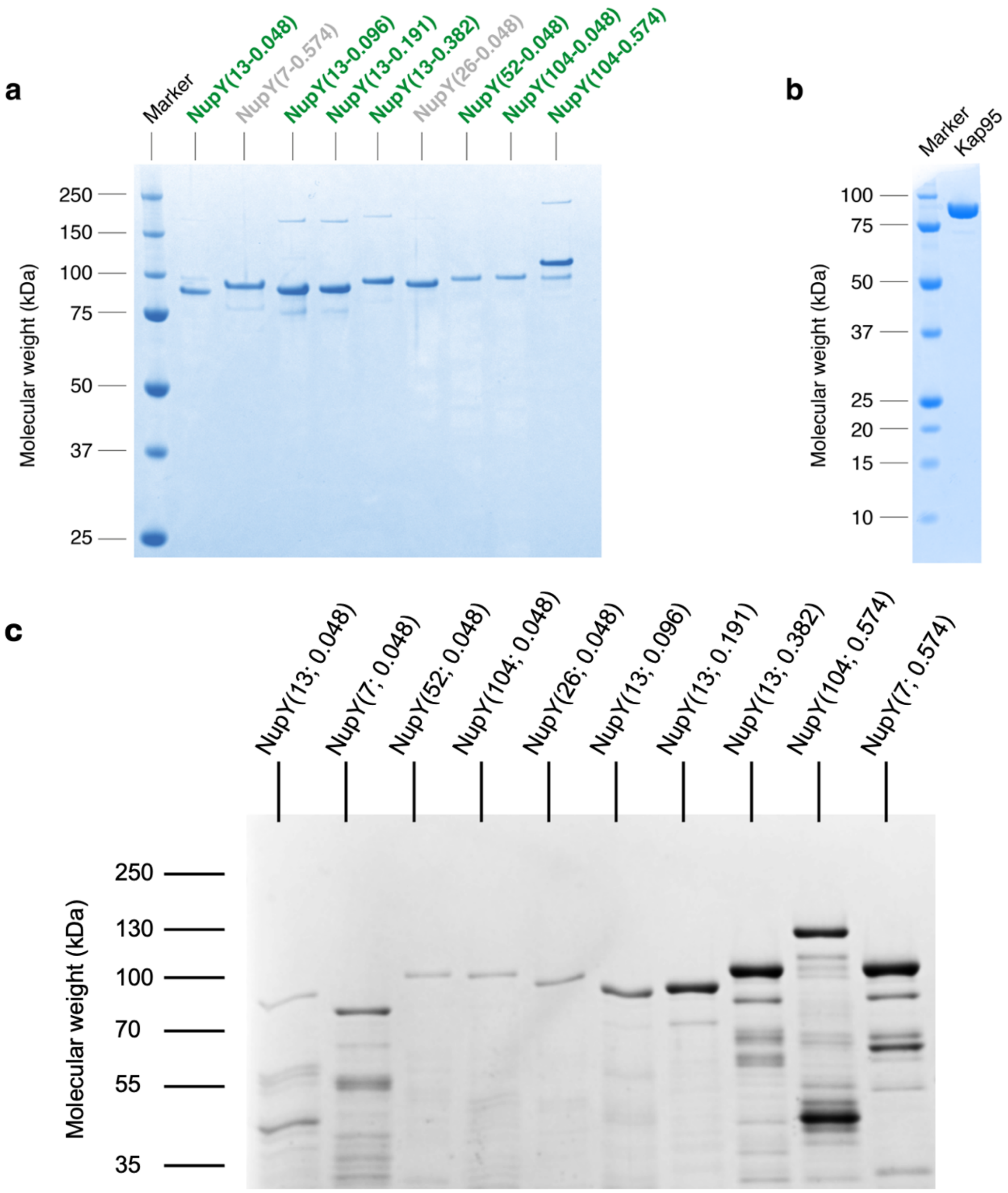

Supplementary Figure 25– SDS-PAGE gel of purified NupY variants and Kap95.
a) Coomassie-stained SDS-PAGE gel showing purified NupY variants running between ~80–120 kDa. Green labels indicate the NupY variants used for QCMD-D and LLPS experiments, whereas the grey ones (NupY(7;0.574) and NupY(26;0.048)) could not be used due to unsatisfactory expression levels. **b)** Coomassie-stained SDS-PAGE for Kap95 confirms the high purity of the Kap95 sample. **c)** Coomassie Brilliant Blue G250 SDS-PAGE gel of purified NupY variants used in Figure S11D.

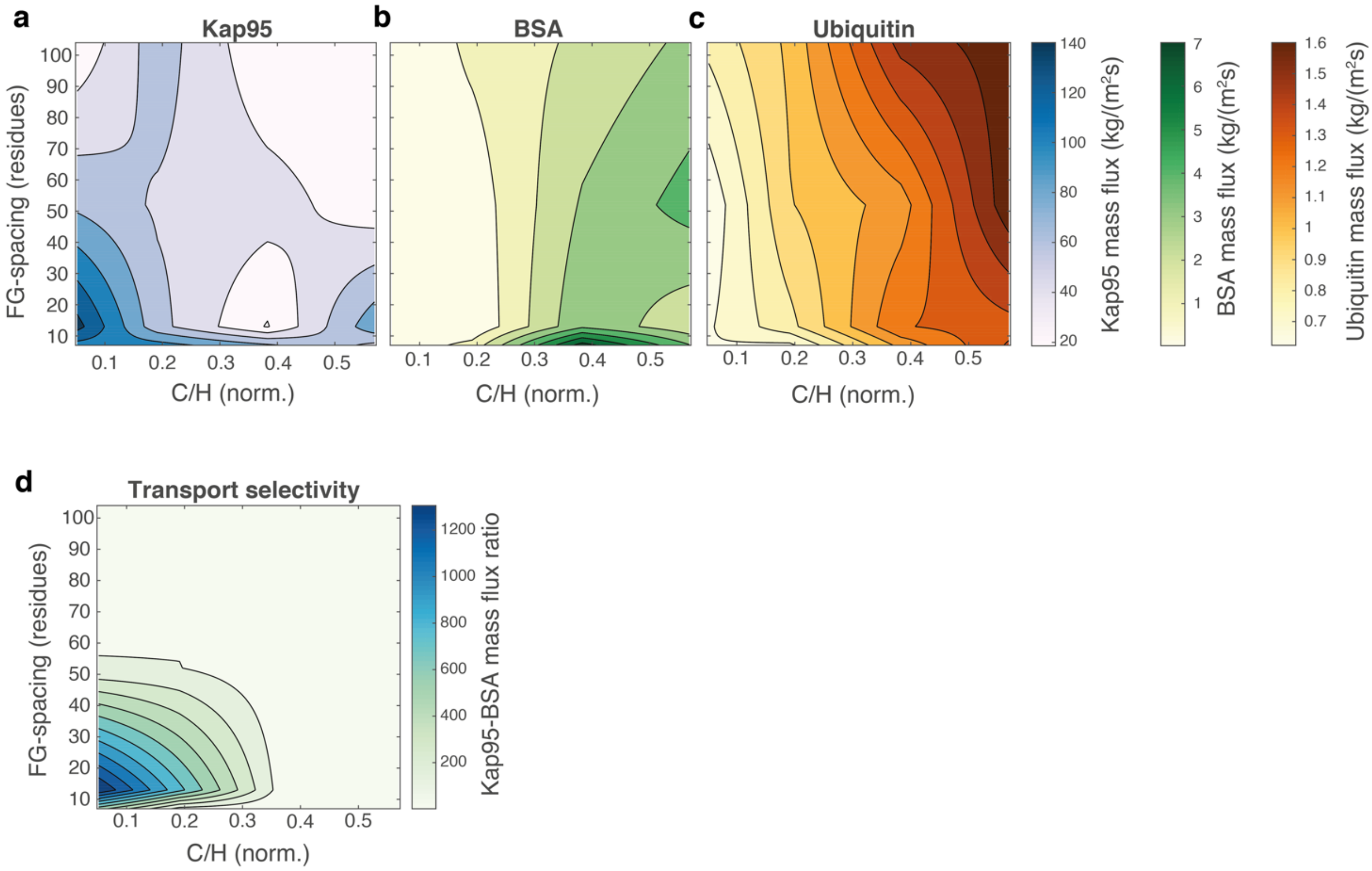

Supplementary Figure 26 – Contour graphs of cargo mass flux and NupY nanopore selectivity
a: Mass flux of Kap95 as a function of d_{FG} and C/H. A global maximum is clearly visible for the native GLFG-Nup average NupY(13;0.05). **b-c:** Mass fluxes of the inert proteins BSA and ubiquitin as a function of d_{FG} and C/H. The mass flux of BSA and ubiquitin increased notably with C/H. The barrier function of NupY-variant pores strongly decreased between C/H-values of 0.19 and 0.38. A local optimum exists for BSA (NupY(7;0.38)), due to a slightly attractive interaction between BSA and NupY(7;0.38) driven by cation-pi interactions **d:** Transport selectivity, defined as the ratio of the Kap95 and BSA mass flux, as a function of d_{FG} and C/H. We found an optimal transport selectivity for NupY(13;0.05), which corresponds to the native GLFG-Nup average of d_{FG} and C/H.

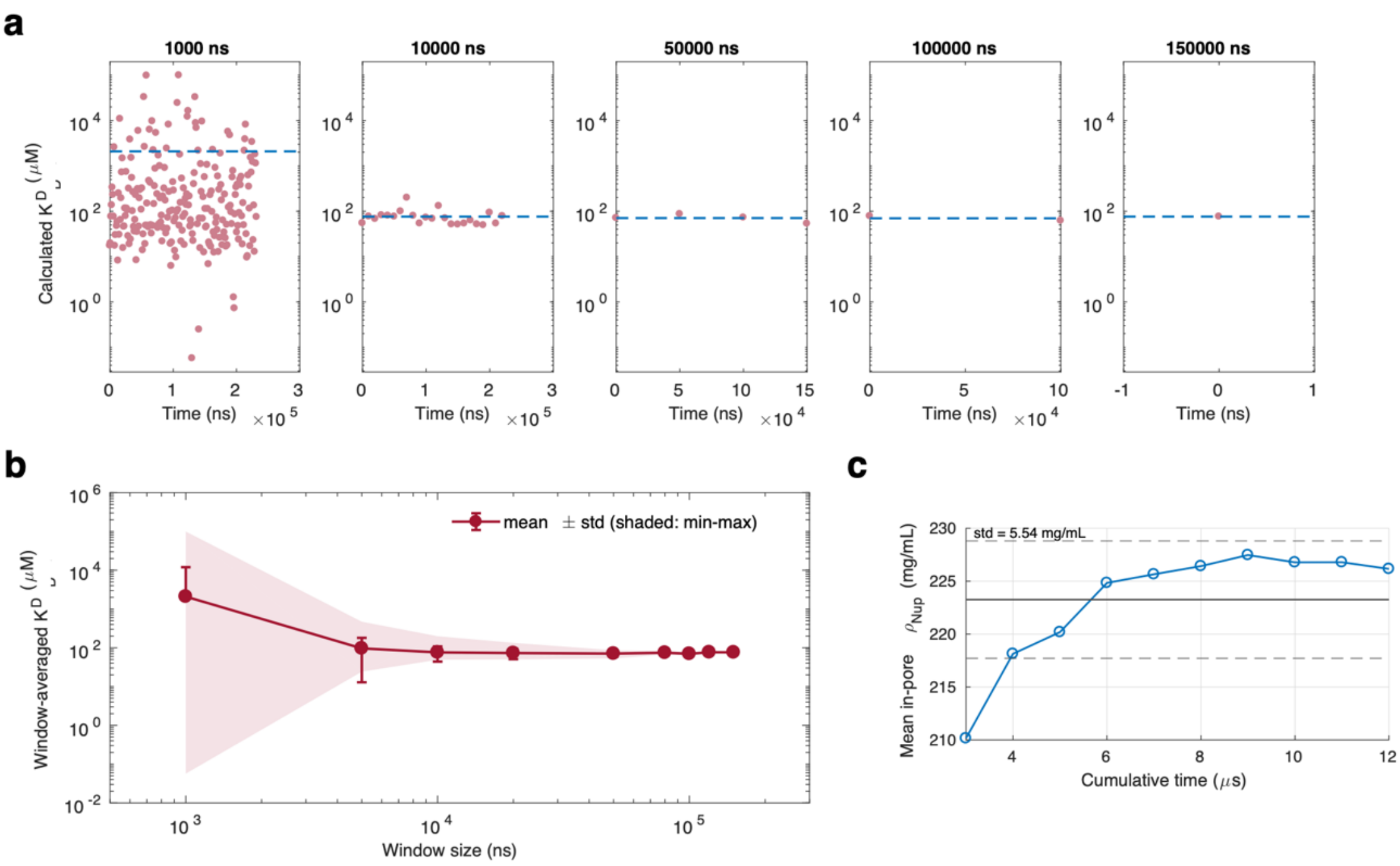

Supplementary Figure 27 – Equilibration of dissociation constant and in-pore protein density
a: Impact of block size on K_D-value for the NupY(13;0.05) variant. When the number of configurations used for the dissociation constant calculation is too small, the method will not converge to the true dissociation constant. This is in line with our earlier work[11], as well as the original study on the subvolume-based approach[12]. **b:** Mean and standard deviation in K_D as a function of total number of simulation frames. For the simulation times used throughout our binding simulations, the K_D-value statistically converges to the true value. **c:** Mean in-pore density as a function of cumulative simulation time for the NupY(13;0.05) variant. The mean density drifts by at most 2% in the first part of the simulation trajectory, and remains stable for the remainder.

Supplementary references

1. Schmidt, H. B. & Görlich, D. Nup98 FG domains from diverse species spontaneously phase-separate into particles with nuclear pore-like permselectivity. *Elife* **4**, e04251 (2015).
2. Milles, S. & Lemke, E. A. Single Molecule Study of the Intrinsically Disordered FG-Repeat Nucleoporin 153. *Biophys. J.* **101**, 1710–1719 (2011).
3. Xu, S. & Powers, M. A. In vivo analysis of human nucleoporin repeat domain interactions. *Mol. Biol. Cell* **24**, 1222–1231 (2013).
4. Yamada, J. *et al.* A Bimodal Distribution of Two Distinct Categories of Intrinsically Disordered Structures with Separate Functions in FG Nucleoporins. *Mol. Cell. Proteomics* **9**, 2205–2224 (2010).
5. Jafarinia, H., Van der Giessen, E. & Onck, P. R. Molecular basis of C9orf72 poly-PR interference with the β-karyopherin family of nuclear transport receptors. *Sci. Rep.* **12**, 21324 (2022).
6. Jafarinia, H., van der Giessen, E. & Onck, P. R. Phase Separation of Toxic Dipeptide Repeat Proteins Related to C9orf72 ALS/FTD. *Biophys. J.* **119**, 843–851 (2020).
7. Holehouse, A. S., Das, R. K., Ahad, J. N., Richardson, M. O. G. & Pappu, R. V. CIDER: Resources to Analyze Sequence-Ensemble Relationships of Intrinsically Disordered Proteins. *Biophys. J.* **112**, 16–21 (2017).

8. Ginell, G. M. & Holehouse, A. S. Analyzing the Sequences of Intrinsically Disordered Regions with CIDER and localCIDER BT - Intrinsically Disordered Proteins: Methods and Protocols. in (eds. Kragelund, B. B. & Skriver, K.) 103–126 (Springer US, 2020). doi:10.1007/978-1-0716-0524-0_5.
9. Peyro, M., Soheilypour, M., Nibber, V. S., Dickson, A. M. & Mofrad, M. R. K. FG nucleoporins feature unique patterns that distinguish them from other IDPs. *Biophys. J.* **120**, 3382–3391 (2021).
10. Peyro, M., Dickson, A. M. & Mofrad, M. R. K. Nucleoporins' exclusive amino acid sequence features regulate their transient interaction with and selectivity of cargo complexes in the nuclear pore. *Mol. Biol. Cell* **32**, ar31 (2021).
11. Fragasso, A. *et al.* Transport receptor occupancy in nuclear pore complex mimics. *Nano Res.* **15**, 9689–9703 (2022).
12. Jost Lopez, A., Quoika, P. K., Linke, M., Hummer, G. & Köfinger, J. Quantifying Protein–Protein Interactions in Molecular Simulations. *J. Phys. Chem. B* **124**, 4673–4685 (2020).
