# Supplementary material for: Spatial control of karyopherin binding avidity within NPC mimics revealed by designer FG-nucleoporins
Source: Nat Commun. 2026 Sep 28;17:11379. doi: 10.1038/s41467-026-77818-0 (PMC13737079; doi:10.1038/s41467-026-77818-0)
Supplement: Supplementary file 2 [file 41467_2026_77818_MOESM2_ESM.pdf]

nature portfolio

Peer Review File

Spatial control of karyopherin binding avidity within NPC mimics revealed by designer FG-Nucleoporins

Corresponding Author: Professor Patrick Onck

This file contains all reviewer reports in order by version, followed by all author rebuttals in order by version.

Attachments originally included by the reviewers as part of their assessment can be found at the end of this file.

Version 0:

Reviewer comments:

Reviewer #1

(Remarks to the Author)
Reviewer's Report for "Spatial control of karyopherin binding avidity within NPC mimics revealed by designer FG-Nups"

The study conducted by two stellar research groups extends their previos collaboration to present here a comprehensive study that explores how two key sequence-level properties—FG-repeat spacing (dFG) and charge-to-hydrophobicity ratio (C/H)—regulate the selective transport of karyopherins through the nuclear pore complex (NPC). Using a modular, synthetic FG-Nup scaffold (Nup-Y), the authors systematically vary dFG and C/H and characterize their effects on NTR binding, cargo partitioning, and phase behavior through a combination of biophysical experiments (QCM-D, partitioning, condensates) and coarse-grained molecular dynamics simulations. They provide convincing evidence that multivalent binding and entropic effects are modulated by these parameters, with results suggesting that native GLFG Nups may have evolved to balance Kap95 avidity and mobility optimally.

The paper is generally well written and well structured.

I have a few suggestions that, I think, would enhance the impact of the work.

Major Comments
1. Motivation and Scope of Parameter Space. The authors choose to vary FG spacing and C/H ratio but do not explicitly justify why other potentially relevant sequence features were excluded. Prior work (e.g., Peyro et al., Biophysical Journal, 2021. DOI: 10.1016/j.bpj.2021.06.031.) has shown that longest positive-like charge regions (lpLCRs) are a defining and functionally significant feature of FG-Nups. Could the authors briefly discuss whether parameters such as lpLCR presence, net charge distribution, or aromatic clustering could affect the conclusions?
In a separate study, Peyro et al. (Molecular Biology of the Cell. 2021. DOI:10.1091/mbc.E21-04-0161) further characterize how FG-Nup sequences differ fundamentally from general IDPs in their charge decoration, with implications for selective barrier formation. The current study could benefit from explicitly referencing these sequence design principles when discussing the synthetic protein architecture.

2. Clarification of Nup-X vs Nup-Y. The distinction between Nup-X (from Fragasso et al., 2021) and the newly introduced Nup-Y is not very clear. Please provide a clear comparison outlining how Nup-Y improves upon or differs from Nup-X. In addition, the explanation of how C/H is increased while keeping net charge fixed is confusing. Clarify what residue substitutions were used and how charge neutrality was maintained.

3. Selection of Variants. In the section titled "FG-spacing and C/H impact Kap95 selectivity," line 192 may have a grammatical issue and lacks a clear rationale for the selection of the seven variants studied. Were these chosen to span a physiological range or based on previous work? A table summarizing their properties (dFG, C/H, net charge, FG density) would improve transparency.

4. Entropic Effects and Discussion Consistency. Entropic repulsion is mentioned in the Introduction but not adequately

developed in the Results. The Discussion returns to the concept, particularly in the context of condensates and brush softness. It would be beneficial to connect the thermodynamic terms more explicitly to specific figures or datasets.

5. Trade-off between low Kd and reduced NTR mobility. The potential trade-off between low Kd (high affinity) and reduced NTR mobility is intriguing and could be elaborated, perhaps with reference to the agent-based modeling framework introduced in Azimi et al. (2013, PLoS ONE. DOI: 10.1371/journal.pone.0081741), which showed that an affinity gradient optimizes transport. Azimi et al. showed that spatial affinity gradients, with stronger FG-Nup-importin interactions at the nuclear basket, enhance directional transport. The authors could more explicitly tie their findings (e.g., the variation in Kap95 binding across synthetic gradients) to this concept of spatial encoding of avidity.
Minor Comments
• Some of the simulation results feel repetitive and could be consolidated to maintain reader engagement.
• Figures could benefit from clearer legends or visual summaries. For example, a 2D map of dFG vs C/H overlaid with Kap95 partitioning/selectivity would aid interpretation.
• Consider including implications for synthetic nanopore design or therapeutic delivery.
• A supplementary table with all synthetic sequences and their biophysical characteristics would be helpful.
• Line 481 is incomplete and should be revised.
• Line 515: “With FG-spacing…” is ambiguous—does increasing spacing cause the described effect or decreasing it?
• Lines 611–614 are unclear and should be rewritten for clarity.
• In the Discussion paragraph starting at line 560, it is not clear which data (e.g., brush, pore, condensate) are being referenced. Please add figure citations.

(Remarks on code availability)

Reviewer #3

(Remarks to the Author)
In the data, the Kap95 affinity for NupY was assessed using QCMD. NupY variants were physisorbed on the Au sensor surface until a similar density was obtained assessed via the changes in frequency. This is an incorrect approach as the frequency is a measure of hydrodynamic layer properties, and not of dry mass. The data must be seen in relation to the ratio of in changes of F/D, since similar frequencies could correspond to different dissipation change values, which could indicate very different densities and layer conformations. Indeed, the F/D values are reported in Figure c, and show that the layers were very different to each other although this is hard to tell as no errors and no statistical analysis has been done (QCMD signals vary quite a lot so repartitions and error must be shown).
What is MUTEG and why was it chosen for passivation? was this step really needed? It would be interesting to see data without this step as coadsorption of MUTEG can affect interactions with Kap95. Data in SI4b suggest there is significant adsorption of MUTEG, does Kap95 bind to these layers?
In order to analyse the kinetics, It is important that the adsorption process follows a single step mechanisms. This can be done by plotting F vs D for each overtone, and a single slope should be obtained (if the layer is dense all overtones should overlap). The fit should be done in the data region where the F vs D slope does not change. This needs to be done to verify the conclusions are correct,

(Remarks on code availability)

Reviewer #4

(Remarks to the Author)
In this paper, the authors made a series of FG-nups constructs varying FG distance and C/H. Compared with previous “NUP X’ designs, ‘NUP Y’ based variances allowed the researchers to systematically detect the parameter changing tendency varying FG distance and C/H ratio. In the paper, they applied biophysical measurements combined with coarse-grained simulations to explore a broad range of parameters regarding the distribution of FG nups inside nanopores and corresponding effect on transport receptors interactions and cargo transportation. Significantly, all the parameters achieved by different methods were consistent with each other and the authors gave reasonable explanations for outliers and exceptions.

The question I have is that since the paper shows good correlations between FG distance or C/H with Kap95 binding and cargo transportation, is it possible that the authors can predict or explain some natural nups behaviors or functions based on the results?

The other suggestion I have is that the whole story is not very reader friendly due to a broad range of statements made in the manuscripts. I made a table to combine all parameters in the paper (attached in the next page). So, I suggest adding a similar summary table or paragraph to guide the reader and for future references.

Overall, I think it’s good work and recommend for publication.

Minor points:

Figure 6f heatmaps missing LUTs.
Line 481: not yet finished sentences.
Figure 6: switch panel ‘d’ with ‘e’ and switch panel ‘f’ with ‘g’ for consistency

(Remarks on code availability)

Reviewer #5

(Remarks to the Author)
In the paper entitled, “Spatial control of karyopherin binding avidity within NPC mimics revealed by designer FG-Nucleoproteins” de Vries et al. explore the protein design space for nuclear pore complex proteins. In a series of elegantly designed experiments the authors clearly demonstrate the importance of protein sequence, FG motif spacing, and charged patterning for nuclear pore function and condensation. In particular, the authors manipulate the protein sequences of yeast nuclear pore proteins to create synthetic mutants which approximate properties of many native yeast NPC proteins (10-15 FG spaced repeats, ~0.5 Charged to uncharged). In keeping with expected behavior for nuclear pore proteins, in the presence of PEG, a crowding agent, mutant sequences which recapitulate wildtype parameters display propensity towards condensate formation and can recruit Kap95 (a protein critical for nuclear import). FRAP analysis highlights the relative mobility within the condensate. Molecular dynamics simulations of tested sequences further support the finding that sequence encodes density and specific affinity for cargo. These data help to establish important parameters governing the selective permeability of the nuclear pore assembly. I would support the publication of this manuscript in Nature Communications following some revisions.
Major concern 1: My understanding is that all the proteins sequence tested/simulated are synthetic. It would be useful to also test the closest native protein sequence from yeast (e.g. Nup116) to demonstrate if the mutant sequences are indeed reflective of native properties.
Major concern 2: For Figure 3, many of the conditions were performed in the presence of the crowding agent PEG8000 as such I am not confident how “specific” the recruitment of Kap95 into condensates. Please try in the absence of PEG to experimentally measure the affinity (do the proteins bind, at what KD, do they co-condense or aggregate together?), Data for supplemental figure 7 suggests some proportion of each of the variants is insoluble in the absence of PEG, and not “liquid-like”. These data would help support findings in figure 3.
Major concern 3: I was unable to find the location of the source code or simulation data in the text. Please emphasize the figshare location and further describe was included (code/data availability section etc.). Although substantial proportions of some of the code appear to be previously published, this does not address if changes have been made how it was etc.)
My minor concern comments are mostly stylistic. I found the naming convention for the mutants difficult to follow. Please consider a different more descriptive naming convention which reflects the similarity or difference to “wildtype” proteins. Another comment has to do with the figure titles. Most of the titles only state an aspect of the experiment and not why the experiment was done or what the results mean. For example, figure 4 “Mobility of Kap95 in NupY condensates” This title does not tell me why this experiment matters. Consider more descriptive figure titles.
Additional Minor points:
To help with interpretation of figure 1 could you color or name mutants shared on panel C with panel f and g and continue throughout.
In addition to the C/H ratios can you also calculate the isoelectric point for each of the mutants? This would help with interpretation of the condensation experiment results.
Please include the comparisons of NupY to well-studied non-yeast nuclear pore proteins. How conserved is this patterning.
Line 31 Should step-wise be one word? Similarly, Line 48 densely-filled two words? Consider adjusting words containing -.

(Remarks on code availability)
Source data files were poorly annotated (which one was the code?). Please consider adding more details to these sections for the final submission.

Version 1:

Reviewer comments:

Reviewer #1

(Remarks to the Author)
All points raised in my prior review have been satisfactorily resolved through the revisions and the added clarifications. The work makes a solid contribution and is ready for publication. I recommend that the manuscript be accepted for publication. - Mohammad Mofrad

(Remarks on code availability)

Reviewer #2

(Remarks to the Author)
Regarding Figure 2 and related QCMD data:

I acknowledge the effort made to improve the clarity of the results, though there are further clarification and work needed to make the statements fully transparent and true.
The authors should clarify in the early text the meaning of the nomenclature use, NypY (X;Y)... I see the description in the method section but that comes at the very end. So please add the definition either in the caption of the figure or in the main text at the start of the draft. Supplementary figures 5 and 6 should show not only the frequency but also the dissipation values. As the authors discuss, the dissipations are all well below 1, if so they could apply estimate the thicknesses of these layers assuming a given density for that protein layer. are these reasonable values? The authors state: Since higher hydration may lead to larger apparent frequency
shifts due to increased amount of coupled water molecules, the resulting protein coverage may be slightly overestimated for the more hydrated brushes. Actually the QCMD overtones contain that information. If the various overtones overlap then the mass or thickness can be directly compared as the layer are homogeneously distributes on the surface.
The authors should also apply some statistics to see if the values reported for F/D ratios in Figure 2b are statistically significant or not.
Line 208-209 should state (Supplementary Figure 7c).
Regarding Supplementary Figure 8, I would say there is some BSA binding, but the extent of it is way smaller than for Kap95... I think the scale used to plot the data is fooling the authors so they claim there is "completely lack of binding" for BSA.
In general, statistical tests are needed to claim: "In summary, the major trends with C/H and dFG were as follows: increases in dFG led to softer, more hydrated brushes which absorb less Kap95."

(Remarks on code availability)

Reviewer #3

(Remarks to the Author)
The authors have adequately addressed my comments and I have no further comments.

(Remarks on code availability)

Reviewer #4

(Remarks to the Author)
The authors have adequately addressed my major and minor concerns. I support the publication of this manuscript in its current form.

(Remarks on code availability)

Version 2:

Reviewer comments:

Reviewer #2

(Remarks to the Author)
The authors have properly addressed the comments raised

(Remarks on code availability)

Open Access This Peer Review File is licensed under a Creative Commons Attribution 4.0 International License, which permits use, sharing, adaptation, distribution and reproduction in any medium or format, as long as you give appropriate credit to the original author(s) and the source, provide a link to the Creative Commons license, and indicate if changes were made.
In cases where reviewers are anonymous, credit should be given to 'Anonymous Referee' and the source.
The images or other third party material in this Peer Review File are included in the article's Creative Commons license, unless indicated otherwise in a credit line to the material. If material is not included in the article's Creative Commons license and your intended use is not permitted by statutory regulation or exceeds the permitted use, you will need to obtain permission directly from the copyright holder.
To view a copy of this license, visit https://creativecommons.org/licenses/by/4.0/

RESPONSE TO REVIEWERS' COMMENTS

In black – reviewers' comments
In red – authors' responses

Reviewer #1

The study conducted by two stellar research groups extends their previous collaboration to present here a comprehensive study that explores how two key sequence-level properties—FG-repeat spacing (dFG) and charge-to-hydrophobicity ratio (C/H)—regulate the selective transport of karyopherins through the nuclear pore complex (NPC). Using a modular, synthetic FG-Nup scaffold (Nup-Y), the authors systematically vary dFG and C/H and characterize their effects on NTR binding, cargo partitioning, and phase behavior through a combination of biophysical experiments (QCM-D, partitioning, condensates) and coarse-grained molecular dynamics simulations. They provide convincing evidence that multivalent binding and entropic effects are modulated by these parameters, with results suggesting that native GLFG Nups may have evolved to balance Kap95 avidity and mobility optimally.

The paper is generally well written and well structured.

We thank the reviewer for the very positive appraisal of our work.

I have a few suggestions that, I think, would enhance the impact of the work.

Major Comments

1. Motivation and Scope of Parameter Space. The authors choose to vary FG spacing and C/H ratio but do not explicitly justify why other potentially relevant sequence features were excluded. Prior work (e.g., Peyro et al., Biophysical Journal, 2021. DOI: 10.1016/j.bpj.2021.06.031.) has shown that longest positive-like charge regions (lpLCRs) are a defining and functionally significant feature of FG-Nups. Could the authors briefly discuss whether parameters such as lpLCR presence, net charge distribution, or aromatic clustering could affect the conclusions?

We thank the reviewer for this question. Indeed, we focused on two orthogonal parameters (C/H, FG-spacing) while aiming to preserve net charge, and we did not explicitly scan other parameters.

Regarding LpLCR and charge patterning/net charges, we have now added two tables (Supplementary Tables 8 and 9) that contain (amongst others) the LpLCR lengths for the designer Nups as well as for the native yeast Nups. Our original rationale to not consider charge patterning as a control parameter in the design was twofold:

- On the one hand, we did not observe a high degree of charge patterning (by calculating the charge patterning parameter 'kappa', see Das & Pappu PNAS 2013 and Supplementary Table 9) in the GLFG-Nup collapsed domains. Since mutations and placement of charged residues

were randomly selected along the sequence in our design methods, we did not consider it necessary to investigate the charge patterning.

- Since the pool of disorder-promoting residues that increase the C/H of our designer Nups was limited, increasing C/H eventually would entail introducing additional charged residues. Since we mainly wanted to preserve the net charge of the protein, we therefore had to introduce both positive and negative charged residues, which eventually breaks any consistency in terms of LpLCRs present in the baseline design.

Regarding aromatic clustering: the overall number of non-Phenylalanine aromatic residues in FG-Nups was effectively zero and hence this was not further controlled for (see e.g. Supplementary Figure 1 for sequence histograms, or Figure 1 of Fragasso et al. Nat Comms. 2021). The main aromatic patterning effects therefore are inherent to the positioning of the FG-motifs in our designs, which is preserved between C/H mutants. We do note that the degree of patterning of the FG-motifs in our designs is higher than found in native FG-Nups and causes the NupY base design to be slightly more collapsed than e.g. Nup100 (which has the same length). We have added two figures with a comparison between NupY and Nup100 to the SI (Supplementary Figs. 3,4).

We now briefly discuss all these considerations in the main text (P4, L17-29).

In a separate study, Peyro et al. (Molecular Biology of the Cell. 2021. DOI:10.1091/mbc.E21-04-0161) further characterize how FG-Nup sequences differ fundamentally from general IDPs in their charge decoration, with implications for selective barrier formation. The current study could benefit from explicitly referencing these sequence design principles when discussing the synthetic protein architecture.

As also mentioned above, we now illustrate in the main text (P4, L17-29) similarities and differences between native FG-Nups and our designer Nups with respect to these parameters, and mention design considerations while citing more broadly from this and other studies.

2. Clarification of Nup-X vs Nup-Y. The distinction between Nup-X (from Fragasso et al., 2021) and the newly introduced Nup-Y is not very clear. Please provide a clear comparison outlining how Nup-Y improves upon or differs from Nup-X.

We have extended the explanation that was present (P3, L32-36) to make these differences more clear.

In addition, the explanation of how C/H is increased while keeping net charge fixed is confusing. Clarify what residue substitutions were used and how charge neutrality was maintained.

We have extended the methods section to reflect this point (P16, L11-16).

3. Selection of Variants. In the section titled “FG-spacing and C/H impact Kap95 selectivity,” line 192 may have a grammatical issue and lacks a clear rationale for the selection of the seven variants studied.

Were these chosen to span a physiological range or based on previous work? A table summarizing their properties (dFG, C/H, net charge, FG density) would improve transparency.

We corrected the grammatical issue in line 192 by rephrasing it. From all the designed sequences, we selected first variants that span either the C/H or dFG-axis while keeping the other parameter fixed at the native average (i.e., C/H of 0.05 while varying d_{FG}, and vice versa). Then, we selected also a combined 'extreme' mutant (NupY104-0.57) and additional variants to better sample the transition in physicochemical behavior of the FG-Nups (26 spacing, native C/H, 2x native C/H with native d_{FG}). Although we performed genetic optimization and purification for all variants mentioned above, purification was not successful for some of these variants (i.e. too low purity, etc). We have now mentioned this explicitly in the results section (P4, L42-45) and color-coded Supplementary Table 1 to indicate this more clearly.

4. Entropic Effects and Discussion Consistency. Entropic repulsion is mentioned in the Introduction but not adequately developed in the Results. The Discussion returns to the concept, particularly in the context of condensates and brush softness. It would be beneficial to connect the thermodynamic terms more explicitly to specific figures or datasets.

We simplified the language throughout the manuscript to more uniformly refer to entropic repulsion or other entropy-related terms as 'steric repulsion' which we feel covers better the effect that we are trying to describe. Indeed, entropy as a thermodynamic quantity can have multiple contributors which we did not explicitly characterize.

5. Trade-off between low Kd and reduced NTR mobility. The potential trade-off between low Kd (high affinity) and reduced NTR mobility is intriguing and could be elaborated, perhaps with reference to the agent-based modeling framework introduced in Azimi et al. (2013, PLoS ONE. DOI: 10.1371/journal.pone.0081741), which showed that an affinity gradient optimizes transport. Azimi et al. showed that spatial affinity gradients, with stronger FG-Nup-importin interactions at the nuclear basket, enhance directional transport. The authors could more explicitly tie their findings (e.g., the variation in Kap95 binding across synthetic gradients) to this concept of spatial encoding of avidity.

We thank the reviewer for pointing this work out to us. We now refer to more findings from the scientific literature (including this paper) when we postulate the important role of NTR avidity (P14, L14-16).

Minor Comments

• Some of the simulation results feel repetitive and could be consolidated to maintain reader engagement.

We acknowledge that the nanopore results in presence/absence of NTRs may come across as repetitive. However, we consider it important to show these separately since the presence of cargo molecules may affect the distribution of the FG-Nups.

• Figures could benefit from clearer legends or visual summaries. For example, a 2D map of dFG vs C/H overlaid with Kap95 partitioning/selectivity would aid interpretation.

We have now added the same data as contour graphs as a new Supplementary Figure 25, which can assist in more quantitatively illustrating the 2D scaling of the panels in Figure 7.

• Consider including implications for synthetic nanopore design or therapeutic delivery.

We appreciate the comment and mentioned this in the closing section.

• A supplementary table with all synthetic sequences and their biophysical characteristics would be helpful.

In line with comments by Reviewers 1 and 4 we added a table with relevant properties in Supplementary Table 8 and the exact sequences in Supplementary Table 1.

• Line 481 is incomplete and should be revised.

Indeed, the remainder of the sentence dropped out during manuscript writing, and is now added back in (P11L14).

• Line 515: “With FG-spacing…” is ambiguous—does increasing spacing cause the described effect or decreasing it?

We added the qualifier ‘increasing’ to the sentence (P11L50).

• Lines 611–614 are unclear and should be rewritten for clarity.

We have expanded the explanation on P13, L44-49.

• In the Discussion paragraph starting at line 560, it is not clear which data (e.g., brush, pore, condensate) are being referenced. Please add figure citations.

We have now clarified in the text that this pertains to the condensate experiments (P12L44).

Reviewer #2

In the data, the Kap95 affinity for NupY was assessed using QCMD. NupY variants were physisorbed on the Au sensor surface until a similar density was obtained assessed via the changes in frequency. This is an incorrect approach as the frequency is a measure of hydrodynamic layer properties, and not of dry mass.

We thank the reviewer for raising this point. We are well aware of the limitation of the QCM-D technique and that different layer viscosities may impact the frequency response and the apparent surface coverage.

Here is a list of points that address this issue:

1. We now added clarifying text at P5, L8-14 reporting on this limitation of the technique.
2. While it is true that different dissipation-to-frequency ratios indicate differences in brush hydration, the actual values are all within ~0.03×10^{-6} Hz^{-1}, and well below 0.4×10^{-6} Hz^{-1} (and at most ~0.06×10^{-6} Hz^{-1} for NupY(104;0.05)), a condition that is generally considered close to satisfying the Sauerbrey relation.
3. We assessed the robustness of the observed Kap95 binding to NupY(13;0.05) brushes of different densities. We analysed Kap95 binding to NupY(13;0.05) brushes that were formed under different concentrations, to achieve very different frequency shifts (from -42 to -115 Hz), see the added Supplementary Figure 6. Upon titrating Kap95 across similar concentration ranges of ~30 to 500 nM, we found very similar frequency responses, indicating that even such dramatic changes in brush density do not substantially impact the Kap95 binding response.

For these reasons, we believe these differences do not significantly impact the observed trends in Kap95 responses, which we found to be robust across large changes in brush density.

The data must be seen in relation to the ratio of in changes of F/D, since similar frequencies could correspond to different dissipation change values, which could indicate very different densities and layer conformations. Indeed, the F/D values are reported in Figure c, and show that the layers were very different to each other although this is hard to tell as no errors and no statistical analysis has been done (QCMD signals vary quite a lot so repartitions and error must be shown).

We added now more replicates in Figure 2c, which are consistent with the previously observed trends.

What is MUTEG and why was it chosen for passivation? was this step really needed? It would be interesting to see data without this step as coadsorption of MUTEG can affect interactions with Kap95. Data in SI4b suggest there is significant adsorption of MUTEG, does Kap95 bind to these layers?

MUTEG is a 1-mercapto-1-undecylte-tra(ethyleneglycol) molecule (as described in the Methods section). It is a short 380 Da molecule that is widely used in the field to passivate the remaining exposed gold surface after the FG-Nup coating step. It has been shown in the literature to exhibit virtually no

interactions with Kap95, including our previous work, see Supplementary Figure 5b in Fragasso et al., 2021 (https://doi.org/10.1038/s41467-021-22293-y). We refer the reviewer to existing literature where the same passivation protocol has been used and further described:

- Schoch, R. L., Kapinos, L. E. & Lim, R. Y. H. Nuclear transport receptor binding avidity triggers a self-healing collapse transition in FG-nucleoporin molecular brushes. *Proc. Natl. Acad. Sci. U. S. A.* **109**, 16911–16916 (2012)
- Kapinos, L. E., Schoch, R. L., Wagner, R. S., Schleicher, K. D. & Lim, R. Y. H. Karyopherin-centric control of nuclear pores based on molecular occupancy and kinetic analysis of multivalent binding with FG nucleoporins. *Biophys. J.* **106**, 1751–1762 (2014).
- Kapinos, L. E., Huang, B., Rencurel, C. & Lim, R. Y. H. Karyopherins regulate nuclear pore complex barrier and transport function. *J. Cell Biol.* **216**, 3609–3624 (2017).
- Wagner, R. S., Kapinos, L. E., Marshall, N. J., Stewart, M. & Lim, R. Y. H. Promiscuous binding of karyopherinβ1 modulates FG nucleoporin barrier function and expedites NTF2 transport kinetics. *Biophys. J.* **108**, 918–927 (2015).

We have now added these references in the main text.

In order to analyse the kinetics, It is important that the adsorption process follows a single step mechanisms. This can be done by plotting F vs D for each overtone, and a single slope should be obtained (if the layer is dense all overtones should overlap). The fit should be done in the data region where the F vs D slope does not change. This needs to be done to verify the conclusions are correct,

We thank the reviewer for the observation. We re-analysed the entire dataset and included more replicates (Figure 2j-k, Supplementary Table 6) to both ensure that the F vs D relation is linear, and that the extracted slope upon fitting a linear regression to the data is consistent across multiple harmonics.

The fits reported in Figure 2h-i and Supplementary Figure 9a-b, as well as the extracted parameters reported in Figure 2j-k and Supplementary Table 6 have consequently been updated. We updated the Method section at P19, L42-50, the main text at P5, L36-39, and added an example of such F vs D analysis in Supplementary Figure 9c-e.

Reviewer #3

In this paper, the authors made a series of FG-nups constructs varying FG distance and C/H. Compared with previous "NUP X' designs, 'NUP Y' based variances allowed the researchers to systematically detect the parameter changing tendency varying FG distance and C/H ratio. In the paper, they applied biophysical measurements combined with coarse-grained simulations to explore a broad range of parameters regarding the distribution of FG nups inside nanopores and corresponding effect on transport receptors interactions and cargo transportation. Significantly, all the parameters achieved by different methods were consistent with each other and the authors gave reasonable explanations for outliers and exceptions.

We thank the reviewer for the positive appraisal of our work.

The question I have is that since the paper shows good correlations between FG distance or C/H with Kap95 binding and cargo transportation, is it possible that the authors can predict or explain some natural nups behaviors or functions based on the results?

We thank the reviewer for this question. To some extent we already tried to draw up a mechanistic description of nuclear transport in our discussion section, where we hypothesize/predict from our findings that FxFG Nups and GLFG-Nups have distinct contributions. We have extended the discussion to refer more explicitly to how we arrived at this conclusion: namely by comparing the sequence properties of native FxFG and GLFG Nups (Figure 1d) to the scaling of the Kap-Nup binding Kd-values in Fig. 6b, as now explained on P14, L4-8, 9-11.

The other suggestion I have is that the whole story is not very reader friendly due to a broad range of statements made in the manuscripts. I made a table to combine all parameters in the paper (attached in the next page). So, I suggest adding a similar summary table or paragraph to guide the reader and for future references.

Each result section already contained a summarizing section, which focused mainly on mechanistic aspects. We have now augmented these with a number of clarifying/summarizing statements on the major trends with C/H and dFG for each set of sub-results (P6, L8-11; P7, L40-41, 45; P8, L42-44; P10, L16-17, 19-20; P11, L29-31).

Overall, I think it’s good work and recommend for publication.

We thank the reviewer for the recommendation to publish the work.

Minor points:

Figure 6f heatmaps missing LUTs.

The heatmap is positioned slightly above Fig. 6f due to space constraints in the panel figure.

Line 481: not yet finished sentences.

The sentence has now been completed.

Figure 6: switch panel ‘d’ with ‘e’ and switch panel ‘f’ with ‘g’ for consistency

We have switched the panels.

Reviewer #4

In the paper entitled, "Spatial control of karyopherin binding avidity within NPC mimics revealed by designer FG-Nucleoproteins" de Vries et al. explore the protein design space for nuclear pore complex proteins. In a series of elegantly designed experiments the authors clearly demonstrate the importance of protein sequence, FG motif spacing, and charged patterning for nuclear pore function and condensation. In particular, the authors manipulate the protein sequences of yeast nuclear pore proteins to create synthetic mutants which approximate properties of many native yeast NPC proteins (10-15 FG spaced repeats, ~0.5 Charged to uncharged). In keeping with expected behavior for nuclear pore proteins, in the presence of PEG, a crowding agent, mutant sequences which recapitulate wildtype parameters display propensity towards condensate formation and can recruit Kap95 (a protein critical for nuclear import). FRAP analysis highlights the relative mobility within the condensate. Molecular dynamics simulations of tested sequences further support the finding that sequence encodes density and specific affinity for cargo. These data help to establish important parameters governing the selective permeability of the nuclear pore assembly. I would support the publication of this manuscript in Nature Communications following some revisions.

We thank the reviewer for this positive appraisal and for supporting publication in Nature Communications.

Major concern 1: My understanding is that all the proteins sequence tested/simulated are synthetic. It would be useful to also test the closest native protein sequence from yeast (e.g. Nup116) to demonstrate if the mutant sequences are indeed reflective of native properties.

This is a valid question which we address in two ways:

- We provide a comparison between simulations of isolated NupY design variants (5) and the closest native yeast Nup, which is Nup100 (length-wise).
- We provide a comparison of phase separation data in presence/absence of Nup100 (see below as a response to major concern 2). Note that a simple comparison of phase separation propensity between NupY and Nup100 (pellets) was already present in Fig. 3a.

From the comparison of NupY baseline variants and Nup100 in our simulations, we observe the following two main differences (which are consistent with the results that we report in our study, and that do not affect the major findings).

1. FG-motifs, which act as 'stickers' and contribute to the condensation and brush behavior of the NupY-variants, are more evenly spaced in our NupY designs compared to the native Nup100 protein which has a similar total number of FG/GLFG motifs but placed more randomly. This causes the NupY variants to be slightly more collapsed.
2. The non-cohesive extended domain of NupY displays has some self-interactions with the collapsed domain in our simulations, whereas this does not occur in the native Nup100 protein. This may further explain why the NupY baseline design variants are somewhat more compact.

We now mention the above points in the results section (P4, L26-29) Additionally, we calculated several sequence parameters for the NupY-variants and native yeast FG-domains, such as charge patterning, like-charge region density, etc. (Supplementary Tables 8,9). This analysis highlighted that the baseline design reproduces key characteristics such as the length of like charge regions, and the overall charge patterning.

Major concern 2: For Figure 3, many of the conditions were performed in the presence of the crowding agent PEG8000 as such I am not confident how "specific" the recruitment of Kap95 into condensates. Please try in the absence of PEG to experimentally measure the affinity (do the proteins bind, at what KD, do they co-condense or aggregate together?),

We thank the reviewer for raising this point. Likewise, we considered the presence of the crowding agent in interpreting our results. Ideally, all experiments would have been performed in the absence of any additional crowding agents. However, it appeared to be impossible to reach sufficient concentrations of the different NupY constructs to perform the condensate experiments without a crowding agent, as the concentrations after the required dilution to remove the denaturant were below the critical concentration for all variants. An exception is the NupY(7;0.05) variant, which showed aggregation (Suppl. Fig. 10c). As speculated in the main text, we believe this to be mostly an effect of the inclusion of the hydrophilic extended C-terminal domain in the NupY design, as the pure FG-domain of Nup100 showed a much-reduced critical concentration and efficient condensate formation also in the absence of the crowding agent. Due to limitations of the protein expression levels (that could not be solved over the course of this long project), we thus are not able to perform the NupY condensate experiments in the absence of the crowding agent. We now mention this limitation in expression levels on P4, L42-45.

However, our data indicate that NupY condensates remain fluid/liquid in the presence of the crowding agent (see droplet merging in Fig. 3b, Kap95 diffusion in Fig. 4c), that PEG8000 is not accumulated in the condensates (absence of specific interactions with the crowding agent, Suppl. Fig. 11, and that distinct functional differences between the NupY variants under crowding conditions correlate well with the QCM-D results (Fig. 3c,f).

As an additional control, we have evaluated the behavior of the FG-domain of Nup100 in the presence and absence of the crowding agent (see Fig. R1 below). To visualize the condensates, we added a small amount (0.1%) of fluorescently labeled Nup100 FG-domain (by maleimide coupling of Alexa488 dye to the C-terminus) before inducing the condensate formation by dilution. In the absence of the crowding agent, the Nup100 FG-domain showed good wetting behavior for the *ibiTreat* surface of the coverslip (µ-Slide 15-well, ibidi, Germany), resulting in the growth of large and spread-out condensates (Fig. R1a, top, see below). In the presence of the crowding agent, the morphology changed to spherical condensates (Fig. R1a, bottom, see below). Likely this morphological change originates from the different growth behavior of the condensates. In the absence of the crowder, the growth of condensates occurs through the deposition of smaller droplets, which subsequently grow through Oswald ripening and merge on the coverslip surface, resulting in the formation of stretched-out

condensates. In the presence of the crowder, growth mostly occurs in the solution phase where large spherical condensates are pre-formed which then deposit on the surface. Despite the differences in the morphology, condensates of the Nup100 FG-domain remained liquid-like in the presence of the crowder and readily merged to form larger condensates (Fig. R1b).

We also did not observe differences in the uptake of Kap95 or cargoes in the presence and absence of the crowder (Fig. R1c). These data support the notion that PEG8000 acts as a passive crowder that promotes the formation of condensates but does not significantly alter their properties.

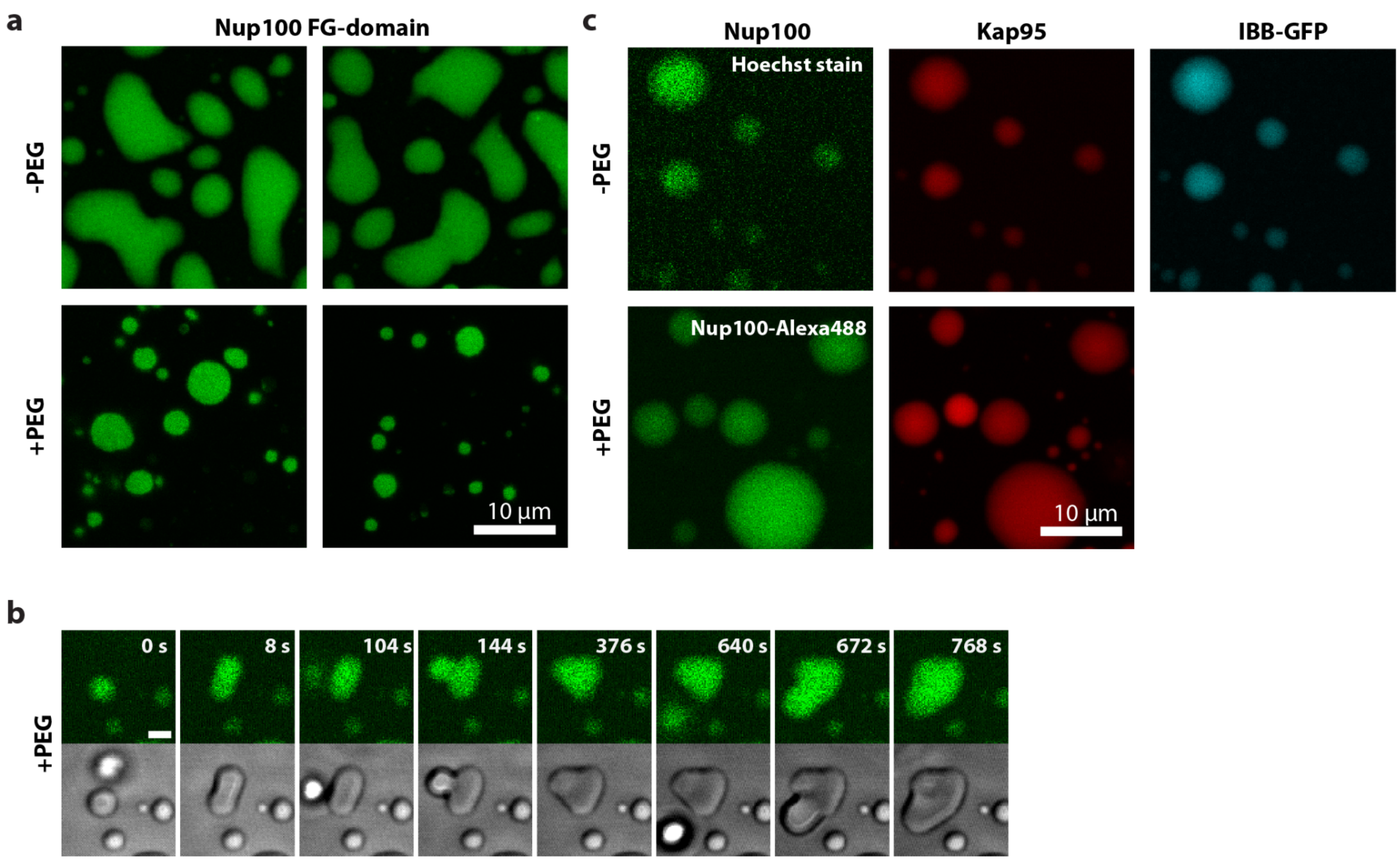

Fig. R1: Comparison of the liquid-liquid phase separation properties of the Nup100 FG-domain in the presence (+PEG) and absence (-PEG) of 10% w/v PEG8000. **a)** Condensate morphology of the Nup100 FG-domain in the absence (top) and presence (bottom) of crowding agent. Condensates are visualized by doping with a small amount (0.1%) of fluorescently labeled Nup100 (Nup100-Alexa488). **b)** Time lapse of condensates of the Nup100 FG-domain merging under crowding conditions (top: fluorescence of 0.1% Nup100-Alexa488, bottom: brightfield). **c)** Uptake of Kap95 into condensates of the Nup100 FG-domain in the absence (top) and presence (bottom) of crowding agent. Kap95 was added at a concentration of 1 µM. In the absence of the crowding agent, condensates were unspecifically stained using the dye Hoechst 33342 at a concentration of 10 µM, and the cargo molecule IBB-GFP was added at a concentration of 1 µM.

Data for supplemental figure 7 suggests some proportion of each of the variants is insoluble in the absence of PEG, and not “liquid-like”.

For the data shown in Supplementary Figure 7 of the original manuscript (now Supplementary Figure 10) we used centrifugation to separate the soluble and condensate phases by careful pipetting of the supernatant. However, there are limitations to the accuracy with which the pipetting can be performed. As such, there will be a residual amount of the soluble phase that remains with the “pellet” after pipetting, visible as faint bands for conditions where no crowding agent was used (left lanes in the absence of PEG in Suppl. Fig. 10a and b, faint bands for NupY(26:0.05) and NupY(52:0.05) in Suppl. Fig. 10d). Additionally, we note that in the absence of PEG, the NupY molecules could be on the border of the condensation threshold between soluble and 'liquid-like', and therefore the pellet size could be variable. The small pellet fraction could contain 'liquid-like' particles, as the buffer does not contain any phase separation disrupting chemicals (such as 1,6-hexanediol or SDS). As these amounts are small compared to the total protein amount, we do not consider them significant.

These data would help support findings in figure 3.

Major concern 3: I was unable to find the location of the source code or simulation data in the text. Please emphasize the figshare location and further describe was included (code/data availability section etc.). Although substantial proportions of some of the code appear to be previously published, this does not address if changes have been made how it was etc.)

We apologize for any unclarity. The source code, set-up files, etc. were uploaded before submission at https://github.com/Onck-group/designer-nups-kap-avidity. We have also added a copy of the repository to the source data file on figshare. Secondary simulation data (quantities derived directly from the raw trajectories) were already present in the material, and we have clarified further the nature/description of data in a metadata file.

My minor concern comments are mostly stylistic. I found the naming convention for the mutants difficult to follow. Please consider a different more descriptive naming convention which reflects the similarity or difference to “wildtype” proteins.

After a number of internal iterations, we did not come up with a more suitable name to highlight the quantitative nature of the C/H-value especially given that the ‘native’ NupY protein constitutes an average of sequence properties of multiple native Nups.

Another comment has to do with the figure titles. Most of the titles only state an aspect of the experiment and not why the experiment was done or what the results mean. For example, figure 4 “Mobility of Kap95 in NupY condensates” This title does not tell me why this experiment matters. Consider more descriptive figure titles.

We thank the reviewer for this comment and updated figure titles where possible.

Additional Minor points:

To help with interpretation of figure 1 could you color or name mutants shared on panel C with panel f and g and continue throughout.

The blue markers in Fig. 1c indicate the combinations of d_{FG} and C/H that were ultimately chosen for further studies (i.e. nanopore simulations, K_D simulations, etc). These do not correspond 1:1 to the variants shown in f and g, which correspond to 50 designs per FG-spacing or C/H-value, with more finely sampled intervals along the primary axis (dotted lines in panel 1c). We have clarified this further in the caption.

In addition to the C/H ratios can you also calculate the isoelectric point for each of the mutants? This would help with interpretation of the condensation experiment results.

In line with comments from Reviewers 1 and 3, we have added a new table to the SI with several common metrics based on the respective amino acid sequences of the collapsed domains (Supplementary Table 9), including the isoelectric point (pI).

Please include the comparisons of NupY to well-studied non-yeast nuclear pore proteins. How conserved is this patterning.

We have updated Figure 1, panels c-e with a number of human FG-Nups (Nup98, Nup153, Nup214). The properties of the full-length FG-domains of these Nups fall within our design space, and we note that hsNup98 and hsNup214 (Fig. 1d) have comparable FG-spacings and C/H-values as compared to the collapsed GLFG-Nups that we used to derive our NupY design from.

Line 31 Should step-wise be one word? Similarly, Line 48 densely-filled two words? Consider adjusting words containing -.

We thank the reviewer and have addressed any hyphenation issues throughout the manuscript.

Reviewer #4' Remarks on code availability: Source data files were poorly annotated (which one was the code?). Please consider adding more details to these sections for the final submission.

A code availability statement will be present in the final manuscript, and the code is available at https://github.com/Onck-group/designer-nups-kap-avidity.

RESPONSE TO REVIEWERS' COMMENTS

Remarks and rebuttal of final points of Reviewer #2:

Regarding Figure 2 and related QCMD data:
I acknowledge the effort made to improve the clarity of the results, though there are further clarification and work needed to make the statements fully transparent and true.

We thank the reviewer for acknowledging the improvements made to the manuscript.

- The authors should clarify in the early text the meaning of the nomenclature use, NypY (X;Y)… I see the description in the method section but that comes at the very end. So please add the definition either in the caption of the figure or in the main text at the start of the draft.

We thank the reviewer for the suggestion. We would like to refer the reviewer to lines 38-40, page 3, where we state:

'To distinguish between the different variants, we label each variant by its spacer length and C/H-value, e.g., 'NupY(13;0.05)' for the NupY variant with dFG=13 and C/H=0.05',

and to lines 45-48 at page 4 (at the beginning of the QCM-D session), where we write:

'The final set of proteins includes the template protein NupY(13;0.05), the FG-spacing variants NupY(52;0.05) and NupY(104;0.05), the C/H variants NupY(13;0.09), NupY(13;0.19) and NupY(13;0.38), as well as a high C/H variant with large FG-spacing, NupY(104;0.57) (Supplementary Table 1).'

- Supplementary figures 5 and 6 should show not only the frequency but also the dissipation values.

We now added dissipation values to Supplementary Figure 7 (previously 6).

As the authors discuss, the dissipations are all well below 1, if so they could apply estimate the thicknesses of these layers assuming a given density for that protein layer. Are these reasonable values?

We thank the reviewer for the suggestion. We estimated the harmonic spread of the frequency plateaus for multiple NupY variants and replicates, and found the error to be small (<3%) (Supplementary Figure 6a-b). We estimated the layer heights using the Sauerbrey equation and, assuming proper layer hydration (ρ=1 g/cm^3), found them to be in a reasonable range of 5.9-8.3 nm (Supplementary Figure 6c).

We now also added this point in the main text on page 5 and further details in the Methods section.

- The authors state: Since higher hydration may lead to larger apparent frequency shifts due to increased amount of coupled water molecules, the resulting protein coverage may be slightly overestimated for the more hydrated brushes. Actually the QCMD overtones contain that information. If the various overtones overlap then the mass or thickness can be directly compared as the layer are homogeneously distributes on the surface.

We thank the reviewer for the suggestions. We implemented this harmonic analysis into Supplementary Figure 6, updated the Methods section, modified the main text on page 5 accordingly.

- The authors should also apply some statistics to see if the values reported for F/D ratios in Figure 2b are statistically significant or not.

We now performed statistical tests (one-way ANOVA) and Tukey's HSD on the dataset reported in Figure 2c, which add more precision to the strength of our claims. We modified the Figure 2 caption and Methods accordingly.

- Line 208-209 should state (Supplementary Figure 7c).

We corrected now the misreferencing of Supplementary Figure 7c (now 8c).

- Regarding Supplementary Figure 8, I would say there is some BSA binding, but the extent of it is way smaller than for Kap95... I think the scale used to plot the data is fooling the authors so they claim there is "completely lack of binding" for BSA.

We rescaled the plot in the figure (now Supplementary Figure 10) to better show the minor frequency shift upon BSA binding and corrected the text accordingly. We also included more replicates for the different mutants to strengthen our conclusions.

In general, statistical tests are needed to claim: "In summary, the major trends with C/H and dFG were as follows: increases in dFG led to softer, more hydrated brushes which absorb less Kap95."

We now performed statistical tests (one-way ANOVA, Tukey's HSD) and ordinary least-squares logarithmic regression for the Kap95 binding parameters (Figure 2j-k). We evaluated the statistical significance of the latter based on the slope of the fitted logarithmic model due to the limited replicate numbers for individual variants. We modified the main text, Figure 2 caption, and Methods accordingly.

REVIEWER 4, ATTACHMENT 1

**Experiment**	**d_{FG} increase**<br>**Parameter trend**	**C/H increase**<br>**Parameter trend**	**Exception**
Single chain simulation	Stroke radius: Not Significant	Stroke radius: increase	d_{FG}=7 has very small stroke radius
QCM-D	Dissipation-to-frequency ratio: increase	Polymer extension: increase	
	Kap95 binding: decrease	Kap95 binding: k_D no change; Faster kinetics	Binding increase in NupY(104;0.57) VS NupY(104; 0.05)
	$t_{1/2}$ decrease, Δf max decrease	$t_{1/2}$ decrease, Δf max decrease	
Condensation	Saturation concentration: increase	C sat: NS	C_{sat} decrease in NupY(104;0.57) VS NupY(104; 0.05)
	Kap95 partitioning decrease	Kap95 partitioning: NS	Significant Kap$_{95}$ partitioning in large d_{FG}<br>As high enough FG in condensation in NupY (104; 0.05) not like in QCM-D for kap95 partitioning
Cargo uptake	30 kDa: steep decrease	30 kDa: NS	C/H > =0.38, sharp decreased large cargo uptake<br>As high C/H results in dense network
	90 kDa: less steep decrease	90 kDa: NS	
Frap experiment	Kap95 diffusion: low	Kap95 diffusion: low	
Nano-pore modeling	FG density: decreases<br>Nup density: NS	FG mass distribution: decrease	
Single molecule binding simulation	Kap95 binding: scale down with FG number<br>K_D: increase	Kap95 binding: decrease<br>K_D: increase	
Simulation on multi-valent interaction on individual molecules	Kap95 binding density: decrease<br>Kap95 localization: in the pore center in large d_{FG}	Kap95 binding density: decrease<br>Kap95 localization: more homogeneous	1. C/H = 0.57, Kap95 in the center.<br>2. An optimum in Kap95 mass flux and transport selectivity occurred for NupY(13;0.05)
	BSA binding region and density: NS	BSA density: increase	Barrier functionality strongly decreased beyond a C/H of 0.19
	Ubiquitin binding region and density: NS	Ubiquitin density: increase	
	FG density decrease	FG in density decrease	
